# Engineering Extracellular Vesicles for Anti‐Aging Therapy: Mechanisms, Applications, and Perspectives

**DOI:** 10.1111/acel.70607

**Published:** 2026-06-23

**Authors:** Xian Huang, Qiujie Li, Guofang Tao, Xulin Gan, Jiangjie Lu, Sergei Krasny, Liyun Shi

**Affiliations:** ^1^ College of Life and Environmental Sciences Hangzhou Normal University Hangzhou China; ^2^ Key Laboratory of Artificial Organs and Computational Medicine in Zhejiang Province, Institute of Translational Medicine Zhejiang Shuren University Hangzhou China; ^3^ Laboratory & Equipment Management Department Hangzhou Normal University Hangzhou China; ^4^ School of Public Health Zhejiang Chinese Medical University Hangzhou China; ^5^ N. N. Alexandrov National Cancer Centre of Belarus Minsk Belarus; ^6^ Yuhang Institute of Medical Science Innovation and Transformation Hangzhou Zhejiang China

**Keywords:** aging, clinical translation, extracellular vesicles, targeted delivery

## Abstract

Aging is a multifactorial process driven by interconnected hallmarks, including chronic inflammation, mitochondrial dysfunction, genomic and epigenetic alterations, and dysregulated intercellular communication. Extracellular vesicles (EVs), naturally derived nanoscale membrane vesicles capable of transporting diverse bioactive cargoes across tissues and biological barriers, have emerged as a highly promising platform for regenerative and anti‐aging therapeutics. In this review, we systematically summarize the multifaceted anti‐aging mechanisms of EVs, including suppression of the senescence‐associated secretory phenotype (SASP), remodeling of the immune microenvironment, mitochondrial restoration and metabolic reprogramming, DNA damage repair, epigenetic modulation, recovery of proteostasis, activation of regenerative signaling pathways, and cross‐organ communication‐mediated rejuvenation. Beyond mechanistic insights, we integrate the targeting biology and cellular entry properties of EVs, encompassing natural tropism determinants, engineered targeting strategies, biodistribution profiles, receptor‐ligand interactions, intracellular trafficking, and subcellular cargo release. Unlike previous reviews focusing on a single EV source or isolated pathways, we establish a comprehensive framework connecting molecular mechanisms with delivery engineering, tissue targeting, biosafety assessment, scalable manufacturing, and clinical translation. We address major technical bottlenecks limiting EV therapeutics—including EV heterogeneity, suboptimal delivery efficiency, endosomal degradation, and the lack of standardized quality‐control frameworks—while highlighting emerging solutions such as bioengineered EVs, hybrid vesicle platforms, biomaterial‐assisted delivery systems, and ultrasound‐enhanced targeting technologies. By bridging fundamental biology, nanomedicine engineering, and clinical translation, this review provides a strategic roadmap for the development of next‐generation precision anti‐aging nanotherapeutics with systemic regulatory capacity, translational feasibility, and broad clinical potential.

## Graphical abstract

This review systematically elucidates the multifactorial anti‐aging mechanisms of extracellular vesicles (EVs). By bridging fundamental biology with advanced bioengineering, scalable manufacturing, and clinical translation, it establishes a comprehensive framework for developing next‐generation EVs‐based precision nanotherapeutics to combat systemic aging.

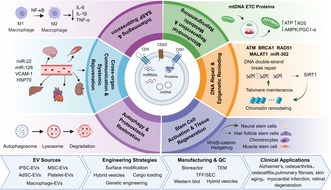

## 1 Introduction

Aging is a complex and systemic biological process involving multiple interconnected pathways and regulatory networks across molecular, cellular, tissue, and organismal levels. It is characterized by a series of conserved hallmarks, including genomic instability, telomere attrition, epigenetic alterations, loss of proteostasis, mitochondrial dysfunction, cellular senescence and the senescence‐associated secretory phenotype, stem cell exhaustion, deregulated nutrient sensing, altered intercellular communication, and chronic low‐grade inflammation (Childs et al. 2017; Guo et al. 2026; Kennedy et al. 2014; Lopez‐Otin et al. 2013, 2023). Collectively, these age‐associated alterations contribute to the onset and progression of disorders affecting the cardiovascular, neurological, musculoskeletal, metabolic, and renal systems, ultimately resulting in impaired tissue regenerative capacity, aggravated immunosenescence, and increased metabolic dysfunction in aging and multimorbid populations (Childs et al. 2017; Kennedy et al. 2014; Lopez‐Otin et al. 2023). Although small molecules, lifestyle interventions, and organ‐specific therapies have shown therapeutic benefits in certain contexts, their ability to comprehensively modulate the complex aging network remains limited due to the multifactorial and multisystem nature of aging. Moreover, current biomarkers of biological aging, including epigenetic clocks, inflammatory proteomic signatures, and functional indicators, still lack standardized and clinically actionable evaluation frameworks, thereby restricting cross‐study comparability and translational implementation. Therefore, there is an urgent need to develop therapeutic strategies capable of broadly correcting aberrant aging‐associated signals across tissues and organ systems. Importantly, such interventions must also exhibit robust manufacturing scalability, safety, and translational feasibility to address the growing clinical and public health challenges posed by population aging (Childs et al. 2017; Kennedy et al. 2014; Lopez‐Otin et al. 2013).

EVs are a heterogeneous population of nanosized particles naturally secreted by both prokaryotic and eukaryotic cells and enclosed by a lipid bilayer membrane. Acting as critical mediators of intercellular communication, EVs transfer diverse bioactive cargoes, including proteins, lipids, mRNAs, and non‐coding RNAs, to recipient cells. Based on their size, biogenesis pathways, and biological characteristics, EVs are generally classified into three major subtypes: exosomes (30–150 nm), microvesicles (100–1000 nm), and apoptotic bodies (1–5 μm) (Gyorgy et al. 2011; Kumar et al. 2024). Exosome biogenesis is a tightly regulated intracellular process that originates within the endosomal system. It is initiated by the inward budding of the limiting membrane of early endosomes, leading to the formation of intraluminal vesicles (ILVs) within multivesicular bodies (MVBs). The sorting and packaging of cargo during this process are primarily regulated through two major mechanisms: the Endosomal Sorting Complex Required for Transport (ESCRT)‐dependent pathway and the ESCRT‐independent pathway (Babst 2011; Horbay et al. 2022; Wei et al. 2021). Following maturation, MVBs are transported along the cytoskeleton toward the plasma membrane, a process primarily regulated by Rab family GTPases (Hyenne et al. 2018; Liu et al. 2025; Wei et al. 2021). Subsequently, MVBs dock and fuse with the plasma membrane through SNARE complex‐mediated membrane fusion, resulting in the release of exosomes into the extracellular space (Fukuda 2025; Liu, Liu, et al. 2023; Yang et al. 2019). These vesicles carry characteristic surface markers, including tetraspanins, membrane transport proteins, and biogenesis‐associated proteins, which not only protect their cargoes from enzymatic degradation but also influence their biodistribution, cellular targeting, and biological functions within complex physiological environments (Skowyra et al. 2024).

The therapeutic properties of EVs are intrinsically influenced by their parental cell origin, thereby providing a versatile cell‐free therapeutic platform for regenerative medicine and anti‐aging interventions. The spectrum of therapeutically relevant EVs has expanded considerably beyond mesenchymal stem cell (MSC)‐derived vesicles to include EVs originating from immune cells and blood components. For instance, EVs derived from M2‐polarized macrophages and neural stem cells exhibit potent anti‐inflammatory and neuroregenerative properties, whereas platelet‐derived EVs (PEVs) are enriched in growth factors that promote wound healing, angiogenesis, and tissue repair (Antich‐Rossello et al. 2021; Madhu et al. 2024). A major paradigm shift in the field has been the emergence of organelle‐derived therapeutics, particularly mitochondria‐derived vesicles (MitoEVs), which are capable of transferring functional mitochondrial components and metabolic enzymes to recipient cells, thereby restoring oxidative phosphorylation and ameliorating pathological conditions ranging from lung injury to metabolic syndrome (Ferrucci et al. 2024; Wu et al. 2024; Zhou et al. 2023). In addition, recent advances in bioengineering have led to the development of bacterial EVs and artificial cell‐derived vesicle mimetics, which provide highly customizable and scalable alternatives to naturally derived vesicles with improved production yield and engineering flexibility (Duan et al. 2024; Robinson et al. 2025). Compared with whole‐cell therapies, these diverse EV populations exhibit superior biosafety profiles, with minimal risks of tumorigenicity, immune rejection, or vascular occlusion. In addition, EVs demonstrate greater storage stability and possess the unique capacity to penetrate complex biological barriers, including the blood–brain barrier. Collectively, these advantages position EVs as a promising next‐generation platform for precision nanomedicine in the treatment of aging and age‐related diseases (Kumar et al. 2024).

While conventional therapeutic strategies are often constrained by single‐target mechanisms and safety concerns, including tumorigenicity and immune‐related adverse effects (Conboy et al. 2005; Mehdipour et al. 2020; Robbins and Morelli 2014; Sahoo and Losordo 2014; Villeda et al. 2014), EVs provide a promising cell‐free alternative characterized by low immunogenicity, high engineering flexibility, and the ability to modulate multiple signaling pathways simultaneously (Kamerkar et al. 2017; Mager et al. 2013; Tkach and Thery 2016; Wilkinson et al. 2012; Xia et al. 2022). Nevertheless, the clinical translation of EV‐based therapeutics remains substantially hindered by several biological and technical challenges, including source heterogeneity, rapid clearance by the reticuloendothelial system, inefficient endosomal escape, insufficient manufacturing standardization, and the lack of comprehensive long‐term safety evaluation frameworks. Therefore, this review focuses on two major themes: the mechanisms underlying EV‐mediated anti‐aging effects and the targeting and cellular entry properties of EVs.

Specifically, we summarize the multifaceted anti‐aging functions of EVs, including immunomodulation, mitochondrial restoration and metabolic reprogramming, genetic and epigenetic information transfer, regulation of proteostasis and autophagy lysosomal pathways, and stem cell‐mediated regenerative signaling. In parallel, we systematically discuss the targeting biology of EVs, encompassing natural and engineered targeting determinants, delivery routes and biodistribution patterns, receptor–ligand interaction networks, microenvironmental influences, cellular uptake and intracellular trafficking, as well as cargo release and subcellular localization. Furthermore, we integrate current evaluation frameworks related to pharmacokinetics, biosafety, toxicology, and quality attributes, with the aim of facilitating interdisciplinary collaboration and accelerating the clinical translation of EV‐based anti‐aging therapeutics.

In recent years, a substantial number of reviews have been published in the field of EVs. However, most existing studies primarily focus on a specific EV source, such as mesenchymal stem cell‐derived exosomes, individual signaling pathways, or particular categories of aging‐related diseases. Other reviews mainly emphasize engineering modifications or drug delivery strategies, while many regard EVs merely as passive regulators of specific pathological phenotypes. In contrast, comprehensive frameworks that systematically integrate the diverse anti‐aging mechanisms of EVs remain relatively scarce. In particular, the coordinated roles of EVs in immune regulation, mitochondrial restoration and metabolic reprogramming, genetic and epigenetic information transfer, proteostasis and autophagy–lysosomal regulation, as well as stem cell‐mediated regenerative signaling, have rarely been discussed within a unified anti‐aging paradigm.

The present review differs substantially from previous reviews in both conceptual scope and translational emphasis, with its unique value primarily reflected in two major aspects. First, rather than merely summarizing the beneficial effects of EVs in specific aging phenotypes, we systematically examine the entire mechanistic cascade underlying EV‐mediated anti‐aging functions, including cellular entry pathways, intracellular trafficking processes, subcellular cargo release, and their downstream biological effects. This integrated connection between EV targeting biology, intracellular dynamics, and functional outcomes has rarely been comprehensively discussed in previous reviews.

Second, unlike most existing reviews that only briefly address translational challenges in the concluding section, we place biosafety evaluation, quality control, pharmacokinetic assessment, and manufacturing standardization at the same level of importance as fundamental mechanistic studies. In particular, we provide an in‐depth discussion of the biosafety and translational risks of EV‐based therapeutics and further propose a reference framework for evaluating their quality attributes and clinical applicability. Collectively, this review aims to bridge fundamental EV biology with translational nanomedicine, thereby providing a more comprehensive theoretical and practical foundation for the development of next‐generation anti‐aging therapeutics.

In addition, there are few existing reviews that can take into account the targeting strategies of natural EVs and engineered EVs at the same time. There is also a lack of integrated analysis of the interaction between receptor‐ligand networks, microenvironment factors, and in vivo distribution differences caused by different administration routes. This review systematically summarizes the above contents, and further connects natural targeting determinants, engineered modification strategies, drug delivery routes and biodistribution, receptor‐ligand interaction networks and their microenvironment regulation, cell uptake and intracellular transport, and cargo release and subcellular localization into a coherent knowledge chain.

**FIGURE 1:**
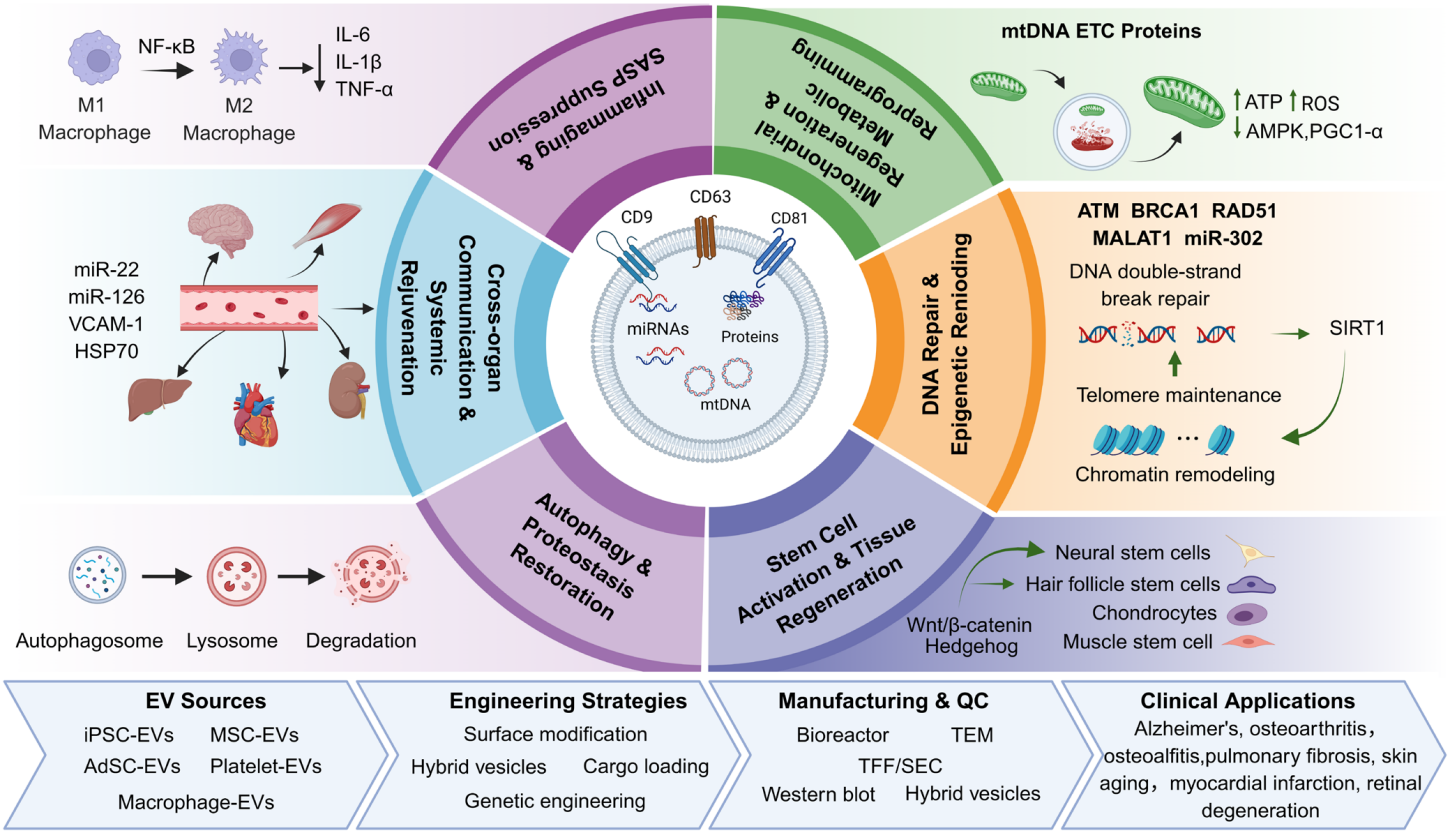
Schematic overview of EV based anti‐aging strategies and clinical translation. Created in httpsBioRender.

Overall, this review is not intended as a repetitive summary of the existing literature, but rather as an effort to bridge the critical gap between mechanistic research, bioengineering innovation, and clinical translation. Specifically, we aim to move beyond the descriptive observation that “EVs are effective in aging models” toward a more comprehensive understanding of how EVs can be safely, efficiently, and controllably developed as anti‐aging therapeutics. By integrating perspectives spanning fundamental mechanisms, targeting biology, engineering strategies, biosafety evaluation, and translational medicine, this review provides a conceptual framework that combines both theoretical depth and practical translational value for basic researchers, bioengineers, and therapeutic developers. The multifunctional roles, engineering strategies, and translational applications of EVs in anti‐aging therapy are schematically illustrated in Figure 1.

## 2 Biological Characteristics of EVs

### 2.1 Definition and Biological Properties

EVs are nanosized lipid bilayer‐enclosed structures secreted by nearly all cell types under both physiological and pathological conditions. They are widely distributed in various biological fluids, including blood, urine, saliva, and cerebrospinal fluid. As critical mediators of intercellular communication, EVs encapsulate and transport diverse bioactive cargoes, including proteins, lipids, nucleic acids, and metabolites, thereby regulating gene expression, signaling pathways, and biological functions in recipient cells (Chitti et al. 2024; Welsh et al. 2024; Yu et al. 2023). According to the latest guidelines of the International Society for EVs (MISEV2023), EVs are broadly classified into three major subtypes based on their biogenesis pathways and size characteristics: exosomes (30–150 nm), which originate from the endosomal pathway; microvesicles, also referred to as ectosomes (100–1000 nm), which are generated through direct outward budding of the plasma membrane; and apoptotic bodies (> 1000 nm), which are produced during programmed cell death. This biological heterogeneity enables EVs to participate in a wide range of physiological and pathological processes, including the maintenance of tissue homeostasis, modulation of disease progression, and mediation of therapeutic responses (Konig and McBride 2024).

### 2.2 Molecular Markers and Identification

Accurate characterization and identification of EVs constitute the foundation for both basic research and translational applications in the field. According to the MISEV guidelines and recent technological advances, EV identification primarily relies on the detection of specific combinations of protein markers. Among these, CD9, CD63, and CD81, which belong to the transmembrane tetraspanin family, are considered the most widely recognized positive surface markers of EVs and are extensively used in immunocapture, flow cytometry, and vesicle characterization assays (Bonner et al. 2024; von Lersner et al. 2024; Welsh et al. 2024). Because exosomes originate from MVBs, they are typically enriched in proteins associated with the endosomal sorting complex required for transport machinery, including TSG101, ALIX, and syntenin‐1. In addition, heat shock proteins (HSPs) are commonly used as representative cytosolic markers of EVs. To ensure the purity and reliability of EV isolation, it is equally important to assess negative markers, such as the endoplasmic reticulum‐associated protein calnexin, the Golgi apparatus marker GM130, and the mitochondrial protein cytochrome c, thereby excluding potential contamination from cellular debris, organelles, or cell lysates (Welsh et al. 2024).

### 2.3 Biogenesis and Secretion Regulation

The biogenesis of EVs is a highly coordinated and tightly regulated biological process. Exosome formation is initiated by the inward invagination of the plasma membrane, leading to the generation of early endosomes. During endosomal maturation, cytosolic cargoes are selectively sorted into the lumen through either ESCRT‐dependent mechanisms or ceramide‐mediated ESCRT‐independent pathways, ultimately resulting in the formation of MVBs containing ILVs (Ovcar and Kovacic 2024; Yu et al. 2023). Following maturation, MVBs are transported toward the plasma membrane under the regulation of Rab family GTPases. Subsequently, MVBs fuse with the plasma membrane through SNARE complex‐mediated membrane fusion, resulting in the extracellular release of ILVs as exosomes. In contrast, the biogenesis of microvesicles represents a more direct process that primarily depends on alterations in plasma membrane lipid asymmetry and cytoskeletal remodeling. Under the regulation of small GTPases such as ARF6 and RhoA, the plasma membrane undergoes outward budding and membrane fission, ultimately leading to microvesicle release (Mathieu et al. 2019; Yu 2021).

### 2.4 Overview of Extracellular Vesicle Sources

#### 2.4.1 Sources of Stem Cells: The Core Power of Regeneration

Owing to their low immunogenicity and potent paracrine regenerative capacity, stem cell‐derived EVs have emerged as one of the most promising platforms for cell‐free regenerative therapies. Among them, MSC‐derived EVs are the most extensively studied. EVs derived from adipose‐derived MSCs (ADSCs) exhibit strong therapeutic potential in skin rejuvenation, collagen regeneration, and soft tissue repair, partly through microRNA‐mediated attenuation of oxidative stress and cellular senescence (Luo et al. 2024; Wong et al. 2023; Zheng et al. 2023). Human umbilical cord‐derived MSC (hUC‐MSC)‐EVs possess pronounced immunomodulatory and pro‐angiogenic properties, making them attractive for the treatment of autoimmune disorders, acute kidney injury, and chronic wounds. In addition, bone marrow MSC‐derived EVs play important roles in bone and cartilage regeneration as well as osteoimmune regulation, whereas induced pluripotent stem cell (iPSC)‐derived EVs have recently gained increasing attention because of their potential in tissue rejuvenation, senescence reversal, and neuroprotection (Ding et al. 2024; Salehpour et al. 2024).

Collectively, the therapeutic functions of stem cell‐derived EVs are closely associated with the biological characteristics of their parental cells, highlighting the importance of source selection for specific anti‐aging and regenerative applications. Future studies should further establish standardized evaluation systems to optimize EV source selection, potency, and translational safety.

#### 2.4.2 Sources of Immune and Endothelial Cells: Modulators of the Microenvironment

EVs derived from immune cells and endothelial cells are key regulators of inflammatory microenvironments and vascular homeostasis. Macrophage‐derived EVs exhibit pronounced functional plasticity that reflects the polarization state of their parental cells. Pro‐inflammatory M1‐EVs enhance antimicrobial and immune defense responses, whereas anti‐inflammatory M2‐EVs, enriched in IL‐10 and TGF‐β, suppress tissue inflammation and promote tissue repair and regeneration (Lin et al. 2024; Ovcar and Kovacic 2024; Xia et al. 2020). In parallel, dendritic cell‐derived EVs express high levels of major histocompatibility complex (MHC) molecules and co‐stimulatory proteins, enabling their development as endogenous nanocarriers for tumor immunotherapy and vaccine delivery (Buzas 2023; Huang et al. 2024). Endothelial cell‐derived EVs also play dual and context‐dependent roles in vascular biology. Under physiological conditions, they contribute to vascular integrity and coagulation homeostasis; however, EVs released from senescent or damaged endothelial cells often carry inflammatory mediators and procoagulant factors that promote atherosclerosis and vascular calcification (Panda and Kubes 2023).

Together, these findings highlight the dynamic immunoregulatory and vascular‐modulating properties of immune‐ and endothelial‐derived EVs, suggesting their considerable potential as both therapeutic agents and disease biomarkers in aging‐associated inflammatory and cardiovascular disorders.

#### 2.4.3 Other Somatic, Organoid, and Special Vesicle Sources: Emerging Frontiers

Beyond the major EV sources described above, several emerging EV subtypes are attracting increasing attention because of their unique biological and therapeutic properties. Platelet‐derived EVs (PEVs), the most abundant vesicle population in circulating blood, are enriched in growth factors such as platelet‐derived growth factor (PDGF) and vascular endothelial growth factor (VEGF), enabling their broad application in hemostasis, wound healing, angiogenesis, and tissue regeneration. In parallel, organoid‐derived EVs have recently emerged as a promising research direction. Owing to the ability of three‐dimensional organoid systems to more accurately recapitulate the native tissue microenvironment, organoid‐derived EVs exhibit improved cargo fidelity and organ‐specific targeting capabilities compared with conventional two‐dimensional culture systems, particularly in liver, kidney, and brain organoid models (Zhou et al. 2024). In addition, mitochondria‐derived vesicles (MDVs) represent a distinct EV subtype generated independently of the canonical exosomal pathway through direct budding from mitochondrial membranes. MDVs participate in mitochondrial quality control by transporting damaged mitochondrial components and mitochondrial DNA (mtDNA), thereby influencing metabolic adaptation, innate immune signaling, and the senescence‐associated secretory phenotype (SASP). Furthermore, advances in bioengineering have accelerated the development of engineered EVs and plant‐derived vesicles as customizable platforms for drug delivery and precision nanomedicine (Herrmann et al. 2021; Jeppesen et al. 2019; Zhong et al. 2023; Ziegler and Tian 2023).

Collectively, the expanding diversity of EV sources is continuously broadening the functional landscape and translational potential of EV‐based therapeutics. Future studies should focus on clarifying source‐specific biological functions, improving scalable production strategies, and establishing standardized quality‐control systems for clinical application.

## 3 Mechanisms of EV‐Mediated Anti‐Aging Effects

EVs are nanoscale membrane‐bound structures secreted by nearly all cell types and serve as important mediators of intercellular communication. By transporting diverse bioactive cargoes, including proteins, lipids, mRNAs, microRNAs (miRNAs), long non‐coding RNAs (lncRNAs), and DNA, EVs regulate a wide range of physiological and pathological processes. In recent years, increasing evidence has demonstrated that EVs derived from young individuals or regenerative cell sources possess significant anti‐aging potential, with the ability to delay cellular senescence, restore tissue function, and improve organismal homeostasis.

The anti‐aging effects of EVs are mediated through multiple interconnected mechanisms. These include suppression of the SASP and remodeling of the immune microenvironment, regulation of mitochondrial homeostasis and metabolic reprogramming, modulation of DNA damage responses and epigenetic remodeling, activation of stem cell‐mediated regenerative signaling, restoration of autophagy–lysosomal function, and mediation of inter‐organ communication networks that contribute to systemic rejuvenation.

Collectively, these multifaceted mechanisms position EVs as promising systemic regulators rather than passive signaling carriers, highlighting their potential as next‐generation therapeutic platforms for aging and age‐related diseases. The major anti‐aging mechanisms mediated by EVs are discussed in the following sections.

### 3.1 Inhibition of SASP and Remodulation of the Immune Microenvironment

The SASP is a hallmark feature of senescent cells and is characterized by the persistent release of pro‐inflammatory cytokines, chemokines, and matrix‐degrading enzymes, which collectively drive chronic low‐grade inflammation, disrupt tissue homeostasis, and propagate senescence to neighboring cells (Cao et al. 2025; Wallis et al. 2021). Increasing evidence indicates that EVs can effectively suppress SASP activity by modulating inflammatory signaling pathways, remodeling immune responses, and facilitating the clearance of senescent cells (El‐Awady et al. 2022; Tesei et al. 2021; Zhu et al. 2025; Ziglari et al. 2025).

**FIGURE 2:**
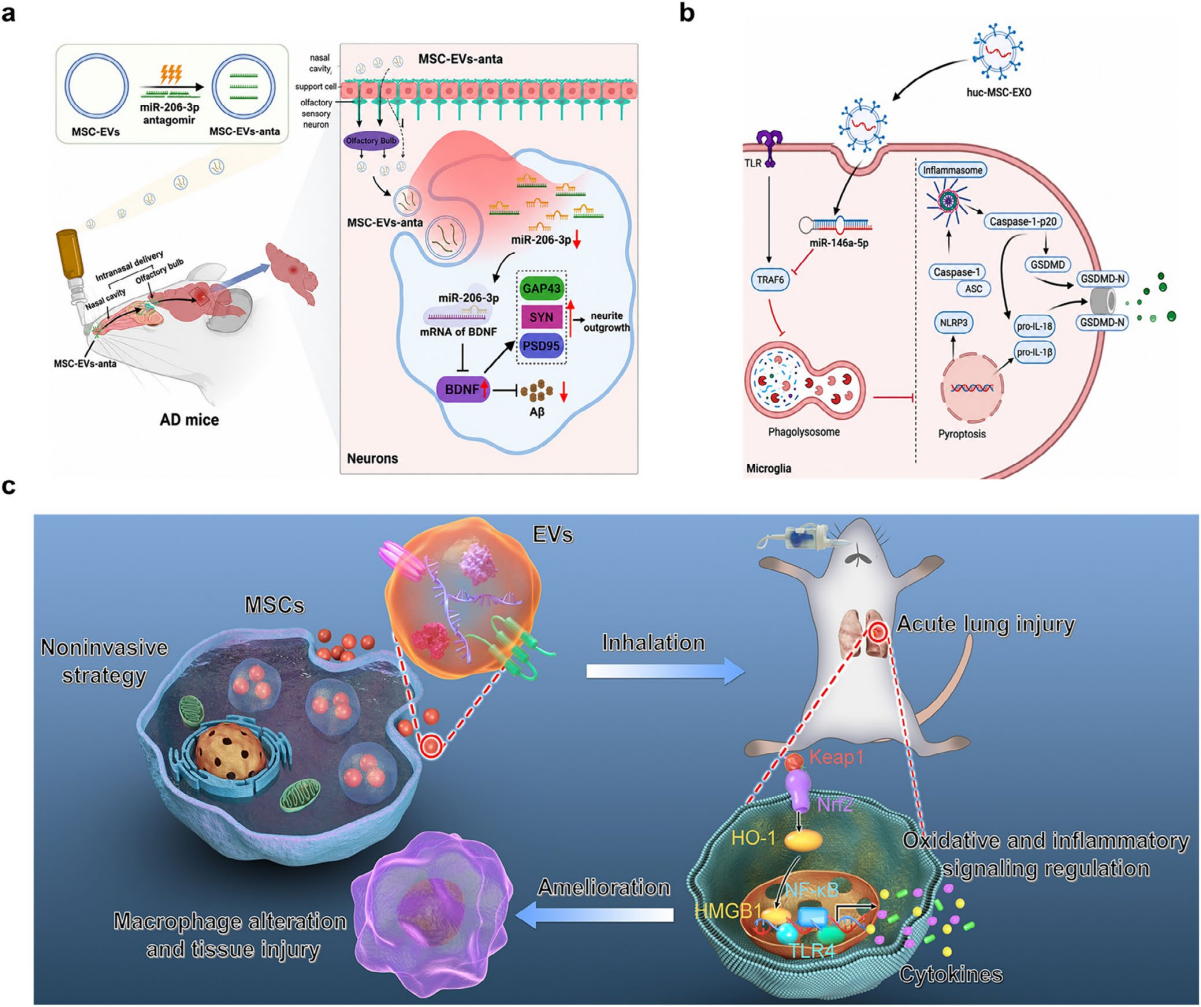
Extracellular vesicles delay aging by inhibiting the remodeling of the immune microenvironment and the accumulation of SASP. (a) MSC‐EVs deliver miR‐21‐5p to inhibit the SASP response in a mouse model of skin aging. Reproduced with permission (Peng, Liu, et al. 2024). Copyright 2024 Theranostics. (b) EVs regulate the miR‐146a‐5p‐mediated TRAF6/IRAK1 signaling pathway to alleviate neuroinflammation and improve cognitive function decline. Reproduced with permission (Hua et al. 2022). Copyright 2022 Springer Nature. (c) MSC‐EVs promote the polarization of M2‐type macrophages and inhibit inflammatory responses. Reproduced with permission (Zhao et al. 2022). Copyright 2022 Elsevier.

Among the most extensively studied mechanisms, MSC‐derived EVs significantly inhibit NF‐κB and p38 MAPK signaling in senescent cells, thereby reducing the secretion of inflammatory mediators such as IL‐6 and MCP‐1 and alleviating both local and systemic inflammation (Zhang, Zhang, et al. 2024; Zhang, Ji, Liu, et al. 2025). For example, human umbilical cord MSC‐EVs suppress SASP activation in aged skin by delivering miR‐21‐5p to regulate the PDCD4/NF‐κB signaling axis (Figure 2a) (Palama et al. 2023; Peng, Liu, et al. 2024). In the nervous system, EV‐mediated delivery of miR‐146a‐5p has been shown to attenuate neuroinflammation and improve cognitive impairment through modulation of TRAF6/IRAK1 signaling (Figure 2b) (Hua et al. 2022; Meng et al. 2024).

EVs also exert important immunoregulatory effects against immunosenescence, which is typically characterized by T‐cell exhaustion, macrophage polarization imbalance, and impaired NK cell activity (Dahlquist et al. 2024; Garabedian et al. 2025; Tao et al. 2025). MSC‐EVs promote macrophage polarization toward the anti‐inflammatory M2 phenotype, enhancing phagocytosis while suppressing excessive inflammatory responses (Figure 2c) (Kim et al. 2022; Zhao et al. 2022). In addition, adipose‐derived MSC‐EVs attenuate LPS‐induced cytokine storms through TSG‐6‐mediated anti‐inflammatory signaling (Jeong et al. 2025) whereas other EV populations help restore immune tolerance by regulating the Treg/Th17 balance (Hu, Li, et al. 2024).

Notably, certain EVs possess intrinsic senolytic potential through the delivery of specific microRNAs and regulatory factors. For instance, EV‐associated members of the miR17 ~ 92 cluster target senescence‐related genes such as BCL2L11 and CDKN1A, thereby facilitating the selective elimination of senescent cells and indirectly suppressing SASP propagation (Zhang, Zhang, et al. 2024; Zhang, Nunes, Lee, et al. 2025). Engineered EV‐mediated delivery of miR‐34a inhibitors has similarly been shown to reduce the accumulation of p16^INK4a^ positive senescent cells in the lung (Gray and Johnson 1976). Collectively, EVs function as multifaceted regulators of inflammatory and immune aging by suppressing SASP signaling, restoring immune homeostasis, and limiting senescence propagation. Future studies should further optimize EV engineering strategies to improve tissue‐specific immunomodulation and enhance the long‐term therapeutic efficacy of EV‐based senotherapeutics (Gorgun et al. 2022; Kumar et al. 2025; Morente‐Lopez et al. 2023; Morente‐Lopez et al. 2022; Pulver et al. 2025; Zhang, He, Xiao, et al. 2025).

### 3.2 Mitochondrial Regeneration and Metabolic Reprogramming

**FIGURE 3:**
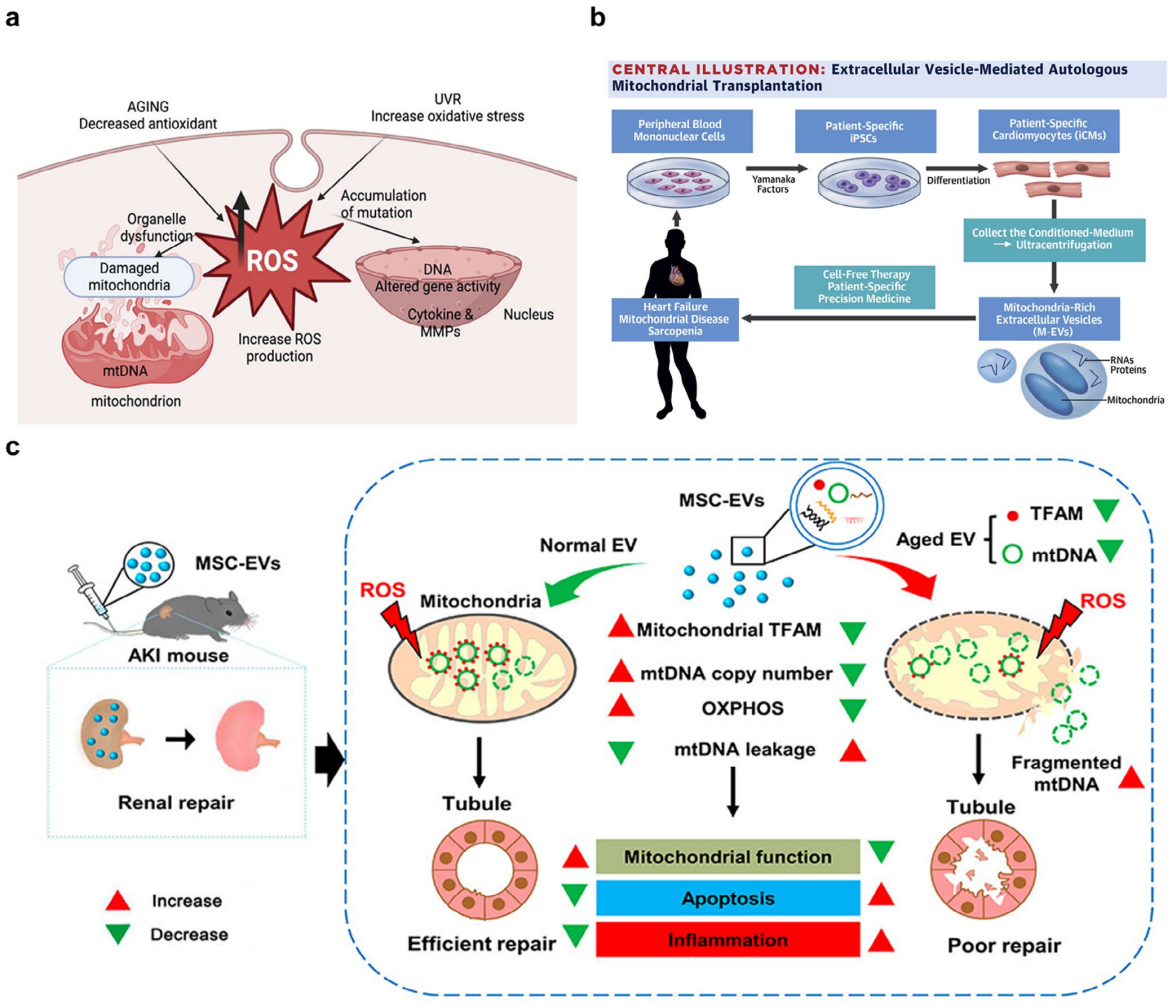
Extracellular vesicles reprogram the homeostasis and metabolism of mitochondria. (a) The core feature of mitochondrial dysfunction. Reproduced with permission (Somasundaram et al. 2024). Copyright 2024 Somasundaram. (b, c) EVs regulate the expression of mitochondrial‐related genes by transferring functional mitochondrial components, thereby restoring the cellular energy metabolism homeostasis. Reproduced with permission (Ikeda et al. 2021). Copyright 2021 American College of Cardiology Foundation. Reproduced with permission (Zhao et al. 2021). Copyright 2020 American Chemical Society.

Mitochondrial dysfunction is a central hallmark of aging and is characterized by impaired ATP production, excessive reactive oxygen species (ROS) accumulation, loss of mitochondrial membrane potential, and dysregulated mitochondrial dynamics (Figure 3a) (Berry and Kaeberlein 2021; DeBalsi et al. 2017; Lejri et al. 2024; Panfilova et al. 2025; Shou et al. 2025; Sinha et al. 2025; Somasundaram et al. 2024). Given the pivotal role of mitochondria in cellular metabolism and stress responses, restoration of mitochondrial homeostasis has emerged as a major therapeutic target in anti‐aging interventions. Increasing evidence suggests that EVs can improve cellular energy metabolism by transferring functional mitochondrial components and regulating mitochondria‐associated signaling pathways. MSC‐derived EVs have been reported to contain intact mitochondrial DNA (mtDNA), electron transport chain proteins, and antioxidant enzymes, which can be internalized by recipient cells and incorporated into existing mitochondrial networks, thereby improving respiratory chain activity and reducing oxidative stress (Figure 3b,c) (Kim et al. 2023; Lu, Zhang, et al. 2022; Peruzzotti‐Jametti et al. 2021; Zhao et al. 2021). In addition, several studies have demonstrated that MSC‐EVs are capable of transferring functional mitochondria to injured cells, including damaged alveolar epithelial cells and ischemic cardiomyocytes, resulting in increased ATP production, reduced ROS accumulation, and improved cellular survival (Dutra Silva et al. 2021; Jiang et al. 2016; Liang et al. 2023; Tolomeo et al. 2024). These findings highlight the important role of EVs in mitochondrial quality control and metabolic rejuvenation. Future research should further clarify the mechanisms governing mitochondrial cargo loading, intercellular mitochondrial transfer efficiency, and long‐term metabolic safety to facilitate the development of mitochondria‐targeted EV therapeutics for aging and age‐related diseases.

Beyond direct mitochondrial restoration, EVs also participate in metabolic reprogramming by regulating the balance between glycolysis and oxidative phosphorylation. Young plasma‐derived EVs, for example, are enriched in microRNAs such as miR‐126 and miR‐221, which enhance glucose uptake and mitochondrial biogenesis through activation of the PI3K/Akt signaling pathway (Katayama et al. 2019; Li, Xu, et al. 2024; Wozniak et al. 2025). Similarly, exosomes derived from human umbilical cord mesenchymal stem cells (hUCMSC‐Exo) promote mitochondrial biogenesis and improve skeletal muscle function in aged mice by activating the AMPK/PGC‐1α pathway (Song, Liu, et al. 2023).

In addition, attenuation of oxidative stress represents another important mechanism by which EVs counteract aging‐associated metabolic dysfunction. EVs can deliver antioxidant molecules and redox‐regulatory microRNAs to recipient cells. For instance, dental pulp stem cell‐derived EVs reduce oxidative stress and delay endothelial senescence through miR‐200c‐mediated regulation of the Keap1/Nrf2 signaling axis (Luo et al. 2025). Collectively, EVs contribute to mitochondrial repair and metabolic rejuvenation through coordinated regulation of mitochondrial biogenesis, energy metabolism, and ROS clearance. These multifunctional metabolic effects further support the potential of EVs as promising therapeutic platforms for reversing aging‐associated energy imbalance and mitochondrial decline.

### 3.3 DNA Damage Repair and Epigenetic Remodeling

Genomic instability is a core hallmark of aging and is characterized by telomere attrition, accumulation of DNA double‐strand breaks (DSBs), and progressive decline in DNA repair capacity (Bolzan 2021; Delabaere et al. 2017; Medoro et al. 2024). Increasing evidence suggests that EVs can enhance genomic maintenance and cellular resilience by delivering bioactive molecules involved in DNA damage response (DDR) pathways (Han et al. 2025; Ribas‐Maynou et al. 2025).

MSC‐derived EVs are enriched in multiple DDR‐associated proteins, including ATM, BRCA1, RAD51, and PARP1, which contribute to accelerated clearance of γH2AX foci and improved DNA repair efficiency in recipient cells. In particular, bone marrow MSC‐EVs have been shown to alleviate ionizing radiation‐induced DNA damage and preserve genomic stability through activation of the ATM–Chk2–p53 signaling pathway (Firoozi et al. 2020; Han et al. 2025; Hu et al. 2023; Mai et al. 2025). Beyond DNA repair, EVs also participate extensively in epigenetic remodeling through the transfer of regulatory non‐coding RNAs and epigenetic enzymes. For example, the miR‐302 family suppresses DNMT1 expression and reduces global DNA methylation levels, thereby promoting a more rejuvenated epigenetic state. In parallel, the long non‐coding RNA MALAT1 regulates H3K27me3 modification through interaction with EZH2, influencing the transcriptional silencing of senescence‐associated genes. In addition, EV‐mediated delivery of SIRT1 contributes to telomere maintenance and suppression of p16^INK4a^ expression (Chiba et al. 2023; Peng, Zhao, et al. 2024; Zhang, Zhang, Sui, et al. 2025; Zhang, Nunes, Lee, et al. 2025).

Collectively, these findings indicate that EVs can modulate both genomic stability and epigenetic plasticity, highlighting their potential to reverse aging‐associated molecular damage at the transcriptional and chromatin levels. Future studies should further investigate the long‐term genomic safety and epigenetic specificity of EV‐based interventions to optimize their translational application in anti‐aging therapeutics.

### 3.4 Targeting Tissue Regenerative Pathways

Aging‐associated decline in tissue regenerative capacity is largely attributed to adult stem cell exhaustion and dysregulation of developmental signaling pathways. Increasing evidence suggests that EVs can restore regenerative potential by reactivating quiescent stem cells and modulating key pathways involved in tissue repair and cellular renewal. Among these pathways, Wnt/β‐catenin signaling plays a central role in stem cell self‐renulation and regeneration. MSC‐derived EVs have been reported to carry Wnt‐related ligands, including Wnt3a, Wnt5a, and LRP6, which promote β‐catenin nuclear translocation and enhance neural stem cell proliferation (Cox et al. 2021; Leedham 2020; O'Brien et al. 2017). In osteoarthritis models, synovial MSC‐derived EVs stimulate chondroprogenitor differentiation through Wnt pathway activation, thereby slowing cartilage degeneration and joint dysfunction (Ye et al. 2022).

Notch signaling is another key regulator of stem cell fate determination and tissue regeneration. Increasing evidence suggests that stem cell‐derived EVs can modulate regenerative signaling pathways and promote neural regeneration and neurogenesis in aging‐associated conditions (Bonetto and Grilli 2023). In addition, EVs have been reported to activate satellite cells and promote skeletal muscle regeneration, further supporting the therapeutic potential of EV‐mediated regenerative signaling in age‐related tissue dysfunction (Porcu et al. 2024).

EVs also regulate other conserved regenerative pathways, including Hedgehog and YAP/TAZ signaling. Adipose‐derived MSC‐EVs containing Sonic hedgehog (Shh) protein activate Gli1‐dependent transcription to promote hair follicle stem cell proliferation and improve alopecia‐related phenotypes. In addition, EVs can modulate YAP/TAZ mechanotransduction signaling through integrin‐associated pathways, thereby enhancing stem cell mechanosensitivity and regenerative responsiveness.

EVs function as potent “signal‐transmitting” platforms that reactivate developmental and regenerative programs through the delivery of key ligands and signaling regulators. Future studies should further investigate how engineered EVs can selectively modulate regenerative pathways in a tissue‐specific manner to improve the precision and durability of anti‐aging regenerative therapies.

### 3.5 Restoration Autophagy‐Lysosomal Pathway for Proteostasis

Age‐associated decline in autophagic and lysosomal function contributes to the accumulation of misfolded proteins and damaged organelles, thereby promoting neurodegenerative disorders and metabolic dysfunction (Cassidy et al. 2020; Guo et al. 2018; Kaushik et al. 2021). Increasing evidence indicates that EVs can restore proteostasis by modulating key regulators of autophagy and intracellular degradation pathways.

MSC‐derived EVs have been shown to enhance autophagic activity through upregulation of ULK1, Beclin‐1, and LC3‐II, thereby promoting autophagosome formation. Mechanistically, EV‐associated miR‐214‐3p suppresses PTEN expression and subsequently activates autophagy through the Akt/mTORC1 signaling axis (Hou et al. 2022; Sang et al. 2020). In neurodegenerative disease models, induced pluripotent stem cell‐derived EVs (iPSC‐EVs) significantly reduce Aβ plaque accumulation and phosphorylated Tau deposition, partly through Transcription Factor EB (TFEB)‐mediated enhancement of lysosomal biogenesis and clearance capacity (Rao et al. 2025; Zheng et al. 2025).

In addition to autophagy regulation, EVs can also transport molecular chaperones and components of the ubiquitin–proteasome system that facilitate the degradation of abnormal or aggregated proteins (Albakova et al. 2021; Hu, Liu, et al. 2024). For example, young liver‐derived EVs enriched in the co‐chaperone Bag3 promote selective clearance of protein aggregates and attenuate the progression of liver fibrosis (Chen et al. 2018; Chen, Ma, et al. 2024). EVs restore intracellular proteostasis by coordinately activating autophagy–lysosomal pathways and protein quality‐control systems, thereby alleviating aging‐associated proteotoxic stress. Future studies should focus on improving the specificity and persistence of EV‐mediated proteostasis regulation to enhance therapeutic efficacy in neurodegenerative and metabolic diseases (Van den Broek et al. 2022).

### 3.6 Cross‐Organ Communication and Systemic Rejuvenation

Recent studies increasingly suggest that EVs can exert systemic anti‐aging effects through blood circulation‐mediated cross‐organ communication. Evidence from heterochronic parabiosis experiments has demonstrated that circulating factors derived from young organisms can reverse aging phenotypes in multiple tissues of aged animals, with EVs now recognized as key mediators of these rejuvenating effects. Notably, young plasma‐derived EVs have been shown to cross the blood–brain barrier, enhance hippocampal neurogenesis, and improve cognitive function in aged mice (Fitz et al. 2023). Systemically administered EVs also exhibit broad tissue distribution and inter‐organ regulatory capacity. For example, following cardiac administration of MSC‐derived EVs, labeled vesicles were detected in the brain, liver, and kidneys, accompanied by improved metabolic activity and reduced systemic inflammation. Increasing evidence further indicates that organ‐specific EV targeting is partially mediated by surface molecules such as integrins and receptor‐associated ligands (Hoshino et al. 2015). For example, liver‐tropic EVs express high levels of ASGPR1 ligands, whereas brain‐tropic EVs have a high concentration of transferrin receptors (Wiklander et al. 2015). Engineered EVs can also be designed as “multi‐organ delivery systems” to enable synergistic interventions. Preclinical studies have confirmed that the intravenous infusion of EVs from young donors can extend the lifespan of progeroid mice by around 20%, as well as improving their exercise capacity, hair density, and wound healing speed. Preliminary human trials also demonstrate that infusion of MSC‐EVs improves inflammatory markers and immune function in elderly volunteers (Mahindran et al. 2023). Preliminary clinical observations similarly suggest that MSC‐EV administration may improve inflammatory profiles and immune function in elderly individuals.

These findings support the concept that EVs function as systemic signaling mediators capable of coordinating rejuvenation across distant organs and tissues. Future studies should further elucidate the mechanisms governing EV biodistribution, organ tropism, and long‐term systemic safety to facilitate the development of EV‐based systemic anti‐aging therapies.

### 3.7 Limitations and Prospects of Existing Mechanism Research

Collectively, the six major anti‐aging mechanisms discussed above including immune microenvironment remodeling, mitochondrial restoration and metabolic reprogramming, DNA damage repair and epigenetic modulation, activation of regenerative signaling pathways, recovery of autophagy–lysosomal function, and cross‐organ communication‐mediated rejuvenation highlight the multifaceted therapeutic potential of EVs in delaying aging and restoring tissue homeostasis. However, despite these encouraging findings, several important technical bottlenecks and methodological limitations continue to restrict mechanistic understanding and translational development.

One of the most significant challenges is the pronounced heterogeneity of EV sources and preparation procedures. Variations in parental cell types, culture conditions, isolation techniques, and characterization standards across laboratories substantially affect EVs' composition and biological activity, thereby limiting reproducibility and direct comparison between studies. Consequently, even when similar signaling pathways are investigated, the identified core effector molecules often differ considerably. In addition, most current studies rely heavily on short‐term in vitro co‐culture systems or acute injury animal models, which are insufficient to recapitulate the chronic, progressive, and systemic nature of biological aging. Although transient pathway activation and phenotypic improvements are frequently observed, the long‐term persistence, tolerance, and compensatory responses associated with chronic EVs' administration remain poorly understood.

Mechanistic validation strategies also remain limited. Most studies infer EVs function through overexpression or knockdown of individual nucleic acids or proteins; however, EVs simultaneously carry hundreds of bioactive molecules that may function synergistically or redundantly. Therefore, attributing a biological effect solely to a single EVs‐associated molecule often lacks rigorous causal evidence. Similar limitations exist in studies of EVs‐mediated cross‐organ communication. Current fluorescence‐ and bioluminescence‐based tracing methods cannot reliably distinguish intact vesicle delivery from signals generated by degraded labeling components. Moreover, following systemic administration, the majority of EVs are rapidly sequestered by the liver and spleen, while only a small fraction reaches distal target tissues. As a result, it remains unclear whether observed systemic rejuvenation effects arise from direct EV delivery to target organs or from secondary signaling cascades initiated by scavenging organs after EV uptake.

Another critical issue is that most mechanistic studies are performed in young and otherwise healthy animal models. In contrast, aged organisms exhibit chronic inflammation, vascular dysfunction, immune dysregulation, and impaired phagocytic activity, all of which can substantially alter EVs biodistribution, clearance kinetics, and cellular uptake efficiency. Consequently, mechanistic conclusions derived from young models may not accurately reflect EVs' behavior in aged physiological environments.

Future studies should therefore prioritize systematic methodological optimization. Establishing standardized reference materials and unified characterization criteria for EVs preparation will be essential for improving cross‐study reproducibility. In parallel, the incorporation of chronic aging models and long‐term administration paradigms will help clarify the temporal and dose‐dependent effects of EV‐based interventions. Advances in single‐vesicle tracking technologies, high‐resolution functional imaging, and genetic tools capable of distinguishing direct from indirect EV‐mediated effects will also be critical for mechanistic dissection.

Only by overcoming these methodological limitations can EVs research truly progress from descriptive observations toward rigorous causal validation, thereby providing a more reliable mechanistic foundation for the development of next‐generation anti‐aging therapeutics and translational nanomedicine platforms.

## 4 The Anti‐Aging Application of EVs

Aging is fundamentally characterized by the progressive accumulation of senescent cells and the persistent activation of the SASP, which together drive chronic low‐grade inflammation and systemic microenvironmental dysfunction (Boulestreau et al. 2021; Lopez‐Otin et al. 2023). As important mediators of intercellular communication, EVs have emerged as critical regulators of healthy aging, with growing evidence supporting their ability to remodel the aging microenvironment, suppress inflammatory signaling, and alleviate tissue degeneration (Rudnitsky et al. 2024).

Recent studies have demonstrated that EVs derived from young mesenchymal stem cells can attenuate aging‐associated inflammation by delivering anti‐aging microRNAs, long non‐coding RNAs, and regulatory proteins to senescent recipient cells, thereby suppressing pro‐inflammatory pathways such as NF‐κB signaling (Ala 2023; Rather et al. 2023). For example, human umbilical cord mesenchymal stem cell‐derived EVs have been shown to reduce inflammatory factor release in skeletal muscle and adipose tissue, leading to improved grip strength and motor coordination in aged mouse models (Rudnitsky et al. 2024; Zhang, Li, and Chen 2024). Beyond their natural biological functions, engineered EVs are also being developed as targeted senotherapeutic delivery platforms. By loading EVs with senolytic agents such as the FOXO4‐DRI peptide, researchers have achieved selective induction of apoptosis in senescent cells, offering a promising strategy for precise systemic anti‐aging intervention (Rudnitsky et al. 2024; Yuan et al. 2025). Collectively, these findings highlight the potential of EVs not only as endogenous regulators of the aging microenvironment but also as programmable therapeutic platforms for targeted senescence modulation and precision geromedicine. Recent studies have revealed that EVs derived from stem cells, plasma, immune cells, and other biological sources exert protective effects in neurodegenerative diseases, pulmonary injury, cardiovascular dysfunction, and age‐related tissue degeneration through distinct molecular mechanisms. Representative applications of EVs in aging‐associated diseases are summarized in Table (Table 1).

**TABLE 1:** Therapeutic applications, key cargoes, and mechanisms of EVs in aging‐associated diseases.

Disease	EV source	Key cargo	Mechanism	References
Neuroinflammation	Plasma extracellular vesicle	Plasma EV LINE‐1 RNA	Inhibition cGAS‐STING pathway	(Yu et al. 2026)
Parkinson's disease	Umbilical cord blood‐derived exosomes	Angiogenic proteins	Inhibition of hyperphosphorylation of MAPK p38 and ERK 1/2 signaling pathways	(Ye et al. 2024)
Lung injury	Human serum‐derived EVs	Human serum‐derived EVs	Inhibit the AKT pathway	(Zhou et al. 2021)
Chronic obstructive pulmonary disease	Human umbilical cord mesenchymal stem cell‐derived EVs	Micro‐RNA content of MSC‐derived exosome	Reduced the levels of NF‐κB subunit p65	(Ridzuan et al. 2021)
Cardiac dysfunction	Umbilical cord mesenchymal stem cell‐derived EVs	IncRNA MALAT1	Exosome/lncRNA MALAT1/NF‐κB/TNF‐α pathway	(Zhu et al. 2019)
Aging in mammals	Mouse plasma EVs	Extracellular nicotinamide phosphoribosyltransferase	NAD Biosynthesis	(Yoshida et al. 2019)
Endothelial cell senescence	Mesenchymal stem cell‐derived exosomes	MiR‐146a in small EVs	Downregulating Src signal pathway	(Xiao et al. 2021)
Brain aging	Mesenchymal stem cell‐derived exosomes	Mesenchymal stem cell‐derived exosomes	Activating the SIRT1 signaling pathway	(Zhang et al. 2023)

### 4.1 Application of EVs in Neurodegenerative Diseases

In the field of neurodegenerative diseases, EVs have emerged as promising diagnostic and therapeutic platforms because of their ability to cross the blood–brain barrier (BBB) and transport central nervous system (CNS)‐derived molecular signals into the peripheral circulation. This unique property enables minimally invasive monitoring of pathological changes within the brain microenvironment (D'Acunzo et al. 2023). Recent multicenter studies have demonstrated that immunoaffinity enrichment of neuron‐derived EVs (NDEs) from plasma provides highly sensitive detection of early neurodegenerative alterations. In Alzheimer's disease (AD), analysis of EV‐associated biomarkers such as phosphorylated tau (p‐tau217) and Aβ42/40 ratios has shown strong concordance with amyloid PET imaging and can identify pathological changes years before clinical symptom onset (Crane et al. 2024; Kwon et al. 2024). Similarly, combined detection of α‐synuclein oligomers and mitochondrial proteins within plasma EVs has shown diagnostic value for distinguishing Parkinson's disease (PD) from atypical parkinsonian syndromes, highlighting the potential of EVs for precision patient stratification (Fiorenzato et al. 2024).

Beyond diagnosis, mesenchymal stem cell‐derived EVs are increasingly recognized as promising cell‐free therapeutics for neurodegenerative disorders because of their intrinsic neuroprotective and immunomodulatory properties. Rather than directly replacing damaged cells, MSC‐EVs primarily function through modulation of neuroinflammatory networks and restoration of neuronal homeostasis. Mechanistically, EV‐delivered microRNAs suppress excessive microglial activation and inhibit the transition from the neuroprotective M2 phenotype toward the neurotoxic M1 state, thereby alleviating chronic neuroinflammation (Dietl et al. 2024). In addition, intranasal administration has emerged as an attractive delivery strategy because it enables EVs to bypass the BBB and directly access the brain parenchyma, leading to enhanced synaptic plasticity and reduced neuronal apoptosis in AD models. Compared with conventional stem cell transplantation, EV‐based therapies offer improved biosafety, lower immunogenicity, and greater translational feasibility.

Recent advances in bioengineering have further expanded the therapeutic potential of EVs for CNS diseases. Surface modification strategies using rabies virus glycoprotein (RVG29), Lamp2b fusion proteins, or receptor‐targeting peptides significantly enhance EV brain‐targeting efficiency by promoting selective interaction with neuronal receptors (Detappe et al. 2023; Hilal et al. 2024; Zlotnick et al. 2024). Furthermore, engineered EVs have been successfully loaded with siRNAs targeting BACE1 or CRISPR/Cas‐based gene editing systems using electroporation and bioorthogonal conjugation approaches (Zhang, Zhang, Zhou, et al. 2025; Werninghaus et al. 2023). In vivo studies demonstrate that these engineered EVs can selectively accumulate in the cortex and hippocampus following systemic administration, reducing amyloid burden while minimizing off‐target toxicity in peripheral organs (Yu, Wang, et al. 2024). Collectively, these findings highlight the dual value of EVs as both liquid biopsy biomarkers and precision therapeutic platforms for neurodegenerative diseases. Future studies should focus on improving BBB targeting efficiency, cargo loading stability, and large‐scale manufacturing standardization to accelerate the clinical translation of EV‐based precision neuromedicine for disorders such as AD, Parkinson's disease, Huntington's disease, and amyotrophic lateral sclerosis (ALS) (Byun et al. 2023).

### 4.2 Therapeutic Applications of Extracellular Vesicles in Lung Aging‐Related Diseases and Breakthroughs in Inhalation Delivery

Recent advances in EV‐based therapies for age‐related lung diseases have primarily focused on reversing two major pathological features of pulmonary aging: exhaustion of type II alveolar epithelial cell (AECII) stemness and persistent fibroblast senescence. Increasing evidence suggests that EVs can interrupt the vicious cycle of fibrosis, inflammation, and tissue degeneration through coordinated regulation of epithelial repair, metabolic homeostasis, and immune remodeling.

MSC‐derived engineered EVs have been shown to deliver anti‐fibrotic microRNAs that suppress key profibrotic pathways, including TGF‐β and Wnt/β‐catenin signaling. In aged models of bleomycin‐induced pulmonary fibrosis and silicosis, these EVs restored E‐cadherin expression while reducing α‐SMA levels, thereby inhibiting epithelial–mesenchymal transition (EMT) and attenuating fibrotic progression (Neary et al. 2024; Suades et al. 2024). In parallel, EV‐mediated delivery of anti‐aging regulators such as SIRT1 has been reported to alleviate senescence‐associated arrest in alveolar epithelial cells by suppressing p53/p21 signaling and reducing excessive DNA damage responses, ultimately promoting regeneration of aged alveolar progenitor cells and maintaining alveolar integrity (Mi and Graham 2023; Schaaf et al. 2024). Beyond anti‐fibrotic activity, EVs also play important roles in pulmonary metabolic reprogramming and immune regulation. Recent studies demonstrated that iPSC‐derived MSC‐EVs can transfer functional mitochondria to injured alveolar epithelial cells and macrophages through membrane fusion or tunneling nanotube‐associated mechanisms, thereby increasing ATP production, reducing ROS, and suppressing NLRP3 inflammasome activation (Curvello et al. 2024). In addition, engineered EVs carrying anti‐inflammatory cargos or specific siRNAs can promote macrophage polarization toward reparative phenotypes while reducing IL‐1β and TNF‐α secretion, thus alleviating chronic inflammaging‐associated lung injury (Stanczak and Pearce 2024; Zhao et al. 2023).

Collectively, these findings highlight the multifunctional capacity of EVs to simultaneously target fibrosis, mitochondrial dysfunction, epithelial senescence, and chronic inflammation in the aging lung. Future studies should focus on improving lung‐specific delivery efficiency, optimizing long‐term biosafety, and integrating EV engineering with precision pulmonary medicine to facilitate clinical translation for chronic age‐related respiratory diseases.

### 4.3 Application of EVs in Myocardial Infarction Repair

**FIGURE 4:**
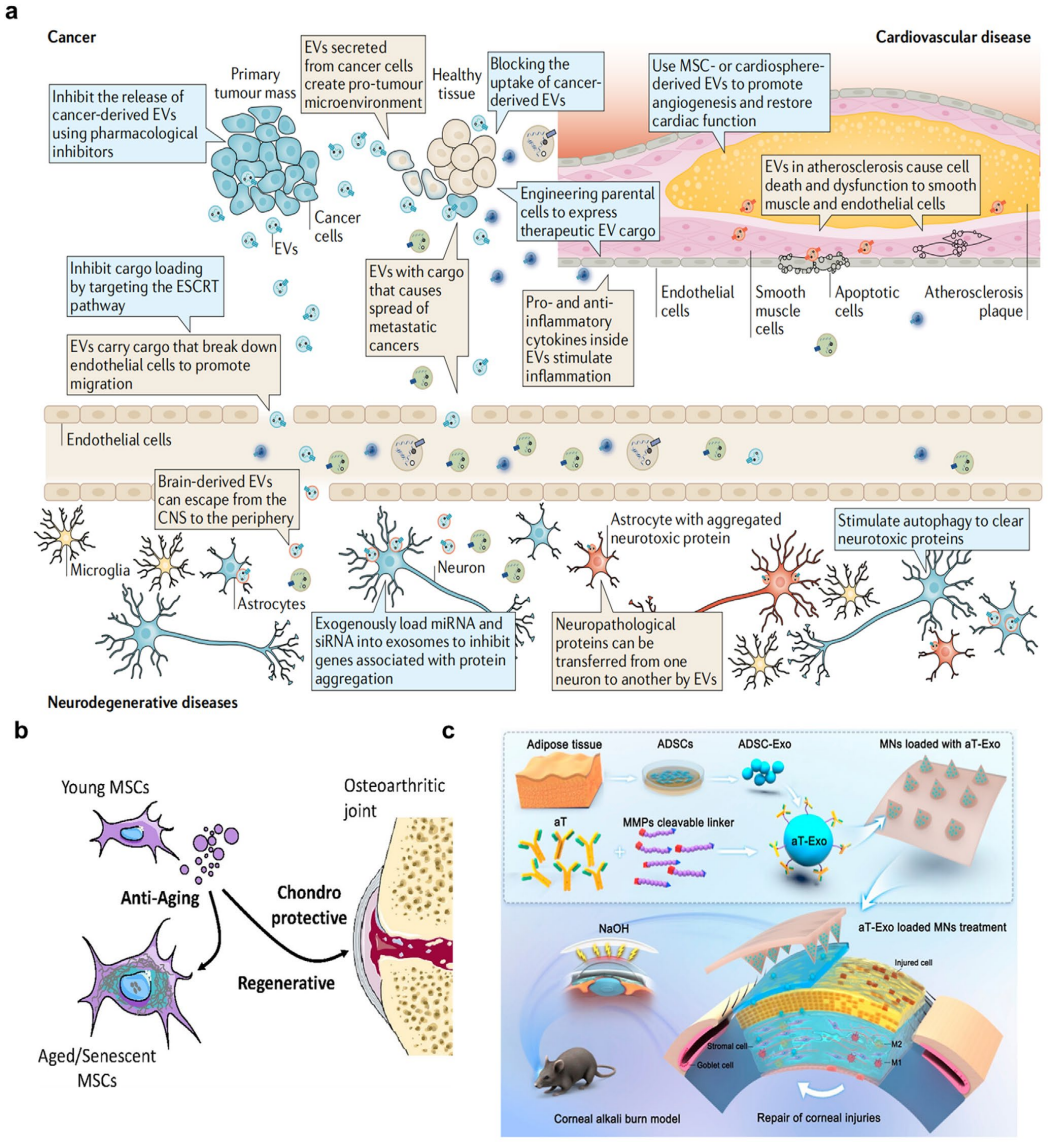
The Anti‐aging Application of EVs. (a) The role of EVs in cancer, neurodegenerative diseases, and cardiovascular diseases. Reproduced with permission (Cheng and Hill 2022). Copyright 2022 Springer Nature. (b) The potential therapy of treating osteoarthritis with EVs derived from MSC‐EVs. Reproduced with permission (Boulestreau et al. 2021). Copyright 2021 Elsevier. (c) Engineering mesenchymal interstitial cell‐loaded exosome microneedles to improve corneal healing after chemical injury. Reproduced with permission (Yu, Zhao, et al. 2024) Copyright 2024 American Chemical Society.

The maintenance of cardiovascular homeostasis relies heavily on intercellular vesicle‐mediated communication, and EVs have demonstrated significant therapeutic potential in myocardial repair, angiogenesis, and vascular rejuvenation (Figure 4a). In myocardial infarction models, EVs enriched with pro‐angiogenic factors and cardioprotective microRNAs, including miR‐21 and miR‐126, activate survival signaling pathways in injured myocardium, thereby reducing cardiomyocyte apoptosis, promoting microvascular regeneration, and improving cardiac function (Ala 2023; Boulestreau et al. 2021). These effects contribute to reduced infarct size and enhanced cardiac ejection fraction, highlighting the regenerative potential of EV‐based therapies in ischemic heart disease.

EVs also play important roles in vascular aging and atherosclerosis. Endothelial‐derived EVs have been shown to suppress the phenotypic transition of vascular smooth muscle cells, thereby attenuating vascular calcification, stiffness, and age‐associated vascular remodeling. In parallel, circulating EV signatures are increasingly being explored as minimally invasive biomarkers for early cardiovascular lesions and disease progression (Ala 2023; Phan et al. 2022; Yuan et al. 2025).

Beyond diagnosis, engineered EVs loaded with anti‐inflammatory cargos can selectively target macrophages within atherosclerotic plaques and suppress local inflammatory responses, supporting the emerging concept of EV‐based cardiovascular theranostics that integrate both diagnostic and therapeutic functions.

Collectively, these findings underscore the multifaceted role of EVs in cardiovascular regeneration, vascular homeostasis, and precision theranostics. Future research should focus on improving cardiac‐targeting efficiency, optimizing large‐scale manufacturing, and validating long‐term biosafety to accelerate the clinical translation of EV‐based therapies for age‐related cardiovascular diseases.

### 4.4 EVs in Treating Osteoarthritis and Musculoskeletal Diseases

In osteoarthritis (OA), recent advances in EV‐based therapy have focused on overcoming the limited targeting efficiency and reparative capacity of native EVs through bioengineering strategies and organelle‐based therapeutic approaches (Figure 4b) (Bhimavarapu et al. 2024; De Sota et al. 2024; Gonzalez‐Rodriguez et al. 2025; Luo et al. 2024). One representative strategy involves the development of hybrid nanovesicles that combine the biological activity of EVs with the targeting capability of synthetic nanomaterials. For example, a hybrid vesicle system constructed by fusing TGF‐β1‐overexpressing EVs with Col2a1 antibody‐modified liposomes demonstrated enhanced penetration into deep cartilage layers in a rat destabilization of the medial meniscus (DMM) model, resulting in substantial restoration of cartilage thickness and marked reduction of OA severity scores (Chen, Tian, et al. 2025; Gonzalez‐Rodriguez et al. 2025; Kim et al. 2024).

In addition to molecular payload delivery, mechanisms based on mitochondrial transfer have also been validated. A 2025 study using advanced tracking technology revealed that mitochondria‐rich extracellular vesicles (Mito‐EVs) derived from synovial mesenchymal stem cells could transport functional mitochondria into damaged chondrocytes, thereby inhibiting cellular senescence and delaying osteoarthritis progression (Li, Lu, et al. 2025). This process significantly inhibits chondrocyte senescence and apoptosis by restoring ATP synthesis and attenuating oxidative stress via modulation of energy metabolism (Belhadj et al. 2023; He et al. 2025). In addition, physical stimulation approaches can further enhance EV production efficiency while enriching anti‐inflammatory microRNAs, enabling more effective inhibition of cartilage matrix‐degrading enzymes (Huan et al. 2024; Wang, Zhao, et al. 2024).

In rheumatoid arthritis (RA) and synovial inflammation, EV research has increasingly shifted from simple cytokine suppression toward immune‐metabolic reprogramming and regulation of pathogenic cell death pathways (Beg et al. 2024; Yuan, Jiang, et al. 2024). Recent studies demonstrated that engineered macrophage‐derived EVs loaded with copper sulfide nanoparticles can selectively induce cuproptosis in pathogenic T cells while simultaneously promoting regulatory T‐cell differentiation through TGF‐β signaling, thereby restoring local immune tolerance (Wu, Su, et al. 2025). This successfully re‐establishes local immune tolerance. In terms of metabolic regulation, using drug‐loaded EVs to polarize macrophages has become the dominant strategy (Yuan, Yang, et al. 2024). A 2024 study described Icariin‐loaded adipose‐derived stem cell exosomes that promote the repolarisation of synovial M1 macrophages toward an anti‐inflammatory M2 phenotype by inhibiting glycolysis. This significantly reduces IL‐1β and TNF‐α concentrations in synovial fluid (Yan et al. 2024). Other engineered EVs with high PD‐L1 expression have been shown to inhibit synovial fibroblast hyperproliferation and pannus formation, further highlighting the potential of EV‐based cell‐free immunotherapy (Silva et al. 2024).

To improve delivery efficiency and intra‐articular retention, multiple biomaterial‐assisted EV delivery systems have also been developed. Microneedle‐based EV patches and injectable hydrogel scaffolds can prolong EV retention within joint tissues, enhance ROS scavenging, and provide mechanical support for cartilage repair through synergistic activation of regenerative signaling pathways (Bui et al. 2024; LeBar et al. 2024; Li, Lu, et al. 2024; Wang, Cheng, and Su 2024; Wang, Guan, et al. 2024; Xiong et al. 2024). Moreover, surface engineering strategies using cartilage affinity peptides or folate ligands significantly improve active targeting toward damaged cartilage and inflammatory macrophages, thereby enhancing therapeutic precision and biosafety (Chen, Ma, et al. 2024; Chen, Chen, et al. 2024; Douez et al. 2024). Collectively, these advances demonstrate that EV‐based therapies for OA and RA are evolving from passive biological interventions toward highly engineered precision nanomedicine platforms integrating targeting capability, metabolic regulation, immune remodeling, and tissue regeneration. Future studies should further optimize long‐term retention, large‐scale manufacturing, and clinical‐grade quality control to accelerate translation into regenerative therapies for musculoskeletal aging diseases.

### 4.5 Use of EVs in Skin Aging and Visual Degenerative Diseases

In the fields of skin regeneration and esthetic medicine, EV‐based therapies are increasingly evolving toward precise functional intervention rather than simple tissue repair. Engineered EVs carrying antioxidant enzymes such as superoxide dismutase (SOD) have been shown to effectively eliminate reactive ROS in skin fibroblasts and restore ultraviolet‐induced collagen damage, highlighting their therapeutic potential for photoaging (Nguyen et al. 2024; Xu et al. 2020). In pathological scar models, adipose‐derived mesenchymal stem cell (ADSC)‐derived EVs enriched with miR‐29b suppress TGF‐β/Smad signaling and inhibit myofibroblast transdifferentiation, thereby attenuating hypertrophic scar formation (Ding et al. 2023; Zhang, Fukazawa, et al. 2024). EVs have also demonstrated promising regenerative effects in hair loss and skin barrier disorders. In androgenetic alopecia models, engineered EVs loaded with growth factors such as FGF2, particularly when combined with microneedle‐assisted delivery, significantly increased hair follicle density and outperformed conventional pharmacological therapies (Kim et al. 2025; Yu et al. 2025). In addition, EV‐mediated delivery of ceramide synthesis‐related mRNAs restored skin barrier integrity and reduced transepidermal water loss (TEWL) in atopic dermatitis models, suggesting potential applications in chronic inflammatory skin diseases (Kim, Lee, et al. 2026; You et al. 2025).

Beyond dermatological applications, EVs have shown considerable promise in the treatment of degenerative ocular diseases because of their ability to deliver therapeutic cargos with high precision and biocompatibility. In age‐related macular degeneration (AMD), EV‐based CRISPR/Cas9 systems targeting VEGFA successfully inhibited pathological retinal neovascularization (McAndrews et al. 2021). In glaucoma models, MSC‐derived EVs carrying miR‐21 protected retinal ganglion cells through activation of the PI3K/Akt survival pathway (van Poppelen et al. 2024). EVs have also demonstrated therapeutic potential in diabetic retinopathy and corneal injury by reducing vascular leakage, improving epithelial repair, and prolonging ocular surface retention through surface engineering strategies such as CD47 modification (Chen, Ma, et al. 2024; Yu, Zhao, et al. 2024). Furthermore, recent studies suggest that EV‐mediated delivery of molecular chaperones such as Hsp70 may restore proteostasis in retinal organoids, providing a potential therapeutic strategy for retinitis pigmentosa and other retinal degenerative disorders (Figure 4c).

Collectively, these findings highlight the versatility of EVs as multifunctional therapeutic nanoplatforms for regenerative dermatology and ophthalmology. Future studies should focus on improving tissue‐specific targeting, sustained delivery efficiency, and clinical‐grade manufacturing to facilitate the translation of EV‐based therapies into precision regenerative medicine and esthetic applications.

### 4.6 Key Challenges at the Application Level

Among current EV‐based therapeutic applications, neurodegenerative diseases have achieved the most advanced translational progress, with several studies already entering multicenter validation stages. Nevertheless, important limitations remain, particularly the lack of quantitative evidence regarding the actual brain delivery efficiency of intranasal EV administration. Similarly, aerosol inhalation strategies for pulmonary diseases represent an innovative delivery approach, yet most studies continue to rely on young animal models and fail to account for aging‐associated alterations in the lung microenvironment, such as mucus barrier thickening and impaired pulmonary clearance, which may substantially influence EV delivery efficiency.

In musculoskeletal diseases, osteoarthritis research has benefited from relatively standardized evaluation systems such as OARSI scoring, facilitating cross‐study comparisons. However, whether EVs can effectively penetrate deep cartilage layers following intra‐articular administration remains insufficiently resolved. In the fields of dermatology and ophthalmology, local delivery strategies are comparatively well established, but rigorous functional validation—such as long‐term restoration of skin barrier integrity or visual function—is still limited. Cardiovascular research currently possesses the strongest preclinical evidence base and the closest connection to early clinical translation. Nonetheless, the apparent discrepancy between the extremely low cardiac retention of EVs and their substantial therapeutic efficacy suggests that indirect systemic or immune‐mediated mechanisms may play underappreciated roles.

Overall, substantial differences remain among disease fields in terms of evidence quality, mechanistic clarity, and translational readiness. Future studies should therefore adopt more disease‐specific optimization strategies. Diagnostic applications require standardized clinical guidelines and biomarker validation frameworks, whereas therapeutic studies should more rigorously distinguish direct EV‐mediated effects from secondary systemic responses. In parallel, innovative delivery approaches must be supported by quantitative in vivo biodistribution and pharmacokinetic analyses. Most importantly, future translational studies should gradually shift from surrogate molecular endpoints toward clinically meaningful outcomes, including functional recovery, long‐term safety, and quality‐of‐life improvement.

Only through these methodological and translational upgrades can the true clinical value of EV‐based therapeutics be comprehensively evaluated and effectively translated into next‐generation precision anti‐aging medicine.

## 5 Tissue Targeting and Cellular Entry Properties of EVs

EVs are nanoscale membrane‐bound vesicles released by nearly all cell types under physiological and pathological conditions. By transporting diverse bioactive cargos including proteins, nucleic acids, lipids, and metabolites—they function as critical mediators of intercellular communication. In recent years, EVs have emerged as highly promising anti‐aging therapeutic platforms because of their excellent biocompatibility, low immunogenicity, capacity to cross biological barriers, and strong engineering potential. However, the therapeutic efficacy of EV‐based interventions depends largely on their ability to precisely target senescent tissues and diseased microenvironments.

Current evidence indicates that, following systemic administration, unmodified EVs are rapidly cleared by the mononuclear phagocyte system, particularly in the liver and spleen, with circulating half‐lives often shorter than 10 min. As a result, only a small fraction of administered EVs ultimately reaches target tissues, substantially limiting therapeutic efficiency. Therefore, understanding the mechanisms governing EV biodistribution, tissue tropism, and cellular uptake has become a central challenge for the clinical translation of EV‐based anti‐aging therapies.

Importantly, EV tropism is not entirely stochastic but is regulated by a complex “homing code” composed of surface proteins, lipids, glycans, and adhesion molecules inherited from parental cells.

These intrinsic targeting properties can also be further optimized through bioengineering approaches to achieve enhanced accumulation within aging‐associated tissues and pathological microenvironments. In the following sections, we systematically summarize the current understanding of natural EV targeting mechanisms and engineered targeting strategies, with particular emphasis on their translational potential for precision anti‐aging interventions (Table 2).

**TABLE 2:** Summary of targeting delivery strategies of EVs and their applications in aging‐related diseases.

Targeting category	Donor cell source	Specific targeting strategy	Target site	Disease/Aging scenario	References
Genetic engineering	Immature Dendritic Cells	LAMP2b‐RVG peptide modification	Cerebral neurons	Alzheimer's Disease (AD)	(Alvarez‐Erviti et al. 2011)
	HEK 293T cells		Aged vascular endothelium	Vascular aging and related diseases	(Paul et al. 2024)
Chemical modification	Mesenchymal stem cells	cRGD peptide	Ischemic myocardium/Endothelial cells	Geriatric myocardial hypertrophy and injury	(Fan et al. 2024)
Aptamer anchoring	Bone Marrow MSCs	AS1411 aptamer modification	Senescent cells with high nucleolin expression	Skin photoaging and cellular progeria	(Yan et al. 2023)
Membrane fusion/hybridization	Embryonic stem cells	cRGD‐Liposome hybridization	Inflammatory articular cartilage	Osteoarthritis and cartilage repair	(Sun et al. 2021)
Ligand‐receptor interaction	HEK293T cells	Apo‐A1 surface engineering	Hepatocytes (SR‐BI receptor)	Liver fibrosis and metabolic aging	(Li, Wang, et al. 2025)
Natural homing mechanism	Adipose‐derived MSCs	CXCR4 chemotactic axis	Tissue injury and inflammatory areas	Muscle atrophy and regenerative failure	(Kakade et al. 2025)
Physical guidance	Macrophages	SPIONs loading	Lesions locked by external magnetic field	Focal stroke and neurological repair	(Liu, Xia, et al. 2023)
Environmental response	Neutrophils	pH‐low insertion peptide	Inflammatory acidic microenvironment	Chronic inflammatory aging and osteoporosis	(Li, Wang, et al. 2025)

### 5.1 Natural Targeting Mechanisms

The natural targeting capability of EVs is largely determined by the biological characteristics of their parental cells. Surface molecular compositions inherited during vesicle biogenesis directly influence EV biodistribution, tissue tropism, and recipient‐cell recognition in vivo. Among the most important determinants are integrin expression patterns, tetraspanin composition, and membrane glycosylation signatures, all of which collectively contribute to the establishment of EV‐specific targeting properties.

#### 5.1.1 Integrin Profiles

Integrins are among the most important adhesion molecules governing EV targeting and tissue‐specific interactions. By mediating selective binding to extracellular matrix components and recipient‐cell receptors, integrins critically influence EV biodistribution and organ tropism. Pioneering large‐scale proteomic studies by Hoshino Atsushi and colleagues demonstrated that specific integrin combinations on tumor‐derived EVs are strongly associated with organ‐specific metastasis. For example, EVs expressing ITGα6β4 or ITGα6β1 preferentially accumulate in the lungs, where they activate the S100A8/A9–NF‐κB signaling axis and promote formation of a pro‐inflammatory pre‐metastatic niche. In contrast, EVs enriched in ITGαvβ5 exhibit liver tropism, whereas high ITGβ3 expression facilitates blood–brain barrier penetration (Hoshino et al. 2015). These findings established the conceptual framework that EV surface integrins function as molecular “zip codes” directing tissue‐specific homing.

Importantly, similar mechanisms are now being explored in the context of regenerative medicine and anti‐aging interventions. EVs derived from young tissues or mesenchymal stem cells often exhibit elevated ITGα4β1 expression, while aged vascular endothelial cells frequently upregulate VCAM‐1. This complementary interaction enhances EV accumulation within aging‐associated tissues such as the brain, heart, and skeletal muscle, effectively enabling “reverse‐engineered” pathological homing (Yousef et al. 2019). Likewise, induced pluripotent stem cell (iPSC)‐derived EVs expressing ITGαLβ2 have demonstrated preferential targeting to lymphoid tissues, where they modulate age‐associated immune dysfunction and immunosenescence (Munagala et al. 2016). These studies suggest that integrin signatures not only determine EV biodistribution but also actively participate in tissue‐specific functional regulation. Future engineering strategies aimed at optimizing integrin composition may therefore provide powerful approaches for enhancing the precision and therapeutic efficiency of EV‐based anti‐aging nanomedicine.

#### 5.1.2 Tetraspanins

Although tetraspanins such as CD9, CD63, and CD81 are widely recognized as canonical EV markers, their functions extend far beyond simple vesicle identification. By organizing membrane protein assemblies into specialized “tetraspanin‐enriched microdomains,” these molecules actively regulate EV biogenesis, membrane stability, cargo sorting, and recipient‐cell recognition (Andreu and Yanez‐Mo 2014). In addition, different tetraspanins contribute to distinct tissue‐targeting properties and biological functions. For example, CD9 is highly enriched in sperm‐derived EVs and plays an important role in sperm–egg fusion and reproductive cell communication (Aalberts et al. 2014).

Emerging evidence further suggests that tetraspanins participate in the coordination of receptor clustering and membrane fusion events, thereby influencing EV uptake efficiency and intracellular signaling in recipient cells. These findings indicate that tetraspanins are not merely structural markers, but functional regulators of EV targeting and intercellular communication, making them attractive candidates for future EV engineering and precision delivery strategies.

#### 5.1.3 Glycosylation Signatures

Surface glycan structures constitute another important layer of EV recognition signals that critically influence vesicle stability, biodistribution, and cellular interactions. Glycosylation patterns regulate the binding affinity of EVs to recipient cells and immune components, thereby shaping their in vivo fate. For example, high‐mannose N‐glycans can be recognized by mannose receptors expressed on macrophages, promoting rapid immune clearance, whereas α2,3‐linked sialylation enhances EV stability and reduces complement activation (Williams et al. 2018).

Distinct glycosylation signatures also contribute to tissue‐specific delivery properties. Milk‐derived EVs, for instance, are highly enriched in sialylated glycans that confer resistance to acidic environments and facilitate intestinal absorption, making them attractive natural carriers for oral drug delivery (Munagala et al. 2016). These findings highlight glycosylation not only as a determinant of EV biological behavior, but also as an important design element for future EV engineering strategies aimed at improving targeting efficiency, circulation stability, and non‐invasive delivery in anti‐aging therapeutics.

### 5.2 Engineered Targeting Strategies

To overcome the intrinsic limitations of natural EV targeting efficiency and biodistribution, a wide range of engineering strategies have been developed to endow EVs with enhanced or novel targeting capabilities. These approaches primarily involve genetic engineering, chemical surface modification, and physical or biomaterial‐assisted techniques, enabling more precise delivery of therapeutic cargos to specific tissues, cell populations, or aging‐associated pathological microenvironments.

This section primarily focuses on the engineering principles and surface modification strategies used to enhance EV targeting capability, whereas the following section emphasizes how these engineered systems improve delivery efficiency and therapeutic performance in aging‐associated diseases.

#### 5.2.1 Genetic Engineering for Ligand Display

To overcome the limitations of natural EV tropism, numerous engineering strategies have been developed to confer EVs with enhanced or entirely novel targeting capabilities through genetic, chemical, and physical modifications. Among these approaches, genetic engineering of parental cells remains the most widely used strategy. In this method, targeting peptides or proteins are fused with EV membrane anchor proteins, enabling their stable presentation on the EV surface. Commonly used anchor molecules include lysosome‐associated membrane protein 2b, PTGFRN, and CD63.

**FIGURE 5:**
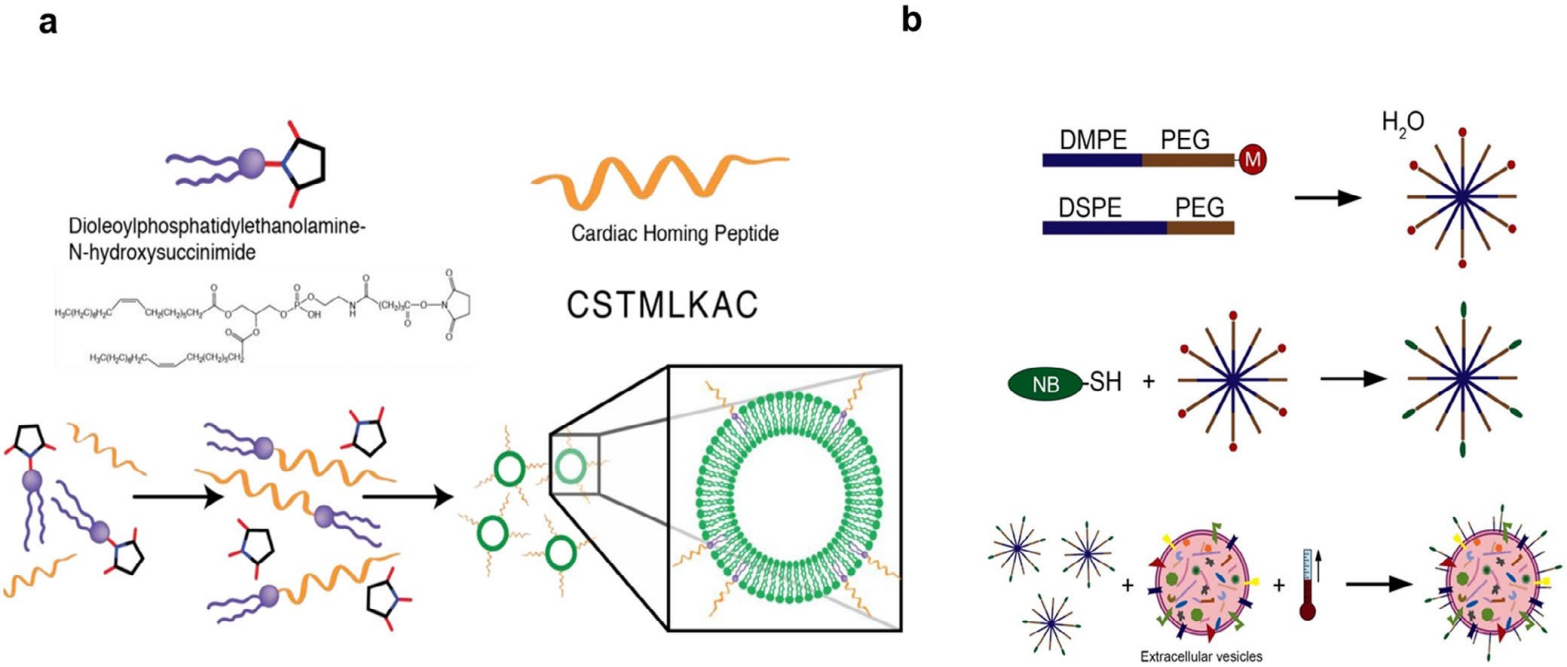
Engineering targeted strategies for extracellular vesicles. (a) The anti‐EGFR nanobody has a high affinity for epithelial cancer cells. Copyright 2018 Theranostics (b) The chemical technology is used to treat EVs, enabling efficient and specific connection of targeted molecules. Copyright 2016 Elsevier.

One of the most extensively studied examples involves fusion of the rabies virus glycoprotein (RVG) peptide with Lamp2b, which markedly enhances EV penetration across the blood–brain barrier and promotes neuronal targeting, particularly in AD models (Figure 5a) (Alvarez‐Erviti et al. 2011; Dooley et al. 2021). Similarly, incorporation of the cyclic RGD (cRGD) peptide enables selective recognition of αvβ3 integrins, thereby improving EV accumulation within tumors and ischemic tissues (Tian, Li, et al. 2014). Cardiac homing peptides have also been engineered onto EV membranes to facilitate targeted delivery to injured myocardium following ischemic damage (Figure 5b) (Vandergriff et al. 2018). In oncology‐related applications, anti‐EGFR nanobody‐modified EVs exhibit high affinity toward epithelial tumor cells, demonstrating the feasibility of antibody‐based precision targeting (Kooijmans et al. 2016).

Collectively, genetic engineering strategies provide highly programmable and relatively stable targeting capabilities, making them particularly attractive for chronic aging‐associated diseases that require precise tissue tropism, such as neurodegeneration, cardiovascular injury, and osteoporosis. However, the increased manufacturing complexity, potential immunogenicity of exogenous ligands, and challenges in large‐scale standardization remain important barriers for clinical translation.

#### 5.2.2 Chemical Conjugation and Lipid Insertion

Chemical surface modification strategies provide relatively simple and efficient approaches for rapidly enhancing EV targeting capability in vitro. Among these methods, click chemistry has emerged as one of the most widely used techniques because it enables highly specific conjugation of targeting ligands while largely preserving EV structural integrity and biological activity (Smyth et al. 2014). In addition, NHS‐ester and maleimide‐based coupling reactions are commonly employed to attach peptides or antibodies containing amine‐ or thiol‐functionalized groups to EV membranes (Armstrong et al. 2017). Another widely adopted approach is lipid insertion, in which hydrophobic interactions are used to incorporate ligand‐modified lipid molecules directly into the EV membrane. This strategy is technically straightforward and avoids complex genetic manipulation; however, the stability and long‐term retention of inserted molecules on the EV surface are often relatively limited (Apfelthaler et al. 2017; Kooijmans et al. 2016). Overall, chemical engineering methods offer flexible and scalable approaches for EV functionalization, particularly in short‐term targeting applications. Future optimization should focus on improving modification stability, minimizing unintended alterations of EV bioactivity, and integrating multifunctional targeting systems to enhance translational applicability in precision anti‐aging therapeutics.

#### 5.2.3 Membrane Fusion and Hybrid Technologies

Fusion of EVs with synthetic liposomes or cellular membranes has led to the development of hybrid vesicle systems that integrate the intrinsic bioactivity of natural EVs with the structural tunability of engineered nanomaterials. EV–liposome hybrids are particularly attractive because they combine efficient drug‐loading capacity with customizable targeting modifications, thereby enhancing both therapeutic payload delivery and tissue specificity (Piffoux et al. 2018). Membrane hybridization strategies can also introduce additional biological functions. For example, incorporation of red blood cell membranes provides EV hybrids with the “self‐recognition” molecule CD47, enabling reduced immune clearance and prolonged circulation time in vivo (Hu et al. 2021; Kamerkar et al. 2017). In parallel, fusion with cancer cell membranes preserves tumor‐associated antigens and facilitates the development of EV‐based cancer vaccines and immune‐targeting platforms (Fang et al. 2014).

Hybrid engineering strategies represent an important step toward the development of next‐generation intelligent delivery systems with enhanced targeting capability, immune evasion, and multifunctional therapeutic potential. Future advances integrating responsive biomaterials, programmable surface modifications, and precision cargo release mechanisms may further expand the application of hybrid EV platforms in anti‐aging nanomedicine and translational therapeutics.

### 5.3 Targeted Delivery, Efficiency Enhancement, and Cargo Loading

A major obstacle limiting the clinical translation of native EVs is their insufficient tissue tropism and rapid clearance by the reticuloendothelial system following systemic administration. To overcome these limitations, multidimensional surface engineering strategies have been developed to transform EVs into “smart” targeting nanoplatforms with enhanced homing capability and prolonged circulation time (Kim et al. 2021).

While early approaches primarily relied on genetic engineering to fuse targeting peptides with EV‐associated membrane proteins such as tetraspanins, more recent advances have introduced bio‐orthogonal chemistry as a flexible and minimally disruptive method for ligand conjugation. These strategies enable precise surface functionalization while preserving EV membrane integrity and biological activity. A representative example is the VesicleVoyager platform, which combines high‐throughput in vivo screening with peptide engineering to identify targeting motifs capable of directing EVs to highly specific tissue microenvironments and even sub‐organ regions (Kawai‐Harada et al. 2025).

In central nervous system disorders, engineered EVs displaying rabies virus glycoprotein (RVG) peptides or integrin‐binding ligands have demonstrated significantly improved blood–brain barrier penetration and enhanced accumulation within neural tissues (Lu, Hu, et al. 2022; Song, Liu, et al. 2023; Zhang, Li, Guan, et al. 2025; Zhang et al. 2021). These targeted EVs can selectively deliver therapeutic cargos to microglia and other brain‐resident cells, thereby improving the treatment efficiency of neurodegenerative diseases such as AD. Such BBB‐penetrating strategies are particularly valuable in aging‐associated neurodegenerative disorders, where inefficient drug transport into the central nervous system remains one of the major translational bottlenecks. Similar targeting principles have also been extended to other aging‐related disorders. For example, bone‐targeting EVs conjugated with aspartic acid octapeptides (Asp8) exhibit strong affinity for hydroxyapatite‐rich resorption sites, enabling precise delivery of therapeutic agents to osteoclast‐associated lesions in osteoporosis models. In contrast to neurodegenerative applications, bone‐targeting strategies emphasize local retention and sustained accumulation within mineralized tissues, highlighting how different aging‐associated diseases require distinct EV delivery paradigms. In renal diseases, EVs engineered with kidney injury molecule‐1 (KIM‐1)‐targeting peptides preferentially accumulate in damaged renal tubules and significantly reduce systemic toxicity compared with free‐drug administration.

In addition to active targeting strategies, immune‐evasive modifications have also attracted considerable attention. Surface functionalization with CD47, a canonical “don't eat me” signal, markedly decreases macrophage‐mediated phagocytosis and prolongs EV circulation time in vivo, thereby improving overall delivery efficiency (Hu, Qian, et al. 2024). Prolonging circulation time may be especially important for systemic anti‐aging interventions, in which repeated dosing and multi‐organ distribution are often required.

#### 5.3.1 Strategies for Efficiency Enhancement

A major limitation of EV‐based therapeutics is the extensive lysosomal degradation that occurs following cellular endocytosis, which substantially reduces the intracellular bioavailability of therapeutic cargos. To overcome this barrier, “endosomal escape” or “barrier‐breaking” engineering strategies have emerged as an important area of investigation. Recent studies have demonstrated that EV surface modification with pH‐responsive fusogenic peptides can destabilize endosomal membranes under acidic conditions, thereby enhancing cytosolic cargo release and significantly improving therapeutic efficiency (Dave et al. 2025; Kim, Hwang, et al. 2026). In parallel, hybrid vesicle systems generated through fusion of EVs with synthetic liposomes have been developed to optimize membrane lipid composition, resulting in improved colloidal stability, membrane fusion capability, and intracellular delivery performance (Xiong et al. 2023).

Beyond intracellular trafficking, considerable efforts have also focused on improving macroscopic delivery efficiency through biomaterial‐assisted and physical delivery technologies. Injectable hydrogel systems, for example, can function as localized “EV reservoirs,” enabling sustained release and prolonged retention within hostile pathological microenvironments. Such systems have shown promising therapeutic effects in bone repair and osteoimmune regulation, including the treatment of periprosthetic osteolysis (He et al. 2024; Wang, Ju, et al. 2025). In tissue engineering, three‐dimensional bioprinting technologies have further enabled the incorporation of EVs into bioactive scaffolds, which simultaneously provide structural support and sustained osteogenic signaling, thereby accelerating tissue regeneration compared with direct EV injection (Brunel et al. 2025).

Among physical enhancement strategies, focused ultrasound (FUS)‐mediated delivery has attracted particular attention because of its ability to transiently and noninvasively increase tissue permeability. When combined with microbubble‐assisted systems, FUS can facilitate EV penetration across dense biological barriers such as the blood–brain barrier and fibrotic tumor stroma, thereby overcoming the limitations of passive diffusion (Guan et al. 2025). Emerging evidence further suggests that ultrasound‐assisted approaches may provide broader therapeutic benefits in age‐related degenerative diseases by enhancing vascular permeability, increasing cellular uptake efficiency, modulating mechanotransduction pathways, and synergizing with biomaterial‐based delivery systems to achieve spatiotemporally controlled cargo release.

Despite these advances, several important challenges remain, including biosafety assessment, parameter standardization, tissue‐specific targeting accuracy, and scalability for clinical translation. Nevertheless, the integration of EV engineering with advanced biomaterials and physical stimulation technologies represents a highly promising direction for overcoming current delivery bottlenecks and improving the therapeutic performance of next‐generation anti‐aging nanomedicine platforms.

#### 5.3.2 Versatile Cargo Loading

The major advantage of engineered EVs lies in their unique capacity to encapsulate and deliver complex biological cargos that are difficult to efficiently transport using conventional synthetic nanocarriers. Through genetic engineering of EV‐associated scaffolds such as CD63, or by utilizing specific RNA‐binding protein systems, researchers can selectively enrich large nucleic acids—including therapeutic mRNAs encoding VEGF, collagen, or other regenerative proteins within EVs (Toms et al. 2023). Additionally, EVs loaded with Cas9 ribonucleoprotein complexes have demonstrated remarkable efficacy in editing pathological genes within neurodegenerative models. As well as nucleic acids, EVs are being engineered to carry hydrophobic senolytic drugs such as dasatinib or quercetin. These cargo‐loading strategies directly align with major hallmarks of aging, including cellular senescence, mitochondrial dysfunction, impaired regeneration, and chronic inflammation, thereby expanding EVs from passive carriers into multifunctional anti‐aging therapeutic platforms. By encapsulating these drugs within the lipid bilayer, the toxicity of the drugs to non‐target cells is significantly reduced and their ability to eliminate senescent cells in aged tissues is enhanced. The use of EVs in mitochondrial immunotherapy is an example of how the field of cargo loading is expanding. In this therapy, EVs derived from natural killer cells or stem cells transfer functional mitochondria to damaged cells, restoring oxidative phosphorylation in cases of ischaemia and metabolic disorders (Homberg et al. 2023; Wu, Feng, et al. 2025; Zhang, Zhang, Zhou, et al. 2025; Zhuang et al. 2024).

### 5.4 Cellular Entry Mechanisms

EVs play a crucial role in intercellular communication. Their therapeutic potential depends not only on the functional activity of their cargo, but also on their ability to efficiently enter target cells. EVs can be taken up by cells via various endocytic or fusion mechanisms. The efficiency, specificity, subcellular localisation, and subsequent fate of these pathways can differ significantly. Importantly, different uptake pathways critically determine whether EV cargos undergo lysosomal degradation or successfully escape into the cytoplasm, thereby directly influencing intracellular bioavailability, organelle targeting, and the therapeutic efficacy of anti‐aging cargos such as microRNAs, mRNAs, senolytic drugs, and mitochondrial regulatory molecules. The physical and chemical properties of EVs, the type of receptor cells, and the state of the microenvironment jointly regulate these mechanisms. These processes become particularly important in aging‐associated tissues, where chronic inflammation, oxidative stress, altered membrane fluidity, and dysfunctional endocytic activity may substantially reshape EV internalization behavior. A thorough understanding of the cellular entry mechanisms of EVs is crucial for optimizing their performance as anti‐aging delivery systems (Table 3).

**TABLE 3:** Internalization pathways of EVs and representative cellular models.

Uptake pathway	Key molecular mechanism	EV source	Target/Recipient cell	References
Clathrin‐mediated endocytosis (CME)	Invagination through AP‐2 and Dynamin‐dependent assembly	Melanoma cells	Macrophages/Epithelial cells	(Krylova and Feng 2023)
Caveolin‐mediated endocytosis (CvME)	Flask‐shaped structures dependent on lipid rafts and Caveolin‐1	Bone Marrow MSCs	Vascular endothelial cells/neurons	(Svensson et al. 2013)
Macropinocytosis	Non‐specific engulfment driven by Actin‐mediated plasma membrane ruffles	Glioma cells	Microglia/Dendritic cells	(Parada et al. 2021)
Membrane Fusion	Direct lipid bilayer fusion induced by SNAREs or acidic environments	Hepatic Stellate Cells	Hepatocytes	(Parolini et al. 2009)
Phagocytosis	Recognition by opsonins or Phosphatidylserine receptors	Red Blood Cells	Macrophages/Monocytes	(Patil et al. 2021)
Lipid Raft‐mediated Uptake	Non‐clathrin pathway dependent on cholesterol and Flotillin‐1	Mesenchymal Stem Cells	Fibroblasts/Immune cells	(Li et al. 2016)

#### 5.4.1 Clathrin‐Mediated Endocytosis

Clathrin‐mediated endocytosis (CME) is one of the best‐characterized pathways for EV internalization in eukaryotic cells. This process is initiated by the assembly of clathrin heavy and light chains into lattice‐like structures at the plasma membrane, followed by recruitment of adaptor protein complexes such as AP2 to form clathrin‐coated pits. Subsequent membrane invagination and vesicle scission are mediated by the GTPase dynamin, ultimately leading to intracellular uptake of EVs (Kaksonen and Roux 2018).

Accumulating evidence suggests that mesenchymal stem cell‐derived EVs (MSC‐EVs) are predominantly internalized by human umbilical vein endothelial cells through CME. Pharmacological inhibition of clathrin assembly using chlorpromazine markedly suppresses EV uptake and downstream VEGF signaling activation, confirming the importance of this pathway in EV‐mediated vascular repair (Chen, Tsai, et al. 2025; Tian, Zhu, et al. 2014). Importantly, aging‐associated reductions in clathrin expression and endocytic activity within senescent endothelial cells may impair CME efficiency, thereby limiting the regenerative potential of therapeutic EVs in aged tissues (Shin et al. 2021). Because many anti‐aging cargos including regulatory RNAs and mitochondrial protective molecules require cytoplasmic release to remain biologically active, excessive lysosomal trafficking following CME may substantially reduce therapeutic bioavailability.

Following CME‐mediated uptake, EVs are typically trafficked to early endosomes and subsequently directed toward lysosomal degradation pathways, which can substantially restrict the cytosolic release and bioavailability of therapeutic cargos (Hung and Leonard 2016). Therefore, strategies aimed at enhancing CME efficiency or promoting endosomal escape have become critical areas of investigation for improving the therapeutic performance of EV‐based anti‐aging delivery systems.

#### 5.4.2 Macropinocytosis

**FIGURE 6:**
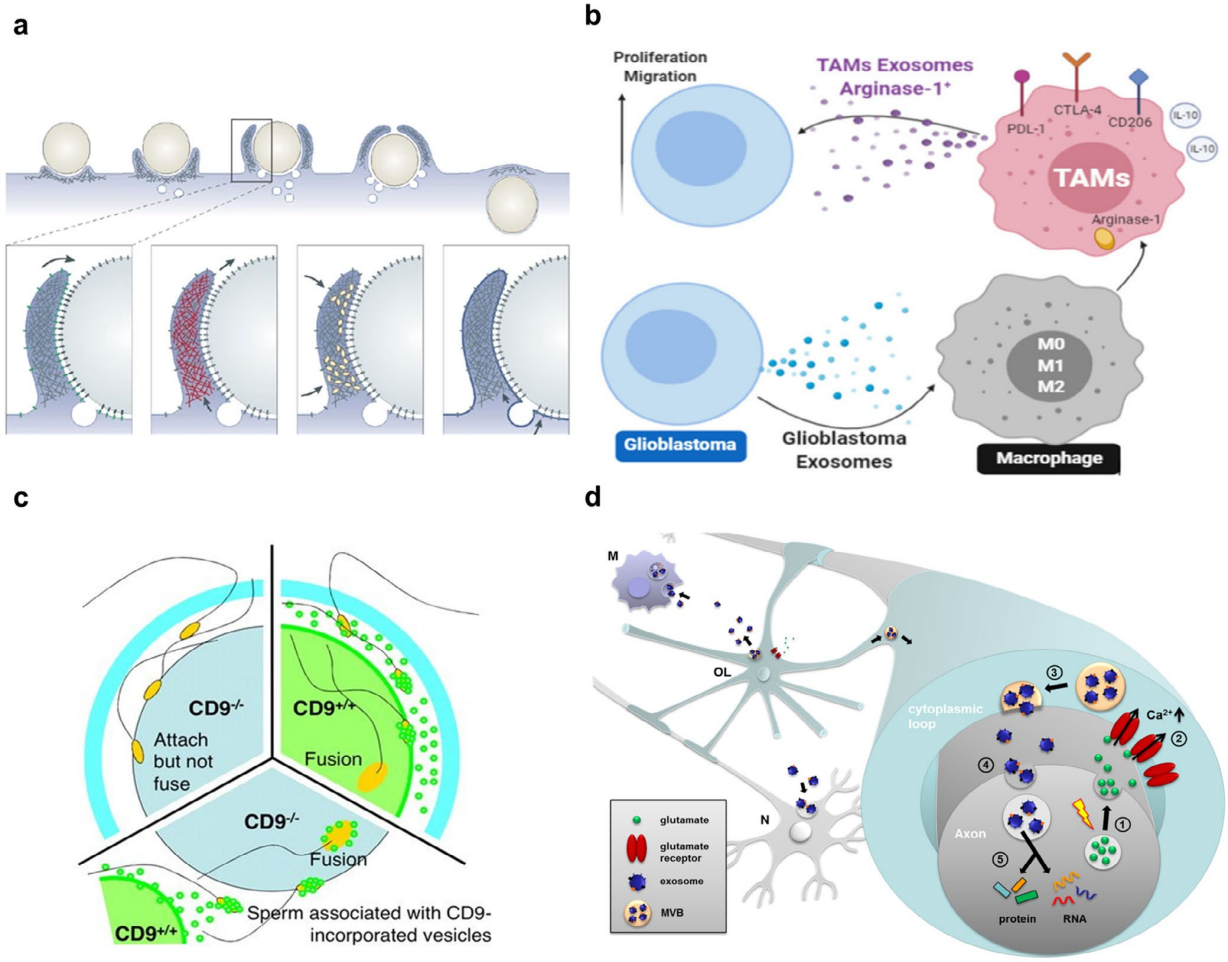
The mechanism of cell entry of extracellular vesicles. (a) Cellular phagocytosis is a non‐specific high‐capacity endocytic mode, in which membrane folds form large vesicles to engulf extracellular substances. Reproduced with permission (Swanson 2008). Copyright 2008 Springer Nature. (b) Tumor‐associated macrophages take up a large amount of EVs derived from tumors through phagocytosis and reprogram themselves to a tumor‐promoting phenotype. Reproduced with permission. (Azambuja et al. 2020) Copyright 2020 MDPI. (c) The process of sperm‐egg fusion involves the fusion of sperm exosomes with the integrin on the egg membrane through the interaction of CD9. Reproduced with permission (Miyado et al. 2008). Copyright 2008 The National Academy of Sciences of the United States of America. (d) Neuronal EVs directly deliver miRNAs to postsynaptic cells through fusion. Reproduced with permission (Fruhbeis et al. 2013). Copyright 2013 PLoS Biology.

Macropinocytosis is a non‐selective, actin‐dependent endocytic pathway characterized by extensive membrane ruffling and the formation of large intracellular vesicles known as macropinosomes. Unlike receptor‐mediated endocytosis, macropinocytosis enables the high‐capacity uptake of extracellular fluid and large quantities of EVs simultaneously. This process is particularly active in highly phagocytic or immune‐responsive cells, including macrophages, dendritic cells, and activated T cells (Figure 6a) (Koivusalo et al. 2010; Swanson 2008). Mechanistically, macropinocytosis is regulated by Rac1/Cdc42 GTPase activation, PI3K signaling, and Na^+^/H^+^ exchange activity, and can be specifically inhibited by the small‐molecule inhibitor EIPA.

Tumor‐associated macrophages, for example, can internalize large amounts of tumor‐derived EVs through macropinocytosis, resulting in metabolic and phenotypic reprogramming toward pro‐tumorigenic states (Figure 6b) (Whiteside et al. 2021; Zheng et al. 2017). In the context of aging, increased macropinocytic activity in aged macrophages may accelerate systemic clearance of therapeutic EVs, thereby reducing their circulation time and bioavailability (Canton 2018; Macedo et al. 2020). This phenomenon may be particularly relevant in inflammaging‐associated tissues, where chronically activated macrophages constitute a dominant barrier to systemic EVs delivery. However, this same property may also provide therapeutic opportunities. Enhanced uptake capacity of activated immune cells could potentially be exploited for targeted delivery of anti‐inflammatory cargos, enabling selective modulation of chronic inflammaging‐associated immune dysregulation.

#### 5.4.3 Direct Membrane Fusion

Direct membrane fusion represents a distinct EV entry mechanism in which the EV membrane merges directly with the plasma membrane of recipient cells, allowing immediate cytoplasmic release of therapeutic cargos without the formation of endocytic vesicles. Although this pathway occurs less frequently than endocytosis‐mediated uptake, it is particularly attractive for therapeutic delivery because it bypasses lysosomal degradation and substantially improves intracellular cargo bioavailability (Parolini et al. 2009). This property is particularly advantageous for the delivery of fragile therapeutic cargos such as mRNA, CRISPR systems, transcription factors, and mitochondrial regulatory molecules that require direct cytoplasmic access. Membrane fusion is highly dependent on compatible lipid composition, membrane fluidity, and the presence of specific fusion‐associated proteins. A classical example is observed during sperm–egg fusion, where sperm‐derived EVs interact with integrins on the oocyte membrane through CD9‐mediated mechanisms to facilitate membrane merging (Figure 6c) (Miyado et al. 2008). In the nervous system, neuronal EVs have also been shown to directly transfer microRNAs to postsynaptic cells via SNARE‐dependent fusion processes, enabling rapid intercellular signaling (Figure 6d) (Fruhbeis et al. 2013).

Importantly, microenvironmental conditions strongly influence fusion efficiency. Acidic conditions increase membrane fluidity and promote membrane fusion, whereas aging‐associated reductions in neuronal membrane flexibility may impair this process (Tian et al. 2013). Interestingly, pathological features of aged tissues—including chronic inflammation, oxidative stress, and local ROS accumulation—may paradoxically enhance EV fusion under certain conditions, thereby creating potential “pathological windows” for selective therapeutic targeting (Franceschi et al. 2018; Naudi et al. 2015).

#### 5.4.4 Phagocytosis

Phagocytosis is a specialized uptake mechanism primarily performed by professional phagocytes, including macrophages, monocytes, and microglia, for the clearance of apoptotic cells, pathogens, and large extracellular particles. Compared with smaller exosomes, this pathway predominantly mediates the uptake of larger EV populations such as microvesicles and apoptotic bodies exceeding approximately 500 nm in diameter (Imai et al. 2015). Recognition and internalization generally depend on Fc receptors, complement receptors, and pattern‐recognition receptors that identify opsonized or damage‐associated vesicles. Consequently, phagocytosis simultaneously represents a major cause of premature EV clearance and a potential mechanism for selective delivery to inflammatory immune populations in aging tissues.

In aging‐related tissues, phagocytic activity is often substantially altered. For example, age‐associated activation of microglia in the central nervous system may accelerate the clearance of neuroprotective EVs, thereby limiting their therapeutic persistence and reducing delivery efficiency within the aging brain (Hong et al. 2016). Conversely, this heightened phagocytic capacity may also be therapeutically advantageous, as engineered anti‐inflammatory EVs can be preferentially internalized by activated microglia or macrophages to suppress chronic neuroinflammation and inflammaging.

Therefore, phagocytosis represents both a major biological clearance barrier and a potential targeting mechanism for EV‐based therapeutics. Future strategies aimed at modulating phagocytic recognition such as CD47 surface functionalization, immune‐evasive membrane engineering, or selective phagocyte‐targeting approaches may help prolong EV circulation time while simultaneously improving tissue‐specific delivery and immunomodulatory efficacy in anti‐aging applications.

#### 5.4.5 Lipid Raft‐Mediated Endocytosis

Lipid raft‐mediated uptake represents a clathrin‐ and caveolin‐independent internalization pathway that relies on cholesterol‐ and sphingolipid‐rich membrane microdomains known as lipid rafts. These specialized membrane regions function as dynamic signaling platforms and frequently mediate the uptake of signaling complexes, membrane receptors, and EVs. Certain immune cell‐derived EVs utilize lipid raft‐dependent mechanisms to enter T cells and participate in immune synapse formation. Experimental depletion of membrane cholesterol using methyl‐β‐cyclodextrin significantly inhibits this process, highlighting the essential role of lipid raft integrity in EV internalization (Mittelbrunn et al. 2011). In addition, lipid rafts facilitate interactions between EVs and heparan sulfate proteoglycans (HSPGs), thereby enhancing EV uptake efficiency in hepatocytes, neurons, and other highly active signaling cells (Li et al. 2022). The disordered structure of lipid rafts in senescent cells may affect EVs recognition and internalization, emphasizing the importance of maintaining the stability of membrane microdomains for the effective delivery of EVs. Engineering EVs to preferentially interact with altered lipid raft compositions in senescent or inflamed cells may therefore improve tissue specificity and therapeutic selectivity in anti‐aging interventions.

### 5.5 Limitations and Prospects of Targeting and Entry Research

Although studies of natural EV tropism have identified several key targeting determinants—including integrin profiles, tetraspanin composition, and glycosylation signatures, most current evidence is derived from tumor models or experiments performed in young animals. However, aging tissues undergo substantial alterations in extracellular matrix composition, receptor expression, glycosylation enzyme activity, and membrane protein distribution. Consequently, a critical unresolved question remains: whether integrins displayed on EVs derived from young donors can still be efficiently recognized by ligands expressed in senescent target tissues.

In addition, many studies investigating natural targeting mechanisms rely heavily on simplified in vitro binding assays or artificial overexpression systems, which fail to accurately replicate the complex in vivo microenvironment, extracellular matrix barriers, and hemodynamic conditions. As a result, numerous targeting molecules that demonstrate promising specificity in vitro often exhibit markedly reduced targeting efficiency following systemic administration in vivo.

Engineered targeting strategies have further expanded the toolbox for EV modification, yet their translational potential may currently be overestimated. Surface engineering approaches using ligands such as rabies virus glycoprotein (RVG) peptides or cyclic arginine–glycine–aspartate (cRGD) peptides can indeed enhance EV accumulation in the brain or ischemic myocardium in mouse models. However, these modifications frequently alter additional biological properties simultaneously, including immunogenicity, circulation half‐life, and clearance kinetics. Importantly, most existing studies focus primarily on improvements in target‐organ accumulation while overlooking comprehensive biodistribution analyses, particularly regarding liver and spleen sequestration after engineering modifications.

More fundamentally, the complexity introduced by engineering modifications presents major challenges for large‐scale clinical manufacturing. Additional modification steps increase production variability, complicate quality control, and may substantially reduce reproducibility during industrial‐scale preparation. Despite the growing enthusiasm surrounding engineered EV therapeutics, these manufacturing and regulatory challenges have rarely been quantitatively evaluated in current literature.

Overall, future studies should place greater emphasis on evaluating targeting mechanisms within physiologically relevant aging models, systematically assessing the balance between targeting efficiency, immune clearance, and biosafety, and establishing standardized large‐scale manufacturing and quality‐control frameworks. Addressing these issues will be essential for translating engineered EV targeting strategies from proof‐of‐concept studies toward clinically viable anti‐aging therapeutics.

## 6 Scalable Manufacturing, Standardization, and Safety Control of EVs

### 6.1 Scalable Manufacturing Technologies

The clinical translation of EV‐based therapeutics requires a fundamental transition from small‐scale laboratory isolation toward standardized industrial‐scale manufacturing. Conventional static two‐dimensional culture systems are insufficient to meet the high production demands required for clinical dosing, particularly for systemic anti‐aging applications. To overcome these limitations, advanced culture platforms such as hollow‐fiber bioreactors and three‐dimensional microcarrier suspension systems have been widely adopted. By substantially increasing the surface area‐to‐volume ratio and improving nutrient exchange efficiency, these systems can enhance EV yield by approximately 10‐ to 40‐fold compared with traditional planar flask cultures, while largely preserving the immunomodulatory and regenerative functions of mesenchymal stem cell‐derived EVs (Haraszti et al. 2018; Todtenhaupt et al. 2023; Yang, Qiu, et al. 2025; Yin et al. 2025).

Beyond conventional culture optimization, bio‐inspired manufacturing strategies have recently emerged as promising approaches for further improving production efficiency. One notable example is the cell dehydration‐induced vesiculation strategy, in which osmotic stress triggers rapid membrane shedding and large‐scale release of engineered vesicles. Compared with passive physiological secretion, this approach dramatically increases production efficiency and offers a potentially scalable route for high‐yield EV manufacturing (Fiszer 2023; Liu et al. 2024).

In parallel, top‐down manufacturing technologies are gaining increasing attention as alternatives to natural EV biogenesis. Cell extrusion methods mechanically force cells through serial microfilters to generate exosome mimetics or cell‐derived nanovesicles, achieving production yields that can exceed natural exocytosis by more than 100‐fold (Yang, Li, et al. 2025). Importantly, these biomimetic vesicles retain key membrane proteins and targeting characteristics of their parental cells. For example, nanovesicles generated from engineered M2 macrophages maintain anti‐inflammatory targeting capabilities and have demonstrated strong therapeutic efficacy in inflammation modulation models.

In addition, metabolic engineering strategies within bioreactor systems have shown considerable promise for optimizing EV quality during large‐scale production. Manipulation of culture conditions, including glucose and glutamine supplementation, can influence intracellular metabolism and significantly improve cargo‐loading efficiency, thereby enhancing the therapeutic consistency of EV preparations.

Despite these technological advances, important challenges remain regarding batch‐to‐batch consistency, scalable purification, regulatory standardization, and preservation of biological activity during manufacturing. Future industrialization efforts should therefore focus not only on maximizing EV yield, but also on establishing reproducible quality‐control systems and functional evaluation standards to ensure the safety, stability, and clinical reliability of next‐generation EV therapeutics.

### 6.2 Standardization and Advanced Isolation Strategies

Establishing robust and standardized isolation and characterization systems is essential for reducing batch‐to‐batch variability during EV manufacturing and facilitating clinical translation. Although ultracentrifugation remains the most widely used isolation method in basic research, its limited scalability, low reproducibility, and tendency to induce vesicle aggregation or structural damage make it unsuitable for large‐scale Good Manufacturing Practice (GMP) production. To address these limitations, tangential flow filtration (TFF) has emerged as a leading strategy for industrial‐scale EV isolation. TFF enables gentle, size‐based separation while preserving vesicle integrity and biological activity, making it highly compatible with continuous large‐volume processing (Busatto et al. 2018; Li et al. 2017). To further improve purity, TFF is frequently combined with size exclusion chromatography (SEC), a complementary approach that efficiently removes soluble proteins, protein aggregates, and high‐density lipoprotein (HDL) contaminants from EV preparations (Auquiere et al. 2025).

In addition to conventional filtration‐based systems, several novel cost‐effective purification technologies have recently been developed. One promising example is the ion osmolarity‐driven sequential concentration strategy, which induces EV precipitation through controlled manipulation of ionic strength. This approach enables efficient recovery of EVs from large‐volume culture media without the use of potentially toxic polymer‐based precipitation reagents (Thakur et al. 2024).

At the forefront of standardization, microfluidic technologies are increasingly transforming EV manufacturing and quality control. Microfluidic platforms utilize mechanisms such as viscoelastic focusing and deterministic lateral displacement to continuously sort EVs according to size, deformability, and physical properties. These chip‐based systems enable automated production of highly uniform vesicle populations and are being integrated into “lab‐on‐a‐chip” platforms for rapid and standardized EV manufacturing (Hassanzadeh‐Barforoushi et al. 2025).

Beyond physical isolation, precise control of biological cargo composition is becoming equally important for next‐generation EV therapeutics. Recent studies have identified specific molecular “sorting codes” that regulate selective RNA packaging into EVs, providing a mechanistic basis for the standardized engineering of RNA‐enriched vesicles with predictable therapeutic properties (Abdelgawad et al. 2025; Favret et al. 2024).

### 6.3 Safety Control and Regulatory Compliance

Ensuring the biosafety of EV‐based therapeutics requires rigorous quality‐control systems that comply with international standards such as the MISEV2023 guidelines (Welsh et al. 2024). Among the major safety concerns, purification of drug‐loaded EVs remains particularly critical. During the encapsulation of chemotherapeutic agents, nucleic acids, or gene‐editing cargos, residual unencapsulated “free drugs” must be efficiently removed to avoid systemic toxicity and off‐target adverse effects. To address this challenge, advanced multimodal chromatography systems have been optimized for high‐resolution separation of therapeutic EVs from free cargo molecules and other contaminants (Auquiere et al. 2025; Corso et al. 2019; Dufil et al. 2023).

Another major obstacle is the intrinsic heterogeneity of EV populations. Conventional bulk characterization methods often fail to accurately distinguish therapeutically active vesicle subpopulations from inactive or potentially harmful particles. To overcome this limitation, emerging single‐vesicle analytical technologies now enable high‐resolution mapping of surface marker composition and cargo distribution at the individual particle level, thereby improving batch consistency and ensuring that only EV populations with defined therapeutic identities are selected for clinical use (Villamizar et al. 2021; Wang, Zhao, et al. 2024).

From a regulatory and manufacturing perspective, removal of endotoxins and host cell proteins (HCPs) is essential for minimizing immunogenicity and ensuring clinical safety. Current Good Manufacturing Practice (GMP)‐compatible workflows commonly integrate depth filtration, anion‐exchange chromatography, and multi‐step purification procedures to reduce these contaminants to levels acceptable for human administration (Gargett et al. 2019; Rohde et al. 2019; Witwer et al. 2019). In parallel, genome engineering technologies such as CRISPR‐Cas9 are being applied to producer cells to eliminate immunogenic membrane proteins or major histocompatibility complex‐related molecules, generating “universal donor” EVs with substantially reduced risks of immune rejection (Gargett et al. 2019; Hyldig et al. 2017).

Despite these advances, standardized release criteria for EV therapeutics remain incompletely defined. Future efforts should focus on establishing globally harmonized quality‐control frameworks encompassing vesicle identity, purity, potency, cargo consistency, immunogenicity, and long‐term biosafety evaluation. Such standardization will be essential for accelerating regulatory approval and enabling the reliable clinical translation of EV‐based anti‐aging therapeutics.

### 6.4 Limitations and Prospects of Production and Safety Control

The balance between large‐scale production and preservation of biological function represents one of the central challenges in EV industrialization. Although artificial induction strategies, such as cell extrusion, osmotic stress, and starvation‐mediated vesicle release can dramatically increase EV yield, systematic comparisons between these engineered vesicles and naturally secreted EVs remain insufficient. In particular, the effects of these production methods on membrane integrity, surface protein composition, lipid organization, and cargo stability have not been comprehensively evaluated. Mechanical stress generated during extrusion procedures may alter membrane protein conformation or induce lipid peroxidation, yet these potential structural damages are often overlooked by conventional characterization methods. Moreover, increased production efficiency is frequently achieved at the expense of cellular viability and physiological status. Because cellular stress and senescence can themselves alter vesicle composition and biological activity, EVs generated under artificial high‐yield conditions may differ substantially from physiologically secreted vesicles in terms of therapeutic function.

At the regulatory level, significant gaps also remain in current EV safety‐control systems. Although the MISEV2023 guidelines provide important recommendations for EV characterization and reporting, they do not establish specific release criteria or standardized thresholds for clinical‐grade products. For drug‐loaded EVs, there is currently no universally accepted quality‐control framework for evaluating residual free‐drug contamination. Likewise, endotoxin and host cell protein (HCP) limits are generally adapted from traditional biologic drug standards, despite the fact that EVs possess unique physicochemical properties as lipid nanoparticle‐based therapeutics. In addition, the long‐term biosafety of CRISPR‐engineered “universal donor” EVs remains largely unexplored.

A more fundamental concern is that current safety evaluations focus predominantly on vesicle composition itself while largely neglecting process‐related impurities introduced during industrial‐scale manufacturing. Potential contaminants including bioreactor leachates, residual microcarrier fragments, membrane extrusion debris, and other manufacturing‐derived nanoparticles are rarely incorporated into existing quality‐control systems despite their possible impact on immunogenicity and long‐term safety.

Future progress in this field will require several important methodological advances. First, large‐scale manufacturing studies should simultaneously evaluate both EV yield and functional potency, rather than treating production efficiency as the sole endpoint. Second, standardized equivalence criteria between engineered vesicles and naturally secreted EVs must be established to determine under which conditions artificial vesicles can serve as acceptable therapeutic substitutes. Third, the focus of standardization should gradually shift from purely physicochemical characterization toward functional subgroup screening and enrichment, supported by high‐throughput single‐vesicle functional analysis technologies. Finally, safety assessment systems should expand beyond vesicle characterization alone to include comprehensive monitoring of process‐related impurities and manufacturing‐associated risks, ultimately promoting the establishment of dedicated regulatory frameworks specifically designed for EV‐based therapeutics.

## 7 Challenges and Prospects of EVs

EVs have emerged as highly promising cell‐free therapeutic platforms in anti‐aging medicine owing to their excellent biocompatibility, low immunogenicity, intrinsic targeting capacity, and ability to cross biological barriers such as the blood–brain barrier. By transporting proteins, lipids, metabolites, and nucleic acids, EVs modulate multiple hallmarks of aging, including chronic inflammation, mitochondrial dysfunction, stem cell exhaustion, impaired proteostasis, and altered intercellular communication. Compared with live‐cell transplantation, EV‐based therapies exhibit lower risks of tumorigenicity and immune rejection while offering advantages in storage stability, manufacturing flexibility, and scalability.

Collectively, current clinical evidence suggests that EV‐based therapies exhibit favorable preliminary safety profiles and promising regenerative potential across multiple aging‐associated diseases. However, most studies remain limited to early‐stage trials with relatively small sample sizes and short follow‐up durations. In addition, substantial heterogeneity persists in EV source, isolation protocols, administration routes, dosage regimens, and therapeutic endpoints. Consequently, large‐scale randomized clinical trials and standardized GMP‐compatible manufacturing systems are urgently needed before EV therapeutics can achieve widespread clinical application.

Despite encouraging advances, several major barriers continue to limit the clinical translation of EVs. Current isolation and purification methods frequently generate batch‐to‐batch variability in vesicle size, cargo composition, and biological activity. Furthermore, most intravenously administered EVs are rapidly cleared by the mononuclear phagocyte system, resulting in short circulation times and insufficient accumulation within target tissues. The limited loading capacity of native EVs also restricts the efficient delivery of macromolecular therapeutics such as mRNA, CRISPR/Cas systems, and recombinant proteins. Therefore, improving manufacturing standardization, delivery efficiency, and cargo‐loading strategies remains a central objective for future development.

Biosafety also requires more systematic investigation. The frequently cited “low immunogenicity” of EVs should not be interpreted as universal safety, because EV‐associated risks are highly dependent on parental cell source, manufacturing procedures, dosage regimens, and recipient status. Tumor‐derived EVs may contain oncogenic or immunosuppressive cargoes, whereas long‐term toxicity data for MSC‐derived EVs remain limited. In addition, industrial‐scale production may introduce process‐related impurities, including endotoxins, residual host‐cell proteins, microcarrier debris, and free drug contamination. At present, standardized toxicological frameworks for evaluating chronic toxicity, tumorigenicity, immunogenicity variation, biodistribution, and off‐target accumulation are still lacking. With the gradual implementation of stricter regulatory policies, EV therapeutics are transitioning from exploratory research toward pharmaceutical‐grade quality control and regulatory supervision.

To overcome these limitations, EV engineering is evolving from the passive utilization of natural vesicles toward semi‐synthetic biomimetic systems and intelligent programmable nanoplatforms. Liposome–exosome hybrid vesicles, microfluidic‐generated nanovesicles, and stimulus‐responsive EV systems are being developed to improve production scalability, cargo‐loading efficiency, and controlled release performance. In parallel, emerging administration strategies—including inhalable dry powders, hydrogel‐assisted local delivery, and microneedle‐based transdermal systems—may further enhance tissue retention while reducing systemic clearance.

Importantly, the integration of multi‐omics technologies and artificial intelligence (AI) is expected to reshape the future design paradigm of EV therapeutics. Transcriptomics, proteomics, lipidomics, and glycomics enable comprehensive characterization of EV heterogeneity and identification of disease‐specific targeting signatures. AI‐driven prediction models may further optimize EV biodistribution, therapeutic efficacy, and biosafety. More notably, AI‐assisted protein design platforms such as AlphaFold and related deep‐learning frameworks may facilitate the de novo design of EV surface ligands with enhanced receptor specificity and tissue tropism. Such strategies could enable the construction of tissue‐specific “molecular postal codes” that selectively guide EVs toward aging‐associated pathological niches, including fibrotic lungs, inflamed vasculature, neurodegenerative lesions, and senescent stem cell compartments, thereby improving targeting precision while minimizing off‐target effects.

Another promising direction involves organoid‐derived EVs. Compared with conventional two‐dimensional cell cultures, organoids more accurately recapitulate native tissue architecture and aging‐associated microenvironments. Consequently, organoid‐derived EVs may better preserve physiologically relevant regenerative signals and disease‐specific molecular cargoes. Brain, lung, intestinal, and vascular organoid‐derived EVs are increasingly being explored for neurodegenerative diseases, pulmonary fibrosis, inflammatory disorders, and vascular aging. In addition, patient‐derived organoids may provide a foundation for personalized EV therapeutics.

Combination therapy is also emerging as an important translational strategy. EVs are increasingly being integrated with hydrogels, senolytic agents, CRISPR systems, mitochondrial transplantation technologies, and biomaterial‐based delivery platforms. Such combinational approaches may simultaneously enhance tissue regeneration, eliminate senescent cells, and remodel inflammatory microenvironments, thereby improving overall therapeutic efficacy.

Nevertheless, several conceptual and methodological issues remain unresolved. Most current studies still rely heavily on young animal models and short‐term observation periods, which insufficiently reflect the chronic and progressive nature of aging. Moreover, many reported therapeutic mechanisms remain correlative rather than causative because EVs contain highly complex molecular cargoes whose synergistic interactions are difficult to dissect using conventional single‐target approaches. Quantitative evidence regarding in vivo biodistribution, target‐organ retention, and intracellular cargo release is also still limited. Future studies should therefore prioritize standardized functional evaluation systems, long‐term aging models, and single‐vesicle tracking technologies capable of distinguishing direct therapeutic effects from indirect systemic responses.

Overall, the future clinical translation of EV‐based anti‐aging therapies will require coordinated advances in biosafety assessment, GMP‐compatible manufacturing, intelligent targeting design, and regulatory standardization. Future research should prioritize long‐term toxicological evaluation, AI‐assisted rational engineering, organ‐specific delivery strategies, and personalized therapeutic platforms. Through the integration of bioengineering, multi‐omics analysis, and artificial intelligence, EVs may evolve from experimental nanocarriers into clinically applicable precision therapeutics for aging‐associated diseases.

Recent years have witnessed the rapid clinical translation of EV‐based therapeutics for aging‐associated diseases. Compared with live‐cell transplantation, EVs exhibit several translational advantages, including lower immunogenicity, reduced tumorigenic risk, improved storage stability, and superior compatibility with industrial‐scale manufacturing. Multiple early‐phase clinical studies have evaluated EVs derived from MSCs, adipose‐derived stem cells, placental tissues, and umbilical cord‐derived cells in neurodegenerative disorders, pulmonary injury, metabolic dysfunction, renal fibrosis, and inflammatory diseases. Importantly, several EV‐based therapeutics have already entered phase I/II clinical evaluation, particularly for inflammatory and degenerative disorders associated with aging. Representative ongoing and completed clinical trials of EV‐based therapies are summarized in Table 4.

**TABLE 4:** Representative ongoing and completed clinical trials of EV‐based therapies for aging‐associated diseases.

Clinical condition	Clinical trial ID	EV source	Phase	Primary outcome measures	Status	References
COVID‐19‐associated lung injury	NCT04276987	Bone marrow MSC‐derived exosomes	Phase I	Oxygenation improvement, inflammatory markers	Completed	(Zhu et al. 2022)
Acute respiratory distress syndrome	NCT04313647	MSC‐derived exosomes	Phase I	Safety and pulmonary function	Completed	(Shi et al. 2021)
Severe COVID‐19 pneumonia	NCT04491240	ExoFlo (BM‐MSC exosomes)	Phase II	Cytokine reduction and survival improvement	Completed	(ClinicalTrials.gov 2020)
Age‐related renal fibrosis	NCT05043181	UC‐MSC‐derived EVs	Phase I/II	Renal function and safety	Recruiting	(ClinicalTrials.gov 2021a)
Type 1 diabetes mellitus	NCT02138331	MSC‐derived microvesicles/exosomes	Phase I	Safety and β‐cell preservation	Completed	(Ezquer et al. 2012)
Alzheimer's disease	NCT04388982	MSC‐derived EVs	Phase I/II	Cognitive assessment and biomarker changes	Recruiting	(Xie et al. 2023)
Dystrophic epidermolysis bullosa	NCT04173650	MSC‐derived exosomes	Phase I	Wound closure and safety	Not recruiting	(ClinicalTrials.gov 2019b)
Bronchopulmonary dysplasia	NCT03857841	MSC‐derived EVs	Phase I	Pulmonary safety and respiratory outcomes	Recruiting	(ClinicalTrials.gov 2019a)
Ischemic stroke	NCT03384433	Allogeneic MSC exosomes	Phase I/II	Neurological recovery and adverse events	Recruiting	(Aldali et al. 2025)
Dry eye disease	NCT04213248	Placental MSC‐derived EVs	Phase I	Ocular surface repair and inflammation reduction	Completed	(Randall Harrell et al. 2025)
Osteoarthritis	NCT05060107	Adipose MSC‐derived exosomes	Phase I/II	Pain relief and cartilage repair	Recruiting	(ClinicalTrials.gov 2021b)
Cutaneous wound healing	NCT02565264	Plasma‐derived exosomes	Early Phase I	Re‐epithelialization and safety	Completed	(ClinicalTrials.gov 2015)

## Author Contributions

X.H. performed validation and wrote the original draft. Q.L. developed the methodology and wrote, reviewed, and edited the final draft. G.T. performed the investigation and data curation. X.G. worked with the software. S.K. contributed to the visualization, data curation, and critical revision of the manuscript. L.S. and J.L. visualized the study and performed data curation.

## Funding

This work was supported by the National Natural Science Foundation of China Funds, 822770014, 82570028, 82000014. Open Fund of Key Laboratory of Artificial Organs and Computational Medicine in Zhejiang Province, SZD2025A002.

## Conflicts of Interest

The authors declare no conflicts of interest.

## Data Availability

The authors have nothing to report.

## References

[acel70607-bib-0001] Aalberts, M. , T. A. Stout , and W. Stoorvogel . 2014. “Prostasomes: Extracellular Vesicles From the Prostate.” Reproduction 147, no. 1: R1–R14. 10.1530/REP-13-0358. 24149515

[acel70607-bib-0002] Abdelgawad, A. , Y. Huang , O. Gololobova , et al. 2025. “Defining the Parameters for Sorting of RNA Cargo Into Extracellular Vesicles.” Journal of Extracellular Vesicles 14, no. 7: e70113. 10.1002/jev2.70113. 40620070 PMC12230367

[acel70607-bib-0003] Ala, M. 2023. “The Beneficial Effects of Mesenchymal Stem Cells and Their Exosomes on Myocardial Infarction and Critical Considerations for Enhancing Their Efficacy.” Ageing Research Reviews 89: 101980. 10.1016/j.arr.2023.101980. 37302757

[acel70607-bib-0004] Albakova, Z. , M. K. S. Siam , P. K. Sacitharan , R. H. Ziganshin , D. Y. Ryazantsev , and A. M. Sapozhnikov . 2021. “Extracellular Heat Shock Proteins and Cancer: New Perspectives.” Translational Oncology 14, no. 2: 100995. 10.1016/j.tranon.2020.100995. 33338880 PMC7749402

[acel70607-bib-0005] Aldali, F. , C. Deng , M. Nie , and H. Chen . 2025. “Advances in Therapies Using Mesenchymal Stem Cells and Their Exosomes for Treatment of Peripheral Nerve Injury: State of the Art and Future Perspectives.” Neural Regeneration Research 20, no. 11: 3151–3171. 10.4103/NRR.NRR-D-24-00235. 39435603 PMC11881730

[acel70607-bib-0006] Alvarez‐Erviti, L. , Y. Seow , H. Yin , C. Betts , S. Lakhal , and M. J. Wood . 2011. “Delivery of siRNA to the Mouse Brain by Systemic Injection of Targeted Exosomes.” Nature Biotechnology 29, no. 4: 341–345. 10.1038/nbt.1807. 21423189

[acel70607-bib-0007] Andreu, Z. , and M. Yanez‐Mo . 2014. “Tetraspanins in Extracellular Vesicle Formation and Function.” Frontiers in Immunology 5: 442. 10.3389/fimmu.2014.00442. 25278937 PMC4165315

[acel70607-bib-0008] Antich‐Rossello, M. , M. A. Forteza‐Genestra , M. Monjo , and J. M. Ramis . 2021. “Platelet‐Derived Extracellular Vesicles for Regenerative Medicine.” International Journal of Molecular Sciences 22, no. 16: 8580. 10.3390/ijms22168580. 34445286 PMC8395287

[acel70607-bib-0009] Apfelthaler, C. , M. Anzengruber , F. Gabor , and M. Wirth . 2017. “Poly‐(l)‐Glutamic Acid Drug Delivery System for the Intravesical Therapy of Bladder Cancer Using WGA as Targeting Moiety.” European Journal of Pharmaceutics and Biopharmaceutics 115: 131–139. 10.1016/j.ejpb.2017.02.016. 28237713

[acel70607-bib-0010] Armstrong, J. P. , M. N. Holme , and M. M. Stevens . 2017. “Re‐Engineering Extracellular Vesicles as Smart Nanoscale Therapeutics.” ACS Nano 11, no. 1: 69–83. 10.1021/acsnano.6b07607. 28068069 PMC5604727

[acel70607-bib-0011] Auquiere, M. , G. G. Muccioli , and A. des Rieux . 2025. “Methods and Challenges in Purifying Drug‐Loaded Extracellular Vesicles.” Journal of Extracellular Vesicles 14, no. 6: e70097. 10.1002/jev2.70097. 40527729 PMC12173533

[acel70607-bib-0012] Azambuja, J. H. , N. Ludwig , S. S. Yerneni , E. Braganhol , and T. L. Whiteside . 2020. “Arginase‐1+ Exosomes From Reprogrammed Macrophages Promote Glioblastoma Progression.” International Journal of Molecular Sciences 21, no. 11: 3990. 10.3390/ijms21113990. 32498400 PMC7312363

[acel70607-bib-0013] Babst, M. 2011. “MVB Vesicle Formation: ESCRT‐Dependent, ESCRT‐Independent and Everything in Between.” Current Opinion in Cell Biology 23, no. 4: 452–457. 10.1016/j.ceb.2011.04.008. 21570275 PMC3148405

[acel70607-bib-0014] Beg, M. A. , M. Huang , L. Vick , K. N. S. Rao , J. Zhang , and Y. Chen . 2024. “Targeting Mitochondrial Dynamics and Redox Regulation in Cardiovascular Diseases.” Trends in Pharmacological Sciences 45, no. 4: 290–303. 10.1016/j.tips.2024.02.001. 38458847 PMC11837222

[acel70607-bib-0015] Belhadj, J. , S. Surina , M. Hengstschlager , and A. J. Lomakin . 2023. “Form Follows Function: Nuclear Morphology as a Quantifiable Predictor of Cellular Senescence.” Aging Cell 22, no. 12: e14012. 10.1111/acel.14012. 37845808 PMC10726876

[acel70607-bib-0016] Berry, B. J. , and M. Kaeberlein . 2021. “An Energetics Perspective on Geroscience: Mitochondrial Protonmotive Force and Aging.” Geroscience 43, no. 4: 1591–1604. 10.1007/s11357-021-00365-7. 33864592 PMC8492883

[acel70607-bib-0017] Bhimavarapu, U. , N. Chintalapudi , and G. Battineni . 2024. “Automatic Detection and Classification of Hypertensive Retinopathy With Improved Convolution Neural Network and Improved SVM.” Bioengineering (Basel) 11, no. 1: 56. 10.3390/bioengineering11010056. 38247933 PMC10813404

[acel70607-bib-0018] Bolzan, A. D. 2021. “Mutagen‐Induced Telomere Instability in Human Cells.” Mutation Research, Genetic Toxicology and Environmental Mutagenesis 868‐869: 503387. 10.1016/j.mrgentox.2021.503387. 34454696

[acel70607-bib-0019] Bonetto, V. , and M. Grilli . 2023. “Neural Stem Cell‐Derived Extracellular Vesicles: Mini Players With Key Roles in Neurogenesis, Immunomodulation, Neuroprotection and Aging.” Frontiers in Molecular Biosciences 10: 1187263. 10.3389/fmolb.2023.1187263. 37228583 PMC10203560

[acel70607-bib-0020] Bonner, S. E. , S. I. van de Wakker , W. Phillips , et al. 2024. “Scalable Purification of Extracellular Vesicles With High Yield and Purity Using Multimodal Flowthrough Chromatography.” Journal of Extracellular Biology 3, no. 2: e138. 10.1002/jex2.138. 38939900 PMC11080796

[acel70607-bib-0021] Boulestreau, J. , M. Maumus , C. Jorgensen , and D. Noel . 2021. “Extracellular Vesicles From Mesenchymal Stromal Cells: Therapeutic Perspectives for Targeting Senescence in Osteoarthritis.” Advanced Drug Delivery Reviews 175: 113836. 10.1016/j.addr.2021.113836. 34166759

[acel70607-bib-0022] Brunel, L. G. , B. Cai , S. M. Hull , et al. 2025. “In Situ UNIversal Orthogonal Network (UNION) Bioink Deposition for Direct Delivery of Corneal Stromal Stem Cells to Corneal Wounds.” Bioactive Materials 48: 414–430. 10.1016/j.bioactmat.2025.02.009. 40083774 PMC11903395

[acel70607-bib-0023] Bui, V. D. , J. Jeon , V. H. Duong , et al. 2024. “Chondroitin Sulfate‐Based Microneedles for Transdermal Delivery of Stem Cell‐Derived Extracellular Vesicles to Treat Rheumatoid Arthritis.” Journal of Controlled Release 375: 105–115. 10.1016/j.jconrel.2024.08.050. 39218160

[acel70607-bib-0024] Busatto, S. , G. Vilanilam , T. Ticer , et al. 2018. “Tangential Flow Filtration for Highly Efficient Concentration of Extracellular Vesicles From Large Volumes of Fluid.” Cells 7, no. 12: 273. 10.3390/cells7120273. 30558352 PMC6315734

[acel70607-bib-0025] Buzas, E. I. 2023. “The Roles of Extracellular Vesicles in the Immune System.” Nature Reviews. Immunology 23, no. 4: 236–250. 10.1038/s41577-022-00763-8. PMC9361922 35927511

[acel70607-bib-0026] Byun, G. , J. Yang , and S. W. Seo . 2023. “CRISPRi‐Mediated Tunable Control of Gene Expression Level With Engineered Single‐Guide RNA in *Escherichia coli* .” Nucleic Acids Research 51, no. 9: 4650–4659. 10.1093/nar/gkad234. 36999618 PMC10201414

[acel70607-bib-0027] Canton, J. 2018. “Macropinocytosis: New Insights Into Its Underappreciated Role in Innate Immune Cell Surveillance.” Frontiers in Immunology 9: 2286. 10.3389/fimmu.2018.02286. 30333835 PMC6176211

[acel70607-bib-0028] Cao, L. , K. Li , Q. Li , Q. Tong , Y. Wang , and L. Huang . 2025. “The Controversial Role of Senescence‐Associated Secretory Phenotype (SASP) in Cancer Therapy.” Molecular Cancer 24, no. 1: 283. 10.1186/s12943-025-02475-8. 41204284 PMC12595836

[acel70607-bib-0029] Cassidy, L. D. , A. R. J. Young , C. N. J. Young , et al. 2020. “Temporal Inhibition of Autophagy Reveals Segmental Reversal of Ageing With Increased Cancer Risk.” Nature Communications 11, no. 1: 307. 10.1038/s41467-019-14187-x. PMC6965206 31949142

[acel70607-bib-0030] Chen, R. , S. Zhu , X. G. Fan , et al. 2018. “High Mobility Group Protein B1 Controls Liver Cancer Initiation Through Yes‐Associated Protein ‐Dependent Aerobic Glycolysis.” Hepatology 67, no. 5: 1823–1841. 10.1002/hep.29663. 29149457 PMC5906197

[acel70607-bib-0031] Chen, S. , T. Ma , J. Wang , et al. 2024. “Macrophage‐Derived Biomimetic Nanoparticles Enhanced SDT Combined With Immunotherapy Inhibited Tumor Growth and Metastasis.” Biomaterials 305: 122456. 10.1016/j.biomaterials.2023.122456. 38184961

[acel70607-bib-0033] Chen, X. , B. Tian , Y. Wang , J. Zheng , and X. Kang . 2025. “Potential and Challenges of Utilizing Exosomes in Osteoarthritis Therapy (Review).” International Journal of Molecular Medicine 55, no. 3: 43. 10.3892/ijmm.2025.5484. 39791222 PMC11759586

[acel70607-bib-0034] Chen, Y. , C. Y. Chen , H. Huang , et al. 2024. “Knocking Down of Xkr8 Enhances Chemotherapy Efficacy Through Modulating Tumor Immune Microenvironment.” Journal of Controlled Release 370: 479–489. 10.1016/j.jconrel.2024.03.011. 38685385 PMC11186464

[acel70607-bib-0035] Chen, Y. C. , W. C. Tsai , Z. X. Li , et al. 2025. “Exosomes From Human Umbilical Cord Mesenchymal Stem Cells Promote the Growth of Human Hair Dermal Papilla Cells.” PLoS One 20, no. 4: e0320154. 10.1371/journal.pone.0320154. 40305498 PMC12043141

[acel70607-bib-0036] Cheng, L. , and A. F. Hill . 2022. “Therapeutically Harnessing Extracellular Vesicles.” Nature Reviews. Drug Discovery 21, no. 5: 379–399. 10.1038/s41573-022-00410-w. 35236964

[acel70607-bib-0037] Chiba, M. , K. Miyata , H. Okawa , et al. 2023. “YBX1 Regulates Satellite II RNA Loading Into Small Extracellular Vesicles and Promotes the Senescent Phenotype.” International Journal of Molecular Sciences 24, no. 22: 16399. 10.3390/ijms242216399. 38003589 PMC10671301

[acel70607-bib-0038] Childs, B. G. , M. Gluscevic , D. J. Baker , et al. 2017. “Senescent Cells: An Emerging Target for Diseases of Ageing.” Nature Reviews. Drug Discovery 16, no. 10: 718–735. 10.1038/nrd.2017.116. 28729727 PMC5942225

[acel70607-bib-0039] Chitti, S. V. , S. Gummadi , T. Kang , et al. 2024. “Vesiclepedia 2024: An Extracellular Vesicles and Extracellular Particles Repository.” Nucleic Acids Research 52, no. D1: D1694–D1698. 10.1093/nar/gkad1007. 37953359 PMC10767981

[acel70607-bib-0041] ClinicalTrials.gov . 2015. “Effect of Plasma Derived Exosomes on Cutaneous Wound Healing (NCT02565264).” https://clinicaltrials.gov/study/NCT02565264.

[acel70607-bib-0045] ClinicalTrials.gov . 2019a. “A Safety Study of IV Stem Cell‐Derived Extracellular Vesicles (UNEX‐42) in Preterm Neonates at High Risk for BPD (NCT03857841).” https://clinicaltrials.gov/study/NCT03857841.

[acel70607-bib-0044] ClinicalTrials.gov . 2019b. “MSC EVs in Dystrophic Epidermolysis Bullosa (NCT04173650).” https://clinicaltrials.gov/study/NCT04173650.

[acel70607-bib-0040] ClinicalTrials.gov . 2020. “Evaluation of Safety and Efficiency of Method of Exosome Inhalation in SARS‐CoV‐2 Associated Pneumonia. (COVID‐19EXO) (NCT04491240).” https://clinicaltrials.gov/study/NCT04491240.

[acel70607-bib-0042] ClinicalTrials.gov . 2021a. “Exosome‐Based Nanoplatform for Ldlr mRNA Delivery in FH (ENDFH) (NCT05043181).” https://clinicaltrials.gov/study/NCT05043181.

[acel70607-bib-0043] ClinicalTrials.gov . 2021b. “Intra‐Articular Injection of MSC‐Derived Exosomes in Knee Osteoarthritis (ExoOA‐1) (NCT05060107).” https://clinicaltrials.gov/study/NCT05060107.

[acel70607-bib-0046] Conboy, I. M. , M. J. Conboy , A. J. Wagers , E. R. Girma , I. L. Weissman , and T. A. Rando . 2005. “Rejuvenation of Aged Progenitor Cells by Exposure to a Young Systemic Environment.” Nature 433, no. 7027: 760–764. 10.1038/nature03260. 15716955

[acel70607-bib-0047] Corso, G. , W. Heusermann , D. Trojer , et al. 2019. “Systematic Characterization of Extracellular Vesicle Sorting Domains and Quantification at the Single Molecule ‐ Single Vesicle Level by Fluorescence Correlation Spectroscopy and Single Particle Imaging.” Journal of Extracellular Vesicles 8, no. 1: 1663043. 10.1080/20013078.2019.1663043. 31579435 PMC6758720

[acel70607-bib-0048] Cox, M. J. , F. Lucien , R. Sakemura , et al. 2021. “Leukemic Extracellular Vesicles Induce Chimeric Antigen Receptor T Cell Dysfunction in Chronic Lymphocytic Leukemia.” Molecular Therapy 29, no. 4: 1529–1540. 10.1016/j.ymthe.2020.12.033. 33388419 PMC8058445

[acel70607-bib-0049] Crane, P. K. , C. Groot , R. Ossenkoppele , et al. 2024. “Cognitively Defined Alzheimer's Dementia Subgroups Have Distinct Atrophy Patterns.” Alzheimer's & Dementia 20, no. 3: 1739–1752. 10.1002/alz.13567. PMC10984445 38093529

[acel70607-bib-0050] Curvello, R. , N. Berndt , S. Hauser , and D. Loessner . 2024. “Recreating Metabolic Interactions of the Tumour Microenvironment.” Trends in Endocrinology and Metabolism 35, no. 6: 518–532. 10.1016/j.tem.2023.12.005. 38212233

[acel70607-bib-0051] D'Acunzo, P. , J. M. Ungania , Y. Kim , et al. 2023. “Cocaine Perturbs Mitovesicle Biology in the Brain.” Journal of Extracellular Vesicles 12, no. 1: e12301. 10.1002/jev2.12301. 36691887 PMC9871795

[acel70607-bib-0052] Dahlquist, K. J. V. , M. A. Huggins , M. J. Yousefzadeh , et al. 2024. “PD1 Blockade Improves Survival and CD8(+) Cytotoxic Capacity, Without Increasing Inflammation, During Normal Microbial Experience in Old Mice.” Nature Aging 4, no. 7: 915–925. 10.1038/s43587-024-00620-4. 38689133 PMC12142680

[acel70607-bib-0053] Dave, K. M. , P. P. Pinky , and S. Devika . 2025. “Molecular Engineering of Extracellular Vesicles for Drug Delivery: Strategies, Challenges, and Perspectives.” Journal of Controlled Release 386: 114068. 10.1016/j.jconrel.2025.114068. 40721069

[acel70607-bib-0054] De Sota, R. E. , S. R. Quake , J. J. Sninsky , and S. Toden . 2024. “Decoding Bioactive Signals of the RNA Secretome: The Cell‐Free Messenger RNA Catalogue.” Expert Reviews in Molecular Medicine 26: e12. 10.1017/erm.2024.12. 38682644 PMC11140549

[acel70607-bib-0055] DeBalsi, K. L. , K. E. Hoff , and W. C. Copeland . 2017. “Role of the Mitochondrial DNA Replication Machinery in Mitochondrial DNA Mutagenesis, Aging and Age‐Related Diseases.” Ageing Research Reviews 33: 89–104. 10.1016/j.arr.2016.04.006. 27143693 PMC5086445

[acel70607-bib-0056] Delabaere, L. , H. A. Ertl , D. J. Massey , et al. 2017. “Aging Impairs Double‐Strand Break Repair by Homologous Recombination in Drosophila Germ Cells.” Aging Cell 16, no. 2: 320–328. 10.1111/acel.12556. 28000382 PMC5334535

[acel70607-bib-0057] Detappe, A. , H. V. Nguyen , Y. Jiang , et al. 2023. “Author Correction: Molecular Bottlebrush Prodrugs as Mono‐ and Triplex Combination Therapies for Multiple Myeloma.” Nature Nanotechnology 18, no. 4: 419. 10.1038/s41565-023-01345-y. PMC10115626 36914884

[acel70607-bib-0058] Dietl, A. , A. Ralser , and K. Pelka . 2024. “Dual Checkpoint T(h1)eamwork Makes the Anti‐Cancer Dream Work.” Immunity 57, no. 3: 406–408. 10.1016/j.immuni.2024.02.015. 38479356

[acel70607-bib-0059] Ding, J. Y. , M. J. Chen , L. F. Wu , et al. 2023. “Mesenchymal Stem Cell‐Derived Extracellular Vesicles in Skin Wound Healing: Roles, Opportunities and Challenges.” Military Medical Research 10, no. 1: 36. 10.1186/s40779-023-00472-w. 37587531 PMC10433599

[acel70607-bib-0060] Ding, Z. , Z. Yan , X. Yuan , et al. 2024. “Apoptotic Extracellular Vesicles Derived From Hypoxia‐Preconditioned Mesenchymal Stem Cells Within a Modified Gelatine Hydrogel Promote Osteochondral Regeneration by Enhancing Stem Cell Activity and Regulating Immunity.” Journal of Nanobiotechnology 22, no. 1: 74. 10.1186/s12951-024-02333-7. 38395929 PMC10885680

[acel70607-bib-0061] Dooley, K. , R. E. McConnell , K. Xu , et al. 2021. “A Versatile Platform for Generating Engineered Extracellular Vesicles With Defined Therapeutic Properties.” Molecular Therapy 29, no. 5: 1729–1743. 10.1016/j.ymthe.2021.01.020. 33484965 PMC8116569

[acel70607-bib-0062] Douez, E. , E. Allard‐Vannier , I. A. M. Amar , et al. 2024. “Branched Pegylated Linker‐Auristatin to Control Hydrophobicity for the Production of Homogeneous Minibody‐Drug Conjugate Against HER2‐Positive Breast Cancer.” Journal of Controlled Release 366: 567–584. 10.1016/j.jconrel.2024.01.012. 38215985

[acel70607-bib-0063] Duan, X. , R. Zhang , H. Feng , et al. 2024. “A New Subtype of Artificial Cell‐Derived Vesicles From Dental Pulp Stem Cells With the Bioequivalence and Higher Acquisition Efficiency Compared to Extracellular Vesicles.” Journal of Extracellular Vesicles 13, no. 7: e12473. 10.1002/jev2.12473. 38965648 PMC11223992

[acel70607-bib-0064] Dufil, Y. , M. Dietrich , D. Zigah , et al. 2023. “Local Degradation of PEDOT:PSS on Silicon Nanostructures Using Scanning Electrochemical Microscopy.” Small 19, no. 10: e2206789. 10.1002/smll.202206789. 36543382

[acel70607-bib-0065] Dutra Silva, J. , Y. Su , C. S. Calfee , et al. 2021. “Mesenchymal Stromal Cell Extracellular Vesicles Rescue Mitochondrial Dysfunction and Improve Barrier Integrity in Clinically Relevant Models of ARDS.” European Respiratory Journal 58, no. 1: 2002978. 10.1183/13993003.02978-2020. 33334945 PMC8318599

[acel70607-bib-0066] El‐Awady, A. R. , M. Elashiry , A. C. Morandini , M. M. Meghil , and C. W. Cutler . 2022. “Dendritic Cells a Critical Link to Alveolar Bone Loss and Systemic Disease Risk in Periodontitis: Immunotherapeutic Implications.” Periodontology 2000 89, no. 1: 41–50. 10.1111/prd.12428. 35244951 PMC9018591

[acel70607-bib-0067] Ezquer, F. , M. Ezquer , D. Contador , M. Ricca , V. Simon , and P. Conget . 2012. “The Antidiabetic Effect of Mesenchymal Stem Cells Is Unrelated to Their Transdifferentiation Potential but to Their Capability to Restore Th1/Th2 Balance and to Modify the Pancreatic Microenvironment.” Stem Cells 30, no. 8: 1664–1674. 10.1002/stem.1132. 22644660

[acel70607-bib-0068] Fan, M. , X. Zhang , H. Liu , et al. 2024. “Reversing Immune Checkpoint Inhibitor‐Associated Cardiotoxicity via Bioorthogonal Metabolic Engineering‐Driven Extracellular Vesicle Redirecting.” Advanced Materials 36, no. 45: e2412340. 10.1002/adma.202412340. 39308257

[acel70607-bib-0069] Fang, R. H. , C. M. Hu , B. T. Luk , et al. 2014. “Cancer Cell Membrane‐Coated Nanoparticles for Anticancer Vaccination and Drug Delivery.” Nano Letters 14, no. 4: 2181–2188. 10.1021/nl500618u. 24673373 PMC3985711

[acel70607-bib-0070] Favret, J. , M. H. Nawaz , M. Patel , H. Khaledi , M. Gelb , and D. Shin . 2024. “Perinatal Loss of Galactosylceramidase in Both Oligodendrocytes and Microglia Is Crucial for the Pathogenesis of Krabbe Disease in Mice.” Molecular Therapy 32, no. 7: 2207–2222. 10.1016/j.ymthe.2024.05.019. 38734898 PMC11286809

[acel70607-bib-0071] Ferrucci, L. , F. Guerra , C. Bucci , E. Marzetti , and A. Picca . 2024. “Mitochondria Break Free: Mitochondria‐Derived Vesicles in Aging and Associated Conditions.” Ageing Research Reviews 102: 102549. 10.1016/j.arr.2024.102549. 39427885 PMC12155116

[acel70607-bib-0072] Fiorenzato, E. , S. Moaveninejad , L. Weis , R. Biundo , A. Antonini , and C. Porcaro . 2024. “Brain Dynamics Complexity as a Signature of Cognitive Decline in Parkinson's Disease.” Movement Disorders 39, no. 2: 305–317. 10.1002/mds.29678. 38054573

[acel70607-bib-0073] Firoozi, S. , S. Pahlavan , M. H. Ghanian , et al. 2020. “Mesenchymal Stem Cell‐Derived Extracellular Vesicles Alone or in Conjunction With a SDKP‐Conjugated Self‐Assembling Peptide Improve a Rat Model of Myocardial Infarction.” Biochemical and Biophysical Research Communications 524, no. 4: 903–909. 10.1016/j.bbrc.2020.02.009. 32057366

[acel70607-bib-0074] Fiszer, A. 2023. “All Roads Lead to Cure: Diversity of Oligonucleotides in DM1 Therapy.” Molecular Therapy ‐ Nucleic Acids 32: 898–899. 10.1016/j.omtn.2023.05.012. 37287495 PMC10242478

[acel70607-bib-0075] Fitz, N. F. , A. Sahu , Y. Lu , F. Ambrosio , I. Lefterov , and R. Koldamova . 2023. “Extracellular Vesicles in Young Serum Contribute to the Restoration of Age‐Related Brain Transcriptomes and Cognition in Old Mice.” International Journal of Molecular Sciences 24, no. 16: 12550. 10.3390/ijms241612550. 37628730 PMC10454174

[acel70607-bib-0076] Franceschi, C. , P. Garagnani , P. Parini , C. Giuliani , and A. Santoro . 2018. “Inflammaging: A New Immune‐Metabolic Viewpoint for Age‐Related Diseases.” Nature Reviews. Endocrinology 14, no. 10: 576–590. 10.1038/s41574-018-0059-4. 30046148

[acel70607-bib-0077] Fruhbeis, C. , D. Frohlich , W. P. Kuo , et al. 2013. “Neurotransmitter‐Triggered Transfer of Exosomes Mediates Oligodendrocyte‐Neuron Communication.” PLoS Biology 11, no. 7: e1001604. 10.1371/journal.pbio.1001604. 23874151 PMC3706306

[acel70607-bib-0078] Fukuda, M. 2025. “Mechanisms of Asymmetrical Exosome Release From Polarized Epithelial Cells: Implications for the Molecular Basis of Exosomal Heterogeneity.” In Extracellular Fine Particles, 27–38. Springer.

[acel70607-bib-0079] Garabedian, B. M. , E. E. Bashian , X. Wang , A. J. Thompson , and J. C. Paulson . 2025. “Targeting Sialidase to PD1 Enhances T Cell Function and Tumor Control.” ACS Central Science 11, no. 8: 1417–1427. 10.1021/acscentsci.5c00510. 40893963 PMC12395300

[acel70607-bib-0080] Gargett, T. , N. Truong , L. M. Ebert , W. Yu , and M. P. Brown . 2019. “Optimization of Manufacturing Conditions for Chimeric Antigen Receptor T Cells to Favor Cells With a Central Memory Phenotype.” Cytotherapy 21, no. 6: 593–602. 10.1016/j.jcyt.2019.03.003. 30975603

[acel70607-bib-0081] Gonzalez‐Rodriguez, A. , F. J. De Toro , A. Jorge‐Mora , et al. 2025. “Targeting Osteoarthritis With Small Extracellular Vesicle Therapy: Potential and Perspectives.” Frontiers in Bioengineering and Biotechnology 13: 1570526. 10.3389/fbioe.2025.1570526. 40621210 PMC12226547

[acel70607-bib-0082] Gorgun, C. , C. Africano , M. C. Ciferri , et al. 2022. “Preconditioned Mesenchymal Stromal Cell‐Derived Extracellular Vesicles (EVs) Counteract Inflammaging.” Cells 11, no. 22: 3695. 10.3390/cells11223695. 36429124 PMC9688039

[acel70607-bib-0083] Gray, D. E. , and F. N. Johnson . 1976. “Neuroticism, Extroversion, and Premenstrual Negative Affect.” IRCS Journal of Medical Science 4, no. 6: 284. 12334839

[acel70607-bib-0084] Guan, M. , X. Zhang , X. Li , et al. 2025. “Research Progress of Osteoarthritis Treatment by Low Intensity Pulsed Ultrasound.” Smart Medicine 4, no. 2: e70003. 10.1002/smmd.70003. 40390768 PMC12087415

[acel70607-bib-0085] Guo, F. , X. Liu , H. Cai , and W. Le . 2018. “Autophagy in Neurodegenerative Diseases: Pathogenesis and Therapy.” Brain Pathology 28, no. 1: 3–13. 10.1111/bpa.12545. 28703923 PMC5739982

[acel70607-bib-0086] Guo, J. , L. Lei , Y. Jin , L. Su , S. Cui , and L. Shi . 2026. “Engineering Immune Cell to Counteract Aging and Aging‐Associated Diseases.” Adv Sci (Weinh) 13, no. 14: e21776. 10.1002/advs.202521776. 41603194 PMC12970235

[acel70607-bib-0087] Gyorgy, B. , T. G. Szabo , M. Pasztoi , et al. 2011. “Membrane Vesicles, Current State‐of‐the‐Art: Emerging Role of Extracellular Vesicles.” Cellular and Molecular Life Sciences 68, no. 16: 2667–2688. 10.1007/s00018-011-0689-3. 21560073 PMC3142546

[acel70607-bib-0088] Han, J. , K. Xu , T. Xu , Q. Song , T. Duan , and J. Yang . 2025. “The Functional Regulation Between Extracellular Vesicles and the DNA Damage Responses.” Mutation Research, Reviews in Mutation Research 795: 108532. 10.1016/j.mrrev.2025.108532. 39828141

[acel70607-bib-0089] Haraszti, R. A. , R. Miller , M. Stoppato , et al. 2018. “Exosomes Produced From 3D Cultures of MSCs by Tangential Flow Filtration Show Higher Yield and Improved Activity.” Molecular Therapy 26, no. 12: 2838–2847. 10.1016/j.ymthe.2018.09.015. 30341012 PMC6277553

[acel70607-bib-0090] Hassanzadeh‐Barforoushi, A. , X. Sango , E. L. Johnston , D. Haylock , and Y. Wang . 2025. “Microfluidic Devices for Manufacture of Therapeutic Extracellular Vesicles: Advances and Opportunities.” Journal of Extracellular Vesicles 14, no. 7: e70132. 10.1002/jev2.70132. 40704561 PMC12287800

[acel70607-bib-0091] He, S. , Z. Li , L. Wang , et al. 2024. “A Nanoenzyme‐Modified Hydrogel Targets Macrophage Reprogramming‐Angiogenesis Crosstalk to Boost Diabetic Wound Repair.” Bioactive Materials 35: 17–30. 10.1016/j.bioactmat.2024.01.005. 38304915 PMC10831190

[acel70607-bib-0092] He, Y. , X. Wang , B. Xu , et al. 2025. “The Potential of Mitochondrial Transfer as the Modifying Therapy for Osteoarthritis.” Frontiers in Cell and Developmental Biology 13: 1643141. 10.3389/fcell.2025.1643141. 40932934 PMC12417883

[acel70607-bib-0093] Herrmann, I. K. , M. J. A. Wood , and G. Fuhrmann . 2021. “Extracellular Vesicles as a Next‐Generation Drug Delivery Platform.” Nature Nanotechnology 16, no. 7: 748–759. 10.1038/s41565-021-00931-2. 34211166

[acel70607-bib-0094] Hilal, H. , M. Haddadnezhad , M. J. Oh , I. Jung , and S. Park . 2024. “Plasmonic Dodecahedral‐Walled Elongated Nanoframes for Surface‐Enhanced Raman Spectroscopy.” Small 20, no. 3: e2304567. 10.1002/smll.202304567. 37688300

[acel70607-bib-0095] Homberg, B. , P. Rehling , and L. D. Cruz‐Zaragoza . 2023. “The Multifaceted Mitochondrial OXA Insertase.” Trends in Cell Biology 33, no. 9: 765–772. 10.1016/j.tcb.2023.02.001. 36863885

[acel70607-bib-0096] Hong, S. , V. F. Beja‐Glasser , B. M. Nfonoyim , et al. 2016. “Complement and Microglia Mediate Early Synapse Loss in Alzheimer Mouse Models.” Science 352, no. 6286: 712–716. 10.1126/science.aad8373. 27033548 PMC5094372

[acel70607-bib-0097] Horbay, R. , A. Hamraghani , L. Ermini , S. Holcik , S. T. Beug , and B. Yeganeh . 2022. “Role of Ceramides and Lysosomes in Extracellular Vesicle Biogenesis, Cargo Sorting and Release.” International Journal of Molecular Sciences 23, no. 23: 15317. 10.3390/ijms232315317. 36499644 PMC9735581

[acel70607-bib-0098] Hoshino, A. , B. Costa‐Silva , T. L. Shen , et al. 2015. “Tumour Exosome Integrins Determine Organotropic Metastasis.” Nature 527, no. 7578: 329–335. 10.1038/nature15756. 26524530 PMC4788391

[acel70607-bib-0099] Hou, D. , Q. Wu , S. Wang , et al. 2022. “Knockdown of miR‐214 Alleviates Renal Interstitial Fibrosis by Targeting the Regulation of the PTEN/PI3K/AKT Signalling Pathway.” Oxidative Medicine and Cellular Longevity 2022: 7553928. 10.1155/2022/7553928. 36285295 PMC9588363

[acel70607-bib-0100] Hu, B. , G. Liu , K. Zhao , and G. Zhang . 2024. “Diversity of Extracellular HSP70 in Cancer: Advancing From a Molecular Biomarker to a Novel Therapeutic Target.” Frontiers in Oncology 14: 1388999. 10.3389/fonc.2024.1388999. 38646439 PMC11026673

[acel70607-bib-0101] Hu, H. , H. Li , R. Li , P. Liu , and H. Liu . 2024. “Re‐Establishing Immune Tolerance in Multiple Sclerosis: Focusing on Novel Mechanisms of Mesenchymal Stem Cell Regulation of Th17/Treg Balance.” Journal of Translational Medicine 22, no. 1: 663. 10.1186/s12967-024-05450-x. 39010157 PMC11251255

[acel70607-bib-0102] Hu, S. , J. Ma , C. Su , et al. 2021. “Engineered Exosome‐Like Nanovesicles Suppress Tumor Growth by Reprogramming Tumor Microenvironment and Promoting Tumor Ferroptosis.” Acta Biomaterialia 135: 567–581. 10.1016/j.actbio.2021.09.003. 34506976

[acel70607-bib-0103] Hu, X. , C. He , L. Zhang , et al. 2023. “Mesenchymal Stem Cell‐Derived Exosomes Attenuate DNA Damage Response Induced by Cisplatin and Bleomycin.” Mutation Research, Genetic Toxicology and Environmental Mutagenesis 889: 503651. 10.1016/j.mrgentox.2023.503651. 37491116

[acel70607-bib-0104] Hu, Z. , S. Qian , Q. Zhao , et al. 2024. “Engineering Strategies for Apoptotic Bodies.” Smart Medicine 3, no. 3: e20240005. 10.1002/SMMD.20240005. 39420952 PMC11425054

[acel70607-bib-0105] Hua, T. , M. Yang , H. Song , et al. 2022. “Huc‐MSCs‐Derived Exosomes Attenuate Inflammatory Pain by Regulating Microglia Pyroptosis and Autophagy via the miR‐146a‐5p/TRAF6 Axis.” Journal of Nanobiotechnology 20, no. 1: 324. 10.1186/s12951-022-01522-6. 35836229 PMC9281091

[acel70607-bib-0106] Huan, Q. , S. Cheng , H. F. Ma , M. Zhao , Y. Chen , and X. Yuan . 2024. “Machine Learning‐Derived Identification of Prognostic Signature for Improving Prognosis and Drug Response in Patients With Ovarian Cancer.” Journal of Cellular and Molecular Medicine 28, no. 1: e18021. 10.1111/jcmm.18021. 37994489 PMC10805490

[acel70607-bib-0107] Huang, Z. , X. Liu , Q. Guo , et al. 2024. “Extracellular Vesicle‐Mediated Communication Between CD8^+^ Cytotoxic T Cells and Tumor Cells.” Frontiers in Immunology 15: 1376962. 10.3389/fimmu.2024.1376962. 38562940 PMC10982391

[acel70607-bib-0108] Hung, M. E. , and J. N. Leonard . 2016. “A Platform for Actively Loading Cargo RNA to Elucidate Limiting Steps in EV‐Mediated Delivery.” Journal of Extracellular Vesicles 5: 31027. 10.3402/jev.v5.31027. 27189348 PMC4870355

[acel70607-bib-0109] Hyenne, V. , M. Labouesse , and J. G. Goetz . 2018. “The Small GTPase Ral Orchestrates MVB Biogenesis and Exosome Secretion.” Small GTPases 9, no. 6: 445–451. 10.1080/21541248.2016.1251378. 27875100 PMC6204988

[acel70607-bib-0110] Hyldig, K. , S. Riis , C. P. Pennisi , V. Zachar , and T. Fink . 2017. “Implications of Extracellular Matrix Production by Adipose Tissue‐Derived Stem Cells for Development of Wound Healing Therapies.” International Journal of Molecular Sciences 18, no. 6: 1167. 10.3390/ijms18061167. 28561757 PMC5485991

[acel70607-bib-0111] Ikeda, G. , M. R. Santoso , Y. Tada , et al. 2021. “Mitochondria‐Rich Extracellular Vesicles From Autologous Stem Cell‐Derived Cardiomyocytes Restore Energetics of Ischemic Myocardium.” Journal of the American College of Cardiology 77, no. 8: 1073–1088. 10.1016/j.jacc.2020.12.060. 33632482 PMC8626617

[acel70607-bib-0112] Imai, T. , Y. Takahashi , M. Nishikawa , et al. 2015. “Macrophage‐Dependent Clearance of Systemically Administered B16BL6‐Derived Exosomes From the Blood Circulation in Mice.” Journal of Extracellular Vesicles 4: 26238. 10.3402/jev.v4.26238. 25669322 PMC4323410

[acel70607-bib-0113] Jeong, J. , J. K. Park , J. Shin , et al. 2025. “Inflammatory Cytokine‐Primed MSC‐Derived Extracellular Vesicles Ameliorate Acute Lung Injury via Enhanced Immunomodulation and Alveolar Repair.” Stem Cell Research & Therapy 16, no. 1: 450. 10.1186/s13287-025-04576-z. 40846969 PMC12374373

[acel70607-bib-0114] Jeppesen, D. K. , A. M. Fenix , J. L. Franklin , et al. 2019. “Reassessment of Exosome Composition.” Cell 177, no. 2: 428–445. 10.1016/j.cell.2019.02.029. 30951670 PMC6664447

[acel70607-bib-0116] Jiang, D. , F. Gao , Y. Zhang , et al. 2016. “Mitochondrial Transfer of Mesenchymal Stem Cells Effectively Protects Corneal Epithelial Cells From Mitochondrial Damage.” Cell Death & Disease 7, no. 11: e2467. 10.1038/cddis.2016.358. 27831562 PMC5260876

[acel70607-bib-0117] Kakade, D. , S. Date , V. Patole , et al. 2025. “Stem Cell‐Derived Exosomes in Wound Healing: Mechanistic Insights and Delivery Strategies.” Regenerative Medicine 20, no. 10: 527–553. 10.1080/17460751.2025.2561449. 41017563 PMC12562669

[acel70607-bib-0118] Kaksonen, M. , and A. Roux . 2018. “Mechanisms of Clathrin‐Mediated Endocytosis.” Nature Reviews. Molecular Cell Biology 19, no. 5: 313–326. 10.1038/nrm.2017.132. 29410531

[acel70607-bib-0119] Kamerkar, S. , V. S. LeBleu , H. Sugimoto , et al. 2017. “Exosomes Facilitate Therapeutic Targeting of Oncogenic KRAS in Pancreatic Cancer.” Nature 546, no. 7659: 498–503. 10.1038/nature22341. 28607485 PMC5538883

[acel70607-bib-0120] Katayama, M. , O. P. B. Wiklander , T. Fritz , et al. 2019. “Circulating Exosomal miR‐20b‐5p Is Elevated in Type 2 Diabetes and Could Impair Insulin Action in Human Skeletal Muscle.” Diabetes 68, no. 3: 515–526. 10.2337/db18-0470. 30552111

[acel70607-bib-0121] Kaushik, S. , I. Tasset , E. Arias , et al. 2021. “Autophagy and the Hallmarks of Aging.” Ageing Research Reviews 72: 101468. 10.1016/j.arr.2021.101468. 34563704 PMC8616816

[acel70607-bib-0122] Kawai‐Harada, Y. , M. Mardikoraem , A. V. Makela , et al. 2025. “VesicleVoyager: In Vivo Selection of Surface Displayed Proteins That Direct Extracellular Vesicles to Tissue‐Specific Targets.” Journal of Extracellular Vesicles 14, no. 11: e70184. 10.1002/jev2.70184. 41216961 PMC12603919

[acel70607-bib-0123] Kennedy, B. K. , S. L. Berger , A. Brunet , et al. 2014. “Geroscience: Linking Aging to Chronic Disease.” Cell 159, no. 4: 709–713. 10.1016/j.cell.2014.10.039. 25417146 PMC4852871

[acel70607-bib-0124] Kim, H. , J. H. Back , G. Han , et al. 2022. “Extracellular Vesicle‐Guided In Situ Reprogramming of Synovial Macrophages for the Treatment of Rheumatoid Arthritis.” Biomaterials 286: 121578. 10.1016/j.biomaterials.2022.121578. 35594838

[acel70607-bib-0125] Kim, J. , Y. H. Hwang , G. H. Nam , and I. S. Kim . 2026. “Breaking Barriers: Engineering Extracellular Vesicles for Enhanced Endosomal Escape and Therapeutic Delivery.” Journal of Controlled Release 389: 114462. 10.1016/j.jconrel.2025.114462. 41285243

[acel70607-bib-0126] Kim, J. W. , H. J. Lee , P. S. Chang , J. M. Lee , and J. C. Kim . 2026. “Hypoxic Conditioning Induced by CoCl_2_ Enhanced the Therapeutic Effects of Mesenchymal Stem Cell‐Derived Exosomes on Oxazolone‐Induced Atopic Dermatitis‐Like Skin Lesions in BALB/c Mice.” Molecular Immunology 189: 153–162. 10.1016/j.molimm.2025.12.008. 41411738

[acel70607-bib-0127] Kim, J. Y. , S. H. Kim , J. Seok , S. H. Bae , S. G. Hwang , and G. J. Kim . 2023. “Increased PRL‐1 in BM‐Derived MSCs Triggers Anaerobic Metabolism via Mitochondria in a Cholestatic Rat Model.” Molecular Therapy ‐ Nucleic Acids 31: 512–524. 10.1016/j.omtn.2023.01.017. 36865088 PMC9970868

[acel70607-bib-0128] Kim, J. Y. , W. K. Rhim , S. Y. Lee , et al. 2024. “Hybrid Nanoparticle Engineered With Transforming Growth Factor ‐beta1‐Overexpressed Extracellular Vesicle and Cartilage‐Targeted Anti‐Inflammatory Liposome for Osteoarthritis.” ACS Nano 18, no. 50: 33937–33952. 10.1021/acsnano.4c07992. 39648484 PMC11656835

[acel70607-bib-0129] Kim, W. , H. Y. Yoon , S. Lim , et al. 2021. “In Vivo Tracking of Bioorthogonally Labeled T‐Cells for Predicting Therapeutic Efficacy of Adoptive T‐Cell Therapy.” Journal of Controlled Release 329: 223–236. 10.1016/j.jconrel.2020.12.002. 33290794

[acel70607-bib-0130] Kim, Y. , S. E. Kim , K. D. Park , and K. M. Park . 2025. “Bioadhesives and Bioactive Hydrogels for Wound Management.” Journal of Controlled Release 379: 285–302. 10.1016/j.jconrel.2025.01.015. 39788376

[acel70607-bib-0131] Koivusalo, M. , C. Welch , H. Hayashi , et al. 2010. “Amiloride Inhibits Macropinocytosis by Lowering Submembranous pH and Preventing Rac1 and Cdc42 Signaling.” Journal of Cell Biology 188, no. 4: 547–563. 10.1083/jcb.200908086. 20156964 PMC2828922

[acel70607-bib-0132] Konig, T. , and H. M. McBride . 2024. “Mitochondrial‐Derived Vesicles in Metabolism, Disease, and Aging.” Cell Metabolism 36, no. 1: 21–35. 10.1016/j.cmet.2023.11.014. 38171335

[acel70607-bib-0133] Kooijmans, S. A. A. , L. A. L. Fliervoet , R. van der Meel , et al. 2016. “PEGylated and Targeted Extracellular Vesicles Display Enhanced Cell Specificity and Circulation Time.” Journal of Controlled Release 224: 77–85. 10.1016/j.jconrel.2016.01.009. 26773767

[acel70607-bib-0134] Krylova, S. V. , and D. Feng . 2023. “The Machinery of Exosomes: Biogenesis, Release, and Uptake.” International Journal of Molecular Sciences 24, no. 2: 1337. 10.3390/ijms24021337. 36674857 PMC9865891

[acel70607-bib-0135] Kumar, M. A. , S. K. Baba , H. Q. Sadida , et al. 2024. “Extracellular Vesicles as Tools and Targets in Therapy for Diseases.” Signal Transduction and Targeted Therapy 9, no. 1: 27. 10.1038/s41392-024-01735-1. 38311623 PMC10838959

[acel70607-bib-0136] Kumar, M. , S. Ray , and S. Sil . 2025. “Stem‐Cell‐Derived Extracellular Vesicles in Neurodegeneration and Neuroaging: Therapeutic Potential and Challenges.” Extracell Vesicles Circ Nucl Acids 6, no. 3: 594–608. 10.20517/evcna.2025.65. 41132506 PMC12540269

[acel70607-bib-0137] Kwon, H. J. , Y. Wu , Y. Li , et al. 2024. “On‐Demand Drug Delivery Bioelectronics Through a Water‐Processable Low Dimensional Highly Conductive MXene Layer.” Lab on a Chip 24, no. 13: 3294–3304. 10.1039/d4lc00234b. 38864519 PMC12066099

[acel70607-bib-0138] LeBar, K. , W. Liu , J. Pang , A. J. Chicco , and Z. Wang . 2024. “Role of the Microtubule Network in the Passive Anisotropic Viscoelasticity of Right Ventricle With Pulmonary Hypertension Progression.” Acta Biomaterialia 176: 293–303. 10.1016/j.actbio.2024.01.023. 38272197

[acel70607-bib-0139] Leedham, S. J. 2020. “Reserving the Right to Change the Intestinal Stem Cell Model.” Cell Stem Cell 26, no. 3: 301–302. 10.1016/j.stem.2020.02.003. 32142658 PMC7617120

[acel70607-bib-0140] Lejri, I. , Z. Cader , A. Grimm , and A. Eckert . 2024. “Human iPSCs From Aged Donors Retain Their Mitochondrial Aging Signature.” International Journal of Molecular Sciences 25, no. 20: 11199. 10.3390/ijms252011199. 39456998 PMC11508692

[acel70607-bib-0141] Li, F. X. , F. Xu , C. C. Li , et al. 2024. “Cold Exposure Alleviates T2DM Through Plasma‐Derived Extracellular Vesicles.” International Journal of Nanomedicine 19: 10077–10095. 10.2147/IJN.S441847. 39371478 PMC11456273

[acel70607-bib-0142] Li, L. , F. Wang , D. Zhu , S. Hu , K. Cheng , and Z. Li . 2025. “Engineering Exosomes and Exosome‐Like Nanovesicles for Improving Tissue Targeting and Retention.” Fundamental Research 5, no. 2: 851–867. 10.1016/j.fmre.2024.03.025. 40242543 PMC11997600

[acel70607-bib-0143] Li, P. , M. Kaslan , S. H. Lee , J. Yao , and Z. Gao . 2017. “Progress in Exosome Isolation Techniques.” Theranostics 7, no. 3: 789–804. 10.7150/thno.18133. 28255367 PMC5327650

[acel70607-bib-0144] Li, R. , C. Wang , M. Zhou , et al. 2022. “Heparan Sulfate Proteoglycan‐Mediated Internalization of Extracellular Vesicles Ameliorates Liver Fibrosis by Targeting Hepatic Stellate Cells.” Extracellular Vesicle 1: 100018. 10.1016/j.vesic.2022.100018.

[acel70607-bib-0145] Li, X. , W. Lu , L. Ni , J. Su , D. Wang , and Z. Deng . 2025. “Mitochondria‐Rich Extracellular Vesicles Derived From the Culture Supernatant of Human Synovial Fluid‐Derived Mesenchymal Stem Cells Inhibited Senescence of Stressed/Inflammatory Licensed Chondrocytes and Delayed Osteoarthritis Progression.” International Immunopharmacology 147: 113954. 10.1016/j.intimp.2024.113954. 39756162

[acel70607-bib-0146] Li, Y. , L. Gao , X. Tan , F. Li , M. Zhao , and S. Peng . 2016. “Lipid Rafts‐Mediated Endocytosis and Physiology‐Based Cell Membrane Traffic Models of Doxorubicin Liposomes.” Biochimica et Biophysica Acta 1858, no. 8: 1801–1811. 10.1016/j.bbamem.2016.04.014. 27117641

[acel70607-bib-0147] Li, Z. , H. Lu , L. Fan , et al. 2024. “Microneedle‐Delivered PDA@Exo for Multifaceted Osteoarthritis Treatment via PI3K‐Akt‐mTOR Pathway.” Advanced Science 11, no. 42: e2406942. 10.1002/advs.202406942. 39206714 PMC11558126

[acel70607-bib-0148] Liang, X. , Y. Zhang , F. Lin , et al. 2023. “Direct Administration of Mesenchymal Stem Cell‐Derived Mitochondria Improves Cardiac Function After Infarction via Ameliorating Endothelial Senescence.” Bioengineering & Translational Medicine 8, no. 1: e10365. 10.1002/btm2.10365. 36684073 PMC9842017

[acel70607-bib-0149] Lin, F. , H. Luo , J. Wang , Q. Li , and L. Zha . 2024. “Macrophage‐Derived Extracellular Vesicles as New Players in Chronic Non‐Communicable Diseases.” Frontiers in Immunology 15: 1479330. 10.3389/fimmu.2024.1479330. 39896803 PMC11782043

[acel70607-bib-0150] Liu, C. , D. Liu , S. Wang , L. Gan , X. Yang , and C. Ma . 2023. “Identification of the SNARE Complex That Mediates the Fusion of Multivesicular Bodies With the Plasma Membrane in Exosome Secretion.” Journal of Extracellular Vesicles 12, no. 9: e12356. 10.1002/jev2.12356. 37700095 PMC10497535

[acel70607-bib-0151] Liu, J. , T. Shen , Y. Zhang , et al. 2024. “Cell Dehydration Enables Massive Production of Engineered Membrane Vesicles With Therapeutic Functions.” Journal of Extracellular Vesicles 13, no. 7: e12483. 10.1002/jev2.12483. 39051765 PMC11270585

[acel70607-bib-0152] Liu, L. , S. Zhang , Y. Ren , et al. 2025. “Macrophage‐Derived Exosomes in Cancer: A Double‐Edged Sword With Therapeutic Potential.” Journal of Nanobiotechnology 23, no. 1: 319. 10.1186/s12951-025-03321-1. 40287762 PMC12034189

[acel70607-bib-0153] Liu, Z. , Q. Xia , D. Ma , et al. 2023. “Biomimetic Nanoparticles in Ischemic Stroke Therapy.” Discover Nano 18, no. 1: 40. 10.1186/s11671-023-03824-6. 36969494 PMC10027986

[acel70607-bib-0154] Lopez‐Otin, C. , M. A. Blasco , L. Partridge , M. Serrano , and G. Kroemer . 2013. “The Hallmarks of Aging.” Cell 153, no. 6: 1194–1217. 10.1016/j.cell.2013.05.039. 23746838 PMC3836174

[acel70607-bib-0155] Lopez‐Otin, C. , M. A. Blasco , L. Partridge , M. Serrano , and G. Kroemer . 2023. “Hallmarks of Aging: An Expanding Universe.” Cell 186, no. 2: 243–278. 10.1016/j.cell.2022.11.001. 36599349

[acel70607-bib-0157] Lu, T. , J. Zhang , J. Cai , et al. 2022. “Extracellular Vesicles Derived From Mesenchymal Stromal Cells as Nanotherapeutics for Liver Ischaemia‐Reperfusion Injury by Transferring Mitochondria to Modulate the Formation of Neutrophil Extracellular Traps.” Biomaterials 284: 121486. 10.1016/j.biomaterials.2022.121486. 35447404

[acel70607-bib-0158] Lu, W. , Z. Hu , X. Zhou , Y. Qin , Y. Zhang , and Y. Fang . 2022. “Natural Biopolymer Masks the Bitterness of Potassium Chloride to Achieve a Highly Efficient Salt Reduction for Future Foods.” Biomaterials 283: 121456. 10.1016/j.biomaterials.2022.121456. 35259583

[acel70607-bib-0159] Luo, D. , H. Zhu , S. Li , Z. Wang , and J. Xiao . 2024. “Mesenchymal Stem Cell‐Derived Exosomes as a Promising Cell‐Free Therapy for Knee Osteoarthritis.” Frontiers in Bioengineering and Biotechnology 12: 1309946. 10.3389/fbioe.2024.1309946. 38292826 PMC10824863

[acel70607-bib-0160] Luo, L. , J. Wang , J. Zhao , B. Yang , W. Ma , and J. Lin . 2025. “Dental Pulp Stem Cells Derived Exosomes Inhibit Ferroptosis via Regulating the Nrf2‐keap1/GPX4 Signaling Pathway to Ameliorate Chronic Kidney Disease Injury.” Tissue & Cell 93: 102670. 10.1016/j.tice.2024.102670. 39667244

[acel70607-bib-0161] Macedo, A. S. , P. M. Castro , L. Roque , et al. 2020. “Novel and Revisited Approaches in Nanoparticle Systems for Buccal Drug Delivery.” Journal of Controlled Release 320: 125–141. 10.1016/j.jconrel.2020.01.006. 31917295

[acel70607-bib-0162] Madhu, L. N. , M. Kodali , R. Upadhya , et al. 2024. “Extracellular Vesicles From Human‐Induced Pluripotent Stem Cell‐Derived Neural Stem Cells Alleviate Proinflammatory Cascades Within Disease‐Associated Microglia in Alzheimer's Disease.” Journal of Extracellular Vesicles 13, no. 11: e12519. 10.1002/jev2.12519. 39499013 PMC11536387

[acel70607-bib-0163] Mager, I. , X. O. Breakefield , and M. J. Wood . 2013. “Extracellular Vesicles: Biology and Emerging Therapeutic Opportunities.” Nature Reviews. Drug Discovery 12, no. 5: 347–357. 10.1038/nrd3978. 23584393

[acel70607-bib-0164] Mahindran, E. , W. S. Wan Kamarul Zaman , K. B. Ahmad Amin Noordin , Y. F. Tan , and F. Nordin . 2023. “Mesenchymal Stem Cell‐Derived Extracellular Vesicles: Hype or Hope for Skeletal Muscle Anti‐Frailty.” International Journal of Molecular Sciences 24, no. 9: 7833. 10.3390/ijms24097833. 37175537 PMC10178115

[acel70607-bib-0165] Mai, J. , Y. Ke , and Y. Yao . 2025. “Distinct Roles of Extracellular Vesicles Derived From Various Mesenchymal Stem Cell Sources in Bone Regeneration: A Systematic Review and Meta‐Analysis.” American Journal of Translational Research 17, no. 8: 5799–5813. 10.62347/WLNC8175. 40950314 PMC12432756

[acel70607-bib-0166] Mathieu, M. , L. Martin‐Jaular , G. Lavieu , and C. Thery . 2019. “Specificities of Secretion and Uptake of Exosomes and Other Extracellular Vesicles for Cell‐To‐Cell Communication.” Nature Cell Biology 21, no. 1: 9–17. 10.1038/s41556-018-0250-9. 30602770

[acel70607-bib-0167] McAndrews, K. M. , F. Xiao , A. Chronopoulos , V. S. LeBleu , F. G. Kugeratski , and R. Kalluri . 2021. “Exosome‐Mediated Delivery of CRISPR/Cas9 for Targeting of Oncogenic Kras(G12D) in Pancreatic Cancer.” Life Science Alliance 4, no. 9: e202000875. 10.26508/lsa.202000875. 34282051 PMC8321670

[acel70607-bib-0168] Medoro, A. , L. Saso , G. Scapagnini , and S. Davinelli . 2024. “NRF2 Signaling Pathway and Telomere Length in Aging and Age‐Related Diseases.” Molecular and Cellular Biochemistry 479, no. 10: 2597–2613. 10.1007/s11010-023-04878-x. 37917279 PMC11455797

[acel70607-bib-0169] Mehdipour, M. , C. Skinner , N. Wong , et al. 2020. “Rejuvenation of Three Germ Layers Tissues by Exchanging Old Blood Plasma With Saline‐Albumin.” Aging (Albany NY) 12, no. 10: 8790–8819. 10.18632/aging.103418. 32474458 PMC7288913

[acel70607-bib-0170] Meng, W. T. , J. Zhu , Y. C. Wang , et al. 2024. “Targeting Delivery of miR‐146a via IMTP Modified Milk Exosomes Exerted Cardioprotective Effects by Inhibiting NF‐kappaB Signaling Pathway After Myocardial Ischemia‐Reperfusion Injury.” Journal of Nanobiotechnology 22, no. 1: 382. 10.1186/s12951-024-02631-0. 38951872 PMC11218161

[acel70607-bib-0171] Mi, Z. , and S. H. Graham . 2023. “Role of UCHL1 in the Pathogenesis of Neurodegenerative Diseases and Brain Injury.” Ageing Research Reviews 86: 101856. 10.1016/j.arr.2023.101856. 36681249 PMC9992267

[acel70607-bib-0172] Mittelbrunn, M. , C. Gutierrez‐Vazquez , C. Villarroya‐Beltri , et al. 2011. “Unidirectional Transfer of microRNA‐Loaded Exosomes From T Cells to Antigen‐Presenting Cells.” Nature Communications 2: 282. 10.1038/ncomms1285. PMC3104548 21505438

[acel70607-bib-0173] Miyado, K. , K. Yoshida , K. Yamagata , et al. 2008. “The Fusing Ability of Sperm Is Bestowed by CD9‐Containing Vesicles Released From Eggs in Mice.” Proceedings of the National Academy of Sciences of the United States of America 105, no. 35: 12921–12926. 10.1073/pnas.0710608105. 18728192 PMC2525563

[acel70607-bib-0175] Morente‐Lopez, M. , R. Mato‐Basalo , S. Lucio‐Gallego , et al. 2022. “Therapy Free of Cells vs Human Mesenchymal Stem Cells From Umbilical Cord Stroma to Treat the Inflammation in OA.” Cellular and Molecular Life Sciences 79, no. 11: 557. 10.1007/s00018-022-04580-z. 36264388 PMC9584990

[acel70607-bib-0174] Morente‐Lopez, M. , R. Mato‐Basalo , S. Lucio‐Gallego , et al. 2023. “Effect of miR‐21 in Mesenchymal Stem Cells‐Derived Extracellular Vesicles Behavior.” Stem Cell Research & Therapy 14, no. 1: 383. 10.1186/s13287-023-03613-z. 38129923 PMC10740217

[acel70607-bib-0176] Munagala, R. , F. Aqil , J. Jeyabalan , and R. C. Gupta . 2016. “Bovine Milk‐Derived Exosomes for Drug Delivery.” Cancer Letters 371, no. 1: 48–61. 10.1016/j.canlet.2015.10.020. 26604130 PMC4706492

[acel70607-bib-0177] Naudi, A. , R. Cabre , M. Jove , et al. 2015. “Lipidomics of Human Brain Aging and Alzheimer's Disease Pathology.” International Review of Neurobiology 122: 133–189. 10.1016/bs.irn.2015.05.008. 26358893

[acel70607-bib-0178] Neary, M. T. , L. M. Mulder , P. S. Kowalski , R. MacLoughlin , A. M. Crean , and K. B. Ryan . 2024. “Nebulised Delivery of RNA Formulations to the Lungs: From Aerosol to Cytosol.” Journal of Controlled Release 366: 812–833. 10.1016/j.jconrel.2023.12.012. 38101753

[acel70607-bib-0179] Nguyen, D. D. N. , D. M. Vu , N. Vo , et al. 2024. “Skin Rejuvenation and Photoaging Protection Using Adipose‐Derived Stem Cell Extracellular Vesicles Loaded With Exogenous Cargos.” Skin Research and Technology 30, no. 2: e13599. 10.1111/srt.13599. 38279569 PMC10818134

[acel70607-bib-0180] O'Brien, C. M. , H. S. Chy , Q. Zhou , et al. 2017. “New Monoclonal Antibodies to Defined Cell Surface Proteins on Human Pluripotent Stem Cells.” Stem Cells 35, no. 3: 626–640. 10.1002/stem.2558. 28009074 PMC5412944

[acel70607-bib-0181] Ovcar, A. , and B. Kovacic . 2024. “Biogenesis of Extracellular Vesicles (EVs) and the Potential Use of Embryo‐Derived EVs in Medically Assisted Reproduction.” International Journal of Molecular Sciences 26, no. 1: 42. 10.3390/ijms26010042. 39795901 PMC11719982

[acel70607-bib-0182] Palama, M. E. F. , S. Coco , G. M. Shaw , et al. 2023. “Xeno‐Free Cultured Mesenchymal Stromal Cells Release Extracellular Vesicles With a “Therapeutic” miRNA Cargo Ameliorating Cartilage Inflammation In Vitro.” Theranostics 13, no. 5: 1470–1489. 10.7150/thno.77597. 37056573 PMC10086204

[acel70607-bib-0183] Panda, R. , and P. Kubes . 2023. “Extracellular Vesicles Selectively Mobilize Splenic Neutrophils.” Cardiovascular Research 119, no. 1: 1–2. 10.1093/cvr/cvad015. 36691968

[acel70607-bib-0184] Panfilova, A. , T. Zubareva , E. Mironova , et al. 2025. “Mitochondrial Proteins as Biomarkers of Cellular Senescence and Age‐Associated Diseases.” Aging (Albany NY) 17, no. 9: 2430–2448. 10.18632/aging.206305. 40856658 PMC12517210

[acel70607-bib-0185] Parada, N. , A. Romero‐Trujillo , N. Georges , and F. Alcayaga‐Miranda . 2021. “Camouflage Strategies for Therapeutic Exosomes Evasion From Phagocytosis.” Journal of Advanced Research 31: 61–74. 10.1016/j.jare.2021.01.001. 34194832 PMC8240105

[acel70607-bib-0186] Parolini, I. , C. Federici , C. Raggi , et al. 2009. “Microenvironmental pH Is a Key Factor for Exosome Traffic in Tumor Cells.” Journal of Biological Chemistry 284, no. 49: 34211–34222. 10.1074/jbc.M109.041152. 19801663 PMC2797191

[acel70607-bib-0187] Patil, M. , S. Saheera , P. K. Dubey , et al. 2021. “Novel Mechanisms of Exosome‐Mediated Phagocytosis of Dead Cells in Injured Heart.” Circulation Research 129, no. 11: 1006–1020. 10.1161/CIRCRESAHA.120.317900. 34623174 PMC8595793

[acel70607-bib-0188] Paul, S. , S. Bhagat , L. Dash , et al. 2024. “ExoDS: A Versatile Exosome‐Based Drug Delivery Platform to Target Cancer Cells and Cancer Stem Cells.” Frontiers in Bioengineering and Biotechnology 12: 1362681. 10.3389/fbioe.2024.1362681. 38903193 PMC11188490

[acel70607-bib-0189] Peng, D. , T. Liu , H. Lu , et al. 2024. “Intranasal Delivery of Engineered Extracellular Vesicles Loaded With miR‐206‐3p Antagomir Ameliorates Alzheimer's Disease Phenotypes.” Theranostics 14, no. 19: 7623–7644. 10.7150/thno.103596. 39659569 PMC11626949

[acel70607-bib-0190] Peng, Y. , T. Zhao , S. Rong , et al. 2024. “Young Small Extracellular Vesicles Rejuvenate Replicative Senescence by Remodeling Drp1 Translocation‐Mediated Mitochondrial Dynamics.” Journal of Nanobiotechnology 22, no. 1: 543. 10.1186/s12951-024-02818-5. 39238005 PMC11378612

[acel70607-bib-0191] Peruzzotti‐Jametti, L. , J. D. Bernstock , C. M. Willis , et al. 2021. “Neural Stem Cells Traffic Functional Mitochondria via Extracellular Vesicles.” PLoS Biology 19, no. 4: e3001166. 10.1371/journal.pbio.3001166. 33826607 PMC8055036

[acel70607-bib-0192] Phan, T. H. , S. Y. Kim , C. Rudge , and W. Chrzanowski . 2022. “Made by Cells for Cells ‐ Extracellular Vesicles as Next‐Generation Mainstream Medicines.” Journal of Cell Science 135, no. 1: jcs259166. 10.1242/jcs.259166. 35019142

[acel70607-bib-0193] Piffoux, M. , A. K. A. Silva , C. Wilhelm , F. Gazeau , and D. Tareste . 2018. “Modification of Extracellular Vesicles by Fusion With Liposomes for the Design of Personalized Biogenic Drug Delivery Systems.” ACS Nano 12, no. 7: 6830–6842. 10.1021/acsnano.8b02053. 29975503

[acel70607-bib-0194] Porcu, C. , G. Dobrowolny , and B. M. Scicchitano . 2024. “Exploring the Role of Extracellular Vesicles in Skeletal Muscle Regeneration.” International Journal of Molecular Sciences 25, no. 11: 5811. 10.3390/ijms25115811. 38892005 PMC11171935

[acel70607-bib-0195] Pulver, A. Y. , R. E. Tokmachev , N. A. Pulver , L. N. Antakova , and M. A. Emelianova . 2025. “Therapeutic Extracellular Vesicles as a Cornerstone of Medicine in the Next Decade With Gerontological Focus.” Biogerontology 26, no. 5: 190. 10.1007/s10522-025-10332-w. 41071355

[acel70607-bib-0196] Randall Harrell, C. , V. Djonov , A. Volarevic , A. Arsenijevic , and V. Volarevic . 2025. “Mesenchymal Stem Cell‐Sourced Exosomes as Potentially Novel Remedies for Severe Dry Eye Disease.” Journal of Ophthalmology 2025: 5552374. 10.1155/joph/5552374. 39839752 PMC11748739

[acel70607-bib-0197] Rao, S. , L. N. Madhu , R. S. Babu , et al. 2025. “Extracellular Vesicles From hiPSC‐Derived NSCs Protect Human Neurons Against Abeta‐42 Oligomers Induced Neurodegeneration, Mitochondrial Dysfunction and Tau Phosphorylation.” Stem Cell Research & Therapy 16, no. 1: 191. 10.1186/s13287-025-04324-3. 40251643 PMC12008877

[acel70607-bib-0198] Rather, H. A. , S. Almousa , S. Craft , and G. Deep . 2023. “Therapeutic Efficacy and Promise of Stem Cell‐Derived Extracellular Vesicles in Alzheimer's Disease and Other Aging‐Related Disorders.” Ageing Research Reviews 92: 102088. 10.1016/j.arr.2023.102088. 37827304 PMC10842260

[acel70607-bib-0199] Ribas‐Maynou, J. , A. Parra , P. Martinez‐Diaz , et al. 2025. “Protective Role of Extracellular Vesicles Against Oxidative DNA Damage.” Biological Research 58, no. 1: 14. 10.1186/s40659-025-00595-5. 40075425 PMC11905505

[acel70607-bib-0200] Ridzuan, N. , N. Zakaria , D. Widera , et al. 2021. “Human Umbilical Cord Mesenchymal Stem Cell‐Derived Extracellular Vesicles Ameliorate Airway Inflammation in a Rat Model of Chronic Obstructive Pulmonary Disease (COPD).” Stem Cell Research & Therapy 12, no. 1: 54. 10.1186/s13287-020-02088-6. 33436065 PMC7805108

[acel70607-bib-0201] Robbins, P. D. , and A. E. Morelli . 2014. “Regulation of Immune Responses by Extracellular Vesicles.” Nature Reviews. Immunology 14, no. 3: 195–208. 10.1038/nri3622. PMC4350779 24566916

[acel70607-bib-0202] Robinson, A. , D. Jiang , A. Nkansah , et al. 2025. “Advanced Manufacturing of Coil‐Reinforced Multilayer Vascular Grafts to Optimize Biomechanical Performance.” Acta Biomaterialia 198: 281–290. 10.1016/j.actbio.2025.04.020. 40216321

[acel70607-bib-0203] Rohde, E. , K. Pachler , and M. Gimona . 2019. “Manufacturing and Characterization of Extracellular Vesicles From Umbilical Cord‐Derived Mesenchymal Stromal Cells for Clinical Testing.” Cytotherapy 21, no. 6: 581–592. 10.1016/j.jcyt.2018.12.006. 30979664

[acel70607-bib-0204] Rudnitsky, E. , A. Braiman , M. Wolfson , et al. 2024. “Stem Cell‐Derived Extracellular Vesicles as Senotherapeutics.” Ageing Research Reviews 99: 102391. 10.1016/j.arr.2024.102391. 38914266

[acel70607-bib-0205] Sahoo, S. , and D. W. Losordo . 2014. “Exosomes and Cardiac Repair After Myocardial Infarction.” Circulation Research 114, no. 2: 333–344. 10.1161/CIRCRESAHA.114.300639. 24436429

[acel70607-bib-0206] Salehpour, A. , Z. Karimi , M. Ghasemi Zadeh , et al. 2024. “Therapeutic Potential of Mesenchymal Stem Cell‐Derived Exosomes and miRNAs in Neuronal Regeneration and Rejuvenation in Neurological Disorders: A Mini Review.” Frontiers in Cellular Neuroscience 18: 1427525. 10.3389/fncel.2024.1427525. 39429946 PMC11486650

[acel70607-bib-0207] Sang, Z. , P. Zhang , Y. Wei , and S. Dong . 2020. “miR‐214‐3p Attenuates Sepsis‐Induced Myocardial Dysfunction in Mice by Inhibiting Autophagy Through PTEN/AKT/mTOR Pathway.” BioMed Research International 2020: 1409038. 10.1155/2020/1409038. 32714974 PMC7359738

[acel70607-bib-0208] Schaaf, K. R. , S. R. Landstreet , S. Pugazenthi , et al. 2024. “Cell‐Free Hemoglobin Triggers Macrophage Cytokine Production via TLR4 and MyD88.” American Journal of Physiology. Lung Cellular and Molecular Physiology 326, no. 1: L29–L38. 10.1152/ajplung.00123.2023. 37991487 PMC11279742

[acel70607-bib-0209] Shi, M. M. , Q. Y. Yang , A. Monsel , et al. 2021. “Preclinical Efficacy and Clinical Safety of Clinical‐Grade Nebulized Allogenic Adipose Mesenchymal Stromal Cells‐Derived Extracellular Vesicles.” Journal of Extracellular Vesicles 10, no. 10: e12134. 10.1002/jev2.12134. 34429860 PMC8363910

[acel70607-bib-0210] Shin, E. Y. , N. K. Soung , M. A. Schwartz , and E. G. Kim . 2021. “Altered Endocytosis in Cellular Senescence.” Ageing Research Reviews 68: 101332. 10.1016/j.arr.2021.101332. 33753287 PMC8131247

[acel70607-bib-0315] Shou, X. , C. Chen , H. Ying , et al. 2025. “Biomimetic MOF Nanocarrier‐Mediated Synergistic Delivery of Mitochondria and Anti‐Inflammatory miRNA to Alleviate Acute Lung Injury.” Advanced Science 12, no. 16: 2416594. 10.1002/advs.202416594. 39999302 PMC12021094

[acel70607-bib-0211] Silva, F. , S. Pinto , S. G. Santos , F. D. Magalhaes , B. Sarmento , and A. M. Pinto . 2024. “New Graphene‐Containing Pharmaceutical Formulations for Infrared Lamps‐Based Phototherapy of Skin Cancer: In Vitro Validation and Ex‐Vivo Human Skin Permeation.” Nanomedicine 57: 102734. 10.1016/j.nano.2024.102734. 38295912

[acel70607-bib-0212] Sinha, J. K. , K. Jorwal , K. K. Singh , S. S. Han , R. Bhaskar , and S. Ghosh . 2025. “The Potential of Mitochondrial Therapeutics in the Treatment of Oxidative Stress and Inflammation in Aging.” Molecular Neurobiology 62, no. 6: 6748–6763. 10.1007/s12035-024-04474-0. 39230868

[acel70607-bib-0213] Skowyra, M. L. , P. Feng , and T. A. Rapoport . 2024. “Towards Solving the Mystery of Peroxisomal Matrix Protein Import.” Trends in Cell Biology 34, no. 5: 388–405. 10.1016/j.tcb.2023.08.005. 37743160 PMC10957506

[acel70607-bib-0214] Smyth, T. , K. Petrova , N. M. Payton , et al. 2014. “Surface Functionalization of Exosomes Using Click Chemistry.” Bioconjugate Chemistry 25, no. 10: 1777–1784. 10.1021/bc500291r. 25220352 PMC4198107

[acel70607-bib-0215] Somasundaram, I. , S. M. Jain , M. Blot‐Chabaud , et al. 2024. “Mitochondrial Dysfunction and Its Association With Age‐Related Disorders.” Frontiers in Physiology 15: 1384966. 10.3389/fphys.2024.1384966. 39015222 PMC11250148

[acel70607-bib-0216] Song, J. , J. Liu , C. Cui , et al. 2023. “Mesenchymal Stromal Cells Ameliorate Diabetes‐Induced Muscle Atrophy Through Exosomes by Enhancing AMPK/ULK1‐Mediated Autophagy.” Journal of Cachexia, Sarcopenia and Muscle 14, no. 2: 915–929. 10.1002/jcsm.13177. 36708027 PMC10067482

[acel70607-bib-0218] Stanczak, M. A. , and E. L. Pearce . 2024. “Please Don't Go: Retinoic Acid ‘retains’ Tissue‐Specific Memory.” Trends in Immunology 45, no. 12: 920–921. 10.1016/j.it.2024.11.005. 39572339 PMC11902903

[acel70607-bib-0219] Suades, R. , M. F. Greco , P. Prieto , et al. 2024. “CD66b^+^/CD68^+^ Circulating Extracellular Vesicles, Lactate Dehydrogenase and Neutrophil‐To‐Lymphocyte Ratio Can Differentiate Coronavirus Disease 2019 Severity During and After Infection.” Journal of Extracellular Vesicles 13, no. 7: e12456. 10.1002/jev2.12456. 39007437 PMC11247396

[acel70607-bib-0220] Sun, Y. , G. Liu , K. Zhang , Q. Cao , T. Liu , and J. Li . 2021. “Mesenchymal Stem Cells‐Derived Exosomes for Drug Delivery.” Stem Cell Res Ther 12, no. 1: 561. 10.1186/s13287-021-02629-7. 34717769 PMC8557580

[acel70607-bib-0221] Svensson, K. J. , H. C. Christianson , A. Wittrup , et al. 2013. “Exosome Uptake Depends on ERK1/2‐Heat Shock Protein 27 Signaling and Lipid Raft‐Mediated Endocytosis Negatively Regulated by Caveolin‐1.” Journal of Biological Chemistry 288, no. 24: 17713–17724. 10.1074/jbc.M112.445403. 23653359 PMC3682571

[acel70607-bib-0222] Swanson, J. A. 2008. “Shaping Cups Into Phagosomes and Macropinosomes.” Nature Reviews. Molecular Cell Biology 9, no. 8: 639–649. 10.1038/nrm2447. 18612320 PMC2851551

[acel70607-bib-0223] Tao, S. , M. Song , J. Fan , F. Zhu , T. Lv , and H. Wei . 2025. “ *Lactobacillus johnsonii* ‐Derived Extracellular Vesicles Carrying GAPDH Protect Against Ulcerative Colitis Through Modulating Macrophage Polarization.” Journal of Advanced Research 81: 409–424. 10.1016/j.jare.2025.06.035. 40533059 PMC12958211

[acel70607-bib-0224] Tesei, A. , C. Arienti , G. Bossi , et al. 2021. “TP53 Drives Abscopal Effect by Secretion of Senescence‐Associated Molecular Signals in Non‐Small Cell Lung Cancer.” Journal of Experimental & Clinical Cancer Research 40, no. 1: 89. 10.1186/s13046-021-01883-0. 33673859 PMC7934399

[acel70607-bib-0225] Thakur, N. S. , I. Rus , A. Herbert , et al. 2024. “Crosslinked‐Hybrid Nanoparticle Embedded in Thermogel for Sustained Co‐Delivery to Inner Ear.” Journal of Nanobiotechnology 22, no. 1: 482. 10.1186/s12951-024-02686-z. 39135039 PMC11321169

[acel70607-bib-0226] Tian, T. , Y. L. Zhu , F. H. Hu , Y. Y. Wang , N. P. Huang , and Z. D. Xiao . 2013. “Dynamics of Exosome Internalization and Trafficking.” Journal of Cellular Physiology 228, no. 7: 1487–1495. 10.1002/jcp.24304. 23254476

[acel70607-bib-0227] Tian, T. , Y. L. Zhu , Y. Y. Zhou , et al. 2014. “Exosome Uptake Through Clathrin‐Mediated Endocytosis and Macropinocytosis and Mediating miR‐21 Delivery.” Journal of Biological Chemistry 289, no. 32: 22258–22267. 10.1074/jbc.M114.588046. 24951588 PMC4139237

[acel70607-bib-0228] Tian, Y. , S. Li , J. Song , et al. 2014. “A Doxorubicin Delivery Platform Using Engineered Natural Membrane Vesicle Exosomes for Targeted Tumor Therapy.” Biomaterials 35, no. 7: 2383–2390. 10.1016/j.biomaterials.2013.11.083. 24345736

[acel70607-bib-0229] Tkach, M. , and C. Thery . 2016. “Communication by Extracellular Vesicles: Where we Are and Where we Need to Go.” Cell 164, no. 6: 1226–1232. 10.1016/j.cell.2016.01.043. 26967288

[acel70607-bib-0230] Todtenhaupt, P. , L. A. Franken , S. G. Groene , et al. 2023. “A Robust and Standardized Method to Isolate and Expand Mesenchymal Stromal Cells From Human Umbilical Cord.” Cytotherapy 25, no. 10: 1057–1068. 10.1016/j.jcyt.2023.07.004. 37516948

[acel70607-bib-0231] Tolomeo, A. M. , R. Malvicini , D. Ventrella , et al. 2024. “Protective Effects of Mesenchymal Stem Cells‐Derived Extracellular Vesicles Against Ischemia‐Reperfusion Injury of Hearts Donated After Circulatory Death: Preliminary Study in a Pig Model.” Biomedicine & Pharmacotherapy 178: 117256. 10.1016/j.biopha.2024.117256. 39111081

[acel70607-bib-0232] Toms, M. , L. Toualbi , P. V. Almeida , R. Harbottle , and M. Moosajee . 2023. “Successful Large Gene Augmentation of USH2A With Non‐Viral Episomal Vectors.” Molecular Therapy 31, no. 9: 2755–2766. 10.1016/j.ymthe.2023.06.012. 37337429 PMC10491995

[acel70607-bib-0233] Van den Broek, B. , C. Wuyts , A. Sisto , et al. 2022. “Oligodendroglia‐Derived Extracellular Vesicles Activate Autophagy via LC3B/BAG3 to Protect Against Oxidative Stress With an Enhanced Effect for HSPB8 Enriched Vesicles.” Cell Communication and Signaling 20, no. 1: 58. 10.1186/s12964-022-00863-x. 35513867 PMC9069805

[acel70607-bib-0234] van Poppelen, N. M. , N. Cassoux , J. A. Turunen , et al. 2024. “The Pediatric and Young Adult Choroidal and Ciliary Body Melanoma Genetic Study, A Survey by the European Ophthalmic Oncology Group.” Investigative Ophthalmology & Visual Science 65, no. 4: 12. 10.1167/iovs.65.4.12. PMC10996971 38573618

[acel70607-bib-0235] Vandergriff, A. , K. Huang , D. Shen , et al. 2018. “Targeting Regenerative Exosomes to Myocardial Infarction Using Cardiac Homing Peptide.” Theranostics 8, no. 7: 1869–1878. 10.7150/thno.20524. 29556361 PMC5858505

[acel70607-bib-0236] Villamizar, O. , S. A. Waters , T. Scott , N. Grepo , A. Jaffe , and K. V. Morris . 2021. “Mesenchymal Stem Cell Exosome Delivered Zinc Finger Protein Activation of Cystic Fibrosis Transmembrane Conductance Regulator.” Journal of Extracellular Vesicles 10, no. 3: e12053. 10.1002/jev2.12053. 33532041 PMC7825549

[acel70607-bib-0237] Villeda, S. A. , K. E. Plambeck , J. Middeldorp , et al. 2014. “Young Blood Reverses Age‐Related Impairments in Cognitive Function and Synaptic Plasticity in Mice.” Nature Medicine 20, no. 6: 659–663. 10.1038/nm.3569. PMC4224436 24793238

[acel70607-bib-0238] von Lersner, A. K. , F. Fernandes , P. M. M. Ozawa , et al. 2024. “Multiparametric Single‐Vesicle Flow Cytometry Resolves Extracellular Vesicle Heterogeneity and Reveals Selective Regulation of Biogenesis and Cargo Distribution.” ACS Nano 18, no. 15: 10464–10484. 10.1021/acsnano.3c11561. 38578701 PMC11025123

[acel70607-bib-0239] Wallis, R. , N. Josipovic , H. Mizen , et al. 2021. “Isolation Methodology Is Essential to the Evaluation of the Extracellular Vesicle Component of the Senescence‐Associated Secretory Phenotype.” Journal of Extracellular Vesicles 10, no. 4: e12041. 10.1002/jev2.12041. 33659050 PMC7892802

[acel70607-bib-0240] Wang, C. , J. Ju , C. Fu , et al. 2025. “Metabolically Engineered Extracellular Vesicles Released From a Composite Hydrogel Delivery System Regulate the Microenvironment for Periprosthetic Osteolysis Treatment.” Journal of Extracellular Vesicles 14, no. 6: e70098. 10.1002/jev2.70098. 40545972 PMC12183387

[acel70607-bib-0241] Wang, T. , H. Zhao , Y. Zhang , et al. 2024. “A Novel Extracellular Vesicles Production System Harnessing Matrix Homeostasis and Macrophage Reprogramming Mitigates Osteoarthritis.” Journal of Nanobiotechnology 22, no. 1: 79. 10.1186/s12951-024-02324-8. 38419097 PMC10903078

[acel70607-bib-0242] Wang, X. , W. Cheng , and J. Su . 2024. “Research Progress of Extracellular Vesicles‐Loaded Microneedle Technology.” Pharmaceutics 16, no. 3: 326. 10.3390/pharmaceutics16030326. 38543220 PMC10975918

[acel70607-bib-0243] Wang, Z. , Z. Guan , H. Wang , et al. 2024. “Pure ZrO_2_ Ferroelectric Thin Film for Nonvolatile Memory and Neural Network Computing.” ACS Applied Materials & Interfaces 16, no. 17: 22122–22130. 10.1021/acsami.4c01234. 38626418

[acel70607-bib-0246] Wei, D. , W. Zhan , Y. Gao , et al. 2021. “RAB31 Marks and Controls an ESCRT‐Independent Exosome Pathway.” Cell Research 31, no. 2: 157–177. 10.1038/s41422-020-00409-1. 32958903 PMC8027411

[acel70607-bib-0247] Welsh, J. A. , D. C. I. Goberdhan , L. O'Driscoll , et al. 2024. “Minimal Information for Studies of Extracellular Vesicles (MISEV2023): From Basic to Advanced Approaches.” Journal of Extracellular Vesicles 13, no. 2: e12404. 10.1002/jev2.12404. 38326288 PMC10850029

[acel70607-bib-0248] Werninghaus, I. C. , D. M. Hinke , E. Fossum , B. Bogen , and R. Braathen . 2023. “Neuraminidase Delivered as an APC‐Targeted DNA Vaccine Induces Protective Antibodies Against Influenza.” Molecular Therapy 31, no. 7: 2188–2205. 10.1016/j.ymthe.2023.03.012. 36926694 PMC10362400

[acel70607-bib-0249] Whiteside, T. L. , B. Diergaarde , and C. S. Hong . 2021. “Tumor‐Derived Exosomes (TEX) and Their Role in Immuno‐Oncology.” International Journal of Molecular Sciences 22, no. 12: 6234. 10.3390/ijms22126234. 34207762 PMC8229953

[acel70607-bib-0250] Wiklander, O. P. , J. Z. Nordin , A. O'Loughlin , et al. 2015. “Extracellular Vesicle In Vivo Biodistribution Is Determined by Cell Source, Route of Administration and Targeting.” Journal of Extracellular Vesicles 4: 26316. 10.3402/jev.v4.26316. 25899407 PMC4405624

[acel70607-bib-0251] Wilkinson, J. E. , L. Burmeister , S. V. Brooks , et al. 2012. “Rapamycin Slows Aging in Mice.” Aging Cell 11, no. 4: 675–682. 10.1111/j.1474-9726.2012.00832.x. 22587563 PMC3434687

[acel70607-bib-0252] Williams, C. , F. Royo , O. Aizpurua‐Olaizola , et al. 2018. “Glycosylation of Extracellular Vesicles: Current Knowledge, Tools and Clinical Perspectives.” Journal of Extracellular Vesicles 7, no. 1: 1442985. 10.1080/20013078.2018.1442985. 29535851 PMC5844028

[acel70607-bib-0253] Witwer, K. W. , B. W. M. Van Balkom , S. Bruno , et al. 2019. “Defining Mesenchymal Stromal Cell (MSC)‐Derived Small Extracellular Vesicles for Therapeutic Applications.” Journal of Extracellular Vesicles 8, no. 1: 1609206. 10.1080/20013078.2019.1609206. 31069028 PMC6493293

[acel70607-bib-0254] Wong, C. W. Y. , D. S. F. Yu , P. W. C. Li , and B. S. Chan . 2023. “The Prognostic Impacts of Frailty on Clinical and Patient‐Reported Outcomes in Patients Undergoing Coronary Artery or Valvular Surgeries/Procedures: A Systematic Review and Meta‐Analysis.” Ageing Research Reviews 85: 101850. 10.1016/j.arr.2023.101850. 36640867

[acel70607-bib-0255] Wozniak, O. , B. Mierzejewski , and E. Brzoska . 2025. “MicroRNA‐126: A Key Regulator of Angiogenesis, Inflammation, and Tumorigenesis‐Exploring Its Multifaceted Functions in Vascular Health and Cancer.” Biochimica et Biophysica Acta‐Molecular Basis of Disease 1871, no. 8: 167984. 10.1016/j.bbadis.2025.167984. 40651584

[acel70607-bib-0256] Wu, G. , T. Su , P. Zhou , et al. 2025. “Engineering M2 Macrophage‐Derived Exosomes Modulate Activated T Cell Cuproptosis to Promote Immune Tolerance in Rheumatoid Arthritis.” Biomaterials 315: 122943. 10.1016/j.biomaterials.2024.122943. 39509857

[acel70607-bib-0257] Wu, S. , T. Yang , M. Ma , et al. 2024. “Extracellular Vesicles Meet Mitochondria: Potential Roles in Regenerative Medicine.” Pharmacological Research 206: 107307. 10.1016/j.phrs.2024.107307. 39004243

[acel70607-bib-0258] Wu, Y. , Y. Feng , F. Hu , et al. 2025. “Engineered Stem Cell Clusters for Extracellular Vesicles‐Mediated Gene Delivery to Rejuvenate Chondrocytes and Facilitate Chondrogenesis in Osteoarthritis Therapy.” Advanced Science 12, no. 25: e2500964. 10.1002/advs.202500964. 40278049 PMC12224965

[acel70607-bib-0259] Xia, L. , C. Zhang , N. Lv , et al. 2022. “AdMSC‐Derived Exosomes Alleviate Acute Lung Injury via Transferring Mitochondrial Component to Improve Homeostasis of Alveolar Macrophages.” Theranostics 12, no. 6: 2928–2947. 10.7150/thno.69533. 35401830 PMC8965475

[acel70607-bib-0260] Xia, Y. , L. Rao , H. Yao , Z. Wang , P. Ning , and X. Chen . 2020. “Engineering Macrophages for Cancer Immunotherapy and Drug Delivery.” Advanced Materials 32, no. 40: e2002054. 10.1002/adma.202002054. 32856350

[acel70607-bib-0261] Xiao, X. , M. Xu , H. Yu , et al. 2021. “Mesenchymal Stem Cell‐Derived Small Extracellular Vesicles Mitigate Oxidative Stress‐Induced Senescence in Endothelial Cells via Regulation of miR‐146a/Src.” Signal Transduction and Targeted Therapy 6, no. 1: 354. 10.1038/s41392-021-00765-3. 34675187 PMC8531331

[acel70607-bib-0262] Xie, X. , Q. Song , C. Dai , et al. 2023. “Clinical Safety and Efficacy of Allogenic Human Adipose Mesenchymal Stromal Cells‐Derived Exosomes in Patients With Mild to Moderate Alzheimer's Disease: A Phase I/II Clinical Trial.” General Psychiatry 36, no. 5: e101143. 10.1136/gpsych-2023-101143. 37859748 PMC10582850

[acel70607-bib-0263] Xiong, S. , H. Xiao , M. Sun , et al. 2023. “Glutamate‐Releasing BEST1 Channel Is a New Target for Neuroprotection Against Ischemic Stroke With Wide Time Window.” Acta Pharmaceutica Sinica B 13, no. 7: 3008–3026. 10.1016/j.apsb.2023.05.012. 37521872 PMC10372917

[acel70607-bib-0264] Xiong, Y. , Q. Zhang , J. Li , et al. 2024. “Light‐Sensitive PEG Hydrogel With Antibacterial Performance for Pacemaker Pocket Infection Prevention.” Materials Today Bio 25: 100987. 10.1016/j.mtbio.2024.100987. PMC10938169 38486799

[acel70607-bib-0265] Xu, P. , Y. Xin , Z. Zhang , et al. 2020. “Extracellular Vesicles From Adipose‐Derived Stem Cells Ameliorate Ultraviolet B‐Induced Skin Photoaging by Attenuating Reactive Oxygen Species Production and Inflammation.” Stem Cell Research & Therapy 11, no. 1: 264. 10.1186/s13287-020-01777-6. 32611371 PMC7329484

[acel70607-bib-0266] Yan, Q. , H. Liu , S. Sun , et al. 2024. “Adipose‐Derived Stem Cell Exosomes Loaded With Icariin Alleviates Rheumatoid Arthritis by Modulating Macrophage Polarization in Rats.” Journal of Nanobiotechnology 22, no. 1: 423. 10.1186/s12951-024-02711-1. 39026367 PMC11256651

[acel70607-bib-0267] Yan, T. , L. Huang , Y. Yan , Y. Zhong , H. Xie , and X. Wang . 2023. “Bone Marrow Mesenchymal Stem Cell‐Derived Exosome miR‐29b‐3p Alleviates UV Irradiation‐Induced Photoaging in Skin Fibroblast.” Photodermatology, Photoimmunology & Photomedicine 39, no. 3: 235–245. 10.1111/phpp.12827. 35950642

[acel70607-bib-0268] Yang, J. , Y. Li , X. Fan , et al. 2025. “Collagen Glycation‐Mediated Mechanical Stress Aggravates Ischemia‐Reperfusion Injury.” Acta Biomaterialia 203: 412–426. 10.1016/j.actbio.2025.06.012. 40484296

[acel70607-bib-0269] Yang, L. , X. Peng , Y. Li , et al. 2019. “Long Non‐Coding RNA HOTAIR Promotes Exosome Secretion by Regulating RAB35 and SNAP23 in Hepatocellular Carcinoma.” Molecular Cancer 18, no. 1: 78. 10.1186/s12943-019-0990-6. 30943982 PMC6446409

[acel70607-bib-0270] Yang, Y. , Y. Qiu , S. Xu , et al. 2025. “Bioactive Vascular Buds Promote Collateral Vessel Formation by Grafting on the Artificial Vessel Walls.” Bioactive Materials 49: 564–575. 10.1016/j.bioactmat.2025.03.015. 40212784 PMC11982305

[acel70607-bib-0271] Ye, J. , X. Sun , Q. Jiang , et al. 2024. “Umbilical Cord Blood‐Derived Exosomes Attenuate Dopaminergic Neuron Damage of Parkinson's Disease Mouse Model.” Journal of Nanobiotechnology 22, no. 1: 567. 10.1186/s12951-024-02773-1. 39277761 PMC11401276

[acel70607-bib-0272] Ye, P. , Z. Mi , D. Wei , P. Gao , M. Ma , and H. Yang . 2022. “miR‐3960 From Mesenchymal Stem Cell‐Derived Extracellular Vesicles Inactivates SDC1/Wnt/Beta‐Catenin Axis to Relieve Chondrocyte Injury in Osteoarthritis by Targeting PHLDA2.” Stem Cells International 2022: 9455152. 10.1155/2022/9455152. 36061148 PMC9438433

[acel70607-bib-0273] Yin, D. , Q. Pang , Y. Yuan , et al. 2025. “An In Vivo Target Mutagenesis System for Multiple Hosts.” Trends in Biotechnology 43, no. 8: 2049–2072. 10.1016/j.tibtech.2025.04.005. 40345898

[acel70607-bib-0274] Yoshida, M. , A. Satoh , J. B. Lin , et al. 2019. “Extracellular Vesicle‐Contained eNAMPT Delays Aging and Extends Lifespan in Mice.” Cell Metabolism 30, no. 2: 329–342. 10.1016/j.cmet.2019.05.015. 31204283 PMC6687560

[acel70607-bib-0275] You, Y. , Y. Tian , R. Guo , et al. 2025. “Extracellular Vesicle‐Mediated VEGF‐A mRNA Delivery Rescues Ischaemic Injury With Low Immunogenicity.” European Heart Journal 46, no. 17: 1662–1676. 10.1093/eurheartj/ehae883. 39831819

[acel70607-bib-0276] Yousef, H. , C. J. Czupalla , D. Lee , et al. 2019. “Aged Blood Impairs Hippocampal Neural Precursor Activity and Activates Microglia via Brain Endothelial Cell VCAM1.” Nature Medicine 25, no. 6: 988–1000. 10.1038/s41591-019-0440-4. PMC6642642 31086348

[acel70607-bib-0277] Yu, A. , Y. Zhang , S. Zhong , Z. Yang , and M. Xie . 2025. “Human Umbilical Cord Mesenchymal Stem Cell‐Derived Exosomes Enhance Follicular Regeneration in Androgenetic Alopecia via Activation of Wnt/Beta‐Catenin Pathway.” Stem Cell Research & Therapy 16, no. 1: 418. 10.1186/s13287-025-04538-5. 40751216 PMC12317459

[acel70607-bib-0278] Yu, F. , X. Zhao , Q. Wang , et al. 2024. “Engineered Mesenchymal Stromal Cell Exosomes‐Loaded Microneedles Improve Corneal Healing After Chemical Injury.” ACS Nano 18: 20065–20082. 10.1021/acsnano.4c00423. 39047084

[acel70607-bib-0279] Yu, H. 2021. “Atherosclerotic Plaque Regression: Experimental Approaches and Therapeutic Advances.” Trends in Cell Biology 31, no. 6: 424–427. 10.1016/j.tcb.2021.03.003. 33726967

[acel70607-bib-0280] Yu, J. , S. Sane , J. E. Kim , et al. 2023. “Biogenesis and Delivery of Extracellular Vesicles: Harnessing the Power of EVs for Diagnostics and Therapeutics.” Frontiers in Molecular Biosciences 10: 1330400. 10.3389/fmolb.2023.1330400. 38234582 PMC10791869

[acel70607-bib-0281] Yu, Q. , J. Wang , C. Liang , et al. 2024. “A Giant Magneto‐Superelasticity of 5% Enabled by Introducing Ordered Dislocations in Ni(34)co(8)cu(8)Mn(36)Ga(14) Single Crystal.” Advanced Science 11, no. 25: e2401234. 10.1002/advs.202401234. 38654685 PMC11220696

[acel70607-bib-0282] Yu, S. , Q. Cheng , Q. Yu , et al. 2026. “Systemic LINE‐1 RNA in Plasma Extracellular Vesicles Drives Neuroinflammation and Cognitive Dysfunction via cGAS‐STING Pathway in Aging.” Aging Cell 25, no. 1: e70350. 10.1111/acel.70350. 41480826 PMC12757926

[acel70607-bib-0283] Yuan, Q. , M. Yang , H. Zheng , et al. 2024. “M2 Macrophage‐Derived Extracellular Vesicles Encapsulated in Hyaluronic Acid Alleviate Osteoarthritis by Modulating Macrophage Polarization.” ACS Biomaterials Science & Engineering 10, no. 5: 3355–3377. 10.1021/acsbiomaterials.3c01833. 38563817

[acel70607-bib-0284] Yuan, Y. , K. Cao , P. Gao , Y. Wang , W. An , and Y. Dong . 2025. “Extracellular Vesicles and Bioactive Peptides for Regenerative Medicine in Cosmetology.” Ageing Research Reviews 107: 102712. 10.1016/j.arr.2025.102712. 40032214

[acel70607-bib-0285] Yuan, Z. , D. Jiang , M. Yang , et al. 2024. “Emerging Roles of Macrophage Polarization in Osteoarthritis: Mechanisms and Therapeutic Strategies.” Orthopaedic Surgery 16, no. 3: 532–550. 10.1111/os.13993. 38296798 PMC10925521

[acel70607-bib-0286] Zhang, A. , Q. Li , and Z. Chen . 2024. “Therapeutic Efficacy and Promise of Human Umbilical Cord Mesenchymal Stem Cell‐Derived Extracellular Vesicles in Aging and Age‐Related Disorders.” International Journal of Molecular Sciences 26, no. 1: 225. 10.3390/ijms26010225. 39796081 PMC11719504

[acel70607-bib-0287] Zhang, D. , J. W. Zhang , H. Xu , et al. 2024. “Therapy‐Induced Senescent Tumor Cell‐Derived Extracellular Vesicles Promote Colorectal Cancer Progression Through SERPINE1‐Mediated NF‐kappaB p65 Nuclear Translocation.” Molecular Cancer 23, no. 1: 70. 10.1186/s12943-024-01985-1. 38576002 PMC10993572

[acel70607-bib-0288] Zhang, J. , S. He , Q. Xiao , et al. 2025. “Engineered Mesenchymal Stem Cell‐Derived Extracellular Vesicles Overexpressing miR‐146a Alleviate Neuroinflammation in Alzheimer's Disease.” Neural Regeneration Research: 10–4103. 10.4103/NRR.NRR-D-25-00404. PMC13568608 41017725

[acel70607-bib-0289] Zhang, S. , Z. Y. Zhang , B. D. Sui , C. X. Zheng , and Y. Fu . 2025. “The Epigenetic Landscape of Mesenchymal Stem Cell and Extracellular Vesicle Therapy.” Trends in Cell Biology 36: 42–56. 10.1016/j.tcb.2025.03.008. 40300990

[acel70607-bib-0291] Zhang, T. , A. D. C. Nunes , J. Lee , et al. 2025. “Identification of Senomorphic miRNAs in Embryonic Progenitor and Adult Stem Cell‐Derived Extracellular Vesicles.” Aging Cell 24, no. 7: e70071. 10.1111/acel.70071. 40275616 PMC12266766

[acel70607-bib-0292] Zhang, W. , K. Fukazawa , A. Mahara , H. Jiang , and T. Yamaoka . 2024. “Photo‐Induced Universal Modification of Small‐Diameter Decellularized Blood Vessels With a Hemocompatible Peptide Improves In Vivo Patency.” Acta Biomaterialia 176: 116–127. 10.1016/j.actbio.2024.01.012. 38232911

[acel70607-bib-0293] Zhang, W. , X. Zhang , Z. Zhou , et al. 2025. “Engineering Natural Killer Cell‐Derived Extracellular Vesicles for Disease Therapy.” Journal of Controlled Release 390: 114535. 10.1016/j.jconrel.2025.114535. 41389966

[acel70607-bib-0294] Zhang, X. , T. Liu , X. Hou , et al. 2023. “Exosomes Secreted by Mesenchymal Stem Cells Delay Brain Aging by Upregulating SIRT1 Expression.” Scientific Reports 13, no. 1: 13213. 10.1038/s41598-023-40543-5. 37580391 PMC10425430

[acel70607-bib-0295] Zhang, Y. , Z. Li , H. Guan , Z. Qiu , and C. Zou . 2025. “Engineering Exosomes for Alzheimer's Disease: Multi‐Target Therapeutic Strategies From Pathogenesis to Clinical Translation.” Clinical and Translational Medicine 15, no. 12: e70548. 10.1002/ctm2.70548. 41399181 PMC12706182

[acel70607-bib-0296] Zhang, Z. , S. Ai , Z. Yang , and X. Li . 2021. “Peptide‐Based Supramolecular Hydrogels for Local Drug Delivery.” Advanced Drug Delivery Reviews 174: 482–503. 10.1016/j.addr.2021.05.010. 34015417

[acel70607-bib-0297] Zhang, Z. , R. Ji , Z. Liu , et al. 2025. “hUMSC‐Exosomes Suppress TREM1‐p38 MAPK Signaling via HMGB1‐Dependent Mechanisms to Reprogram Microglial Function and Promote Neuroprotection in Ischemic Stroke.” Journal of Nanobiotechnology 23, no. 1: 572. 10.1186/s12951-025-03652-z. 40830888 PMC12363081

[acel70607-bib-0298] Zhao, M. , S. Liu , C. Wang , et al. 2021. “Mesenchymal Stem Cell‐Derived Extracellular Vesicles Attenuate Mitochondrial Damage and Inflammation by Stabilizing Mitochondrial DNA.” ACS Nano 15, no. 1: 1519–1538. 10.1021/acsnano.0c08947. 33369392

[acel70607-bib-0299] Zhao, R. , L. Wang , T. Wang , P. Xian , H. Wang , and Q. Long . 2022. “Inhalation of MSC‐EVs Is a Noninvasive Strategy for Ameliorating Acute Lung Injury.” Journal of Controlled Release 345: 214–230. 10.1016/j.jconrel.2022.03.025. 35307508

[acel70607-bib-0300] Zhao, S. , J. Li , X. Xing , J. Chen , Q. Zhou , and J. Sun . 2023. “Oxyberberine Suppressed the Carbon Tetrachloride‐Induced Liver Fibrosis by Inhibiting Liver Inflammation in a Sirtuin 3‐Dependent Manner in Mice.” International Immunopharmacology 116: 109876. 10.1016/j.intimp.2023.109876. 37599565

[acel70607-bib-0301] Zheng, P. , L. Chen , X. Yuan , et al. 2017. “Exosomal Transfer of Tumor‐Associated Macrophage‐Derived miR‐21 Confers Cisplatin Resistance in Gastric Cancer Cells.” Journal of Experimental & Clinical Cancer Research 36, no. 1: 53. 10.1186/s13046-017-0528-y. 28407783 PMC5390430

[acel70607-bib-0302] Zheng, Q. , S. Wang , T. Wang , and G. Zhang . 2025. “Efficacy of Stem Cell‐Derived Extracellular Vesicles in the Treatment of Alzheimer's Disease Model Mice: A Systematic Review and Meta‐Analysis.” Current Stem Cell Research & Therapy 20, no. 7: 728–747. 10.2174/011574888X352270250407170235. 40257023

[acel70607-bib-0303] Zheng, X. , W. Wang , S. Chen , B. Zuo , and J. Li . 2023. “Transplanted Mesenchymal Stromal Cells Are Unable to Migrate to the Bone Surface and Subsequently Improve Osteogenesis in Glucocorticoid‐Induced Osteoporosis.” Cytotherapy 25, no. 5: 472–482. 10.1016/j.jcyt.2023.01.004. 36863932

[acel70607-bib-0304] Zhong, Y. , S. Du , and Y. Dong . 2023. “mRNA Delivery in Cancer Immunotherapy.” Acta Pharmaceutica Sinica B 13, no. 4: 1348–1357. 10.1016/j.apsb.2023.03.001. 37139419 PMC10150179

[acel70607-bib-0305] Zhou, G. , R. Li , S. Sheng , et al. 2024. “Organoids and Organoid Extracellular Vesicles‐Based Disease Treatment Strategies.” Journal of Nanobiotechnology 22, no. 1: 679. 10.1186/s12951-024-02917-3. 39506799 PMC11542470

[acel70607-bib-0306] Zhou, Q. L. , Y. Z. Bai , J. Gao , et al. 2021. “Human Serum‐Derived Extracellular Vesicles Protect A549 From PM (2.5)‐Induced Cell Apoptosis.” Biomedical and Environmental Sciences 34, no. 1: 40–49. 10.3967/bes2021.006. 33531106

[acel70607-bib-0307] Zhou, X. , S. Liu , Y. Lu , M. Wan , J. Cheng , and J. Liu . 2023. “MitoEVs: A New Player in Multiple Disease Pathology and Treatment.” Journal of Extracellular Vesicles 12, no. 4: e12320. 10.1002/jev2.12320. 37002588 PMC10065981

[acel70607-bib-0308] Zhu, B. , L. Zhang , C. Liang , et al. 2019. “Stem Cell‐Derived Exosomes Prevent Aging‐Induced Cardiac Dysfunction Through a Novel Exosome/lncRNA MALAT1/NF‐kappaB/TNF‐Alpha Signaling Pathway.” Oxidative Medicine and Cellular Longevity 2019: 9739258. 10.1155/2019/9739258. 31089420 PMC6476062

[acel70607-bib-0309] Zhu, S. , Y. Chen , H. Lin , et al. 2025. “SenExo‐cCCT2 Reprograms Senescence Response and Anti‐Tumor Immunity Following FOLFIRINOX Chemotherapy in Pancreatic Ductal Adenocarcinoma.” Advanced Science 12, no. 38: e08431. 10.1002/advs.202508431. 40686389 PMC12520514

[acel70607-bib-0310] Zhu, Y. G. , M. M. Shi , A. Monsel , et al. 2022. “Nebulized Exosomes Derived From Allogenic Adipose Tissue Mesenchymal Stromal Cells in Patients With Severe COVID‐19: A Pilot Study.” Stem Cell Research & Therapy 13, no. 1: 220. 10.1186/s13287-022-02900-5. 35619189 PMC9135389

[acel70607-bib-0311] Zhuang, Y. , S. Jiang , X. Deng , et al. 2024. “Energy Metabolism as Therapeutic Target for Aged Wound Repair by Engineered Extracellular Vesicle.” Science Advances 10, no. 15: eadl0372. 10.1126/sciadv.adl0372. 38608014 PMC11014449

[acel70607-bib-0312] Ziegler, J. N. , and C. Tian . 2023. “Engineered Extracellular Vesicles: Emerging Therapeutic Strategies for Translational Applications.” International Journal of Molecular Sciences 24, no. 20: 15206. 10.3390/ijms242015206. 37894887 PMC10607082

[acel70607-bib-0313] Ziglari, T. , N. L. Calistri , J. M. Finan , et al. 2025. “Senescent Cell‐Derived Extracellular Vesicles Inhibit Cancer Recurrence by Coordinating Immune Surveillance.” Cancer Research 85, no. 5: 859–874. 10.1158/0008-5472.CAN-24-0875. 39804967 PMC11878441

[acel70607-bib-0314] Zlotnick, H. M. , M. M. Stevens , and R. L. Mauck . 2024. “Physical‐Property‐Based Patterning: Simply Engineering Complex Tissues.” Trends in Biotechnology 42, no. 10: 1230–1240. 10.1016/j.tibtech.2024.03.004. 38664141 PMC11449661

